# Supplementary material for: Neural precursor cells rescue symptoms of Rett syndrome by activation of the Interferon γ pathway
Source: EMBO Mol Med. 2024 Sep 20;16(12):3218–46. doi: 10.1038/s44321-024-00144-9 (PMC11628625; doi:10.1038/s44321-024-00144-9)
Supplement: Supplementary file 1 — Appendix [file 44321_2024_144_MOESM1_ESM.pdf]

Table of Content of the RNA-seq Quality check:
Appendix Figure S1 (p. 1-2)

Appendix Figure S1

A) Table reporting sample IDs, genotypes and transplantation conditions of the sequenced samples.

B) Number of retained reads after Trimmomatic adapter trimming.

C) Unique versus duplicated number of reads

D) STAR alignment scores: number of uniquely mapped reads, mapped to many loci or unmapped.

E) Number of reads assigned to genes by HTSeq.

A

sampleID	1540	1551	1553	1584	1586	1595	1596	1597	1598	1599	1601	1607	1608	1609	1611	1612	1613	1614	1615	1621	1645
genotype	KO	WT	WT	KO	WT	WT	WT	WT	WT	KO	KO	KO	KO	KO	KO	WT	WT	WT	WT	KO	KO
condition	PBS	PBS	PBS	NPC	PBS	PBS	PBS	NPC	NPC	NPC	PBS	NPC	NPC	NPC	PBS	PBS	PBS	NPC	NPC	NPC	PBS

B

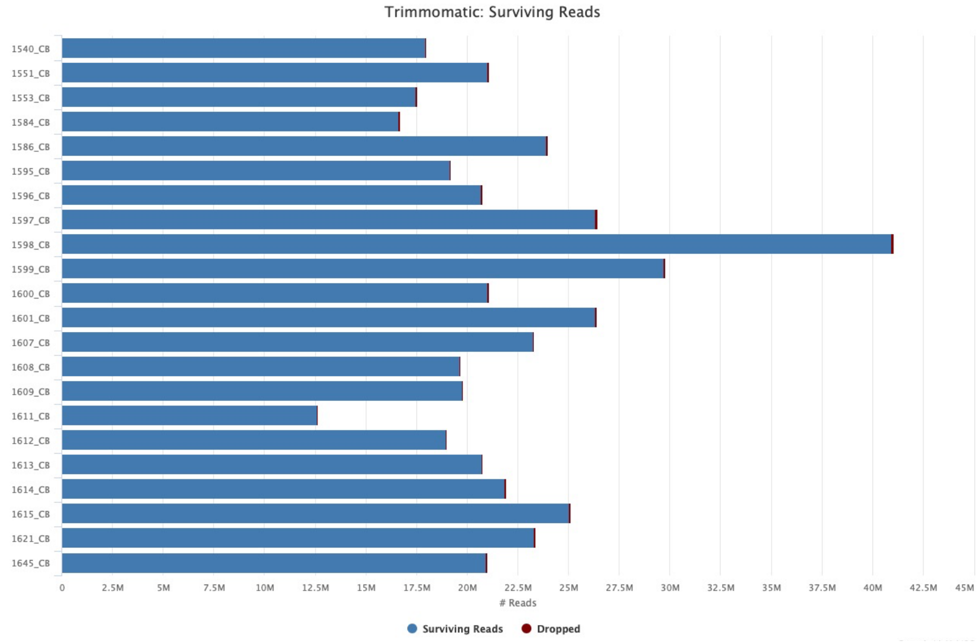

C

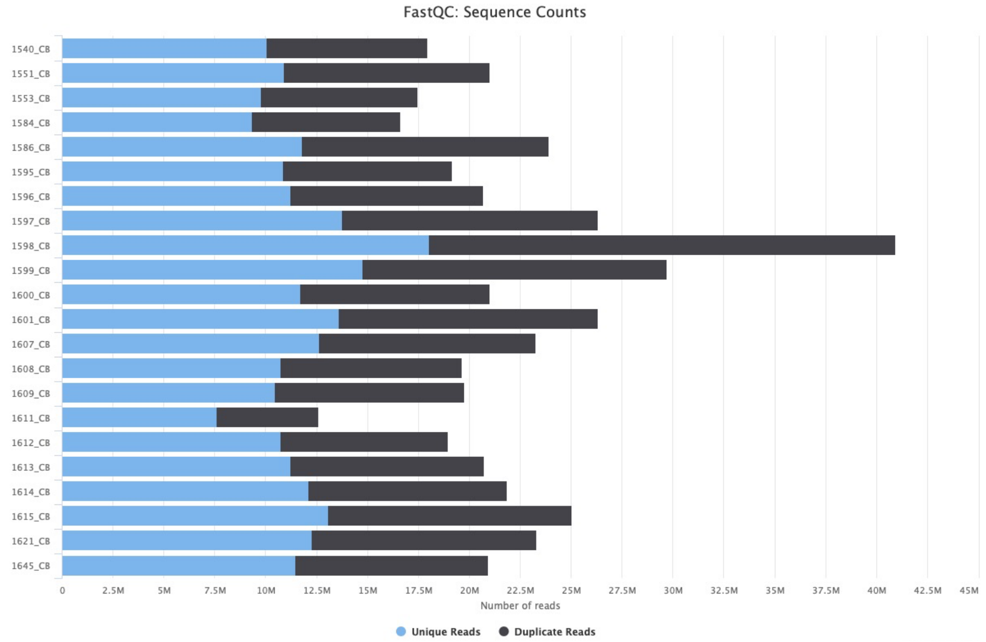

D

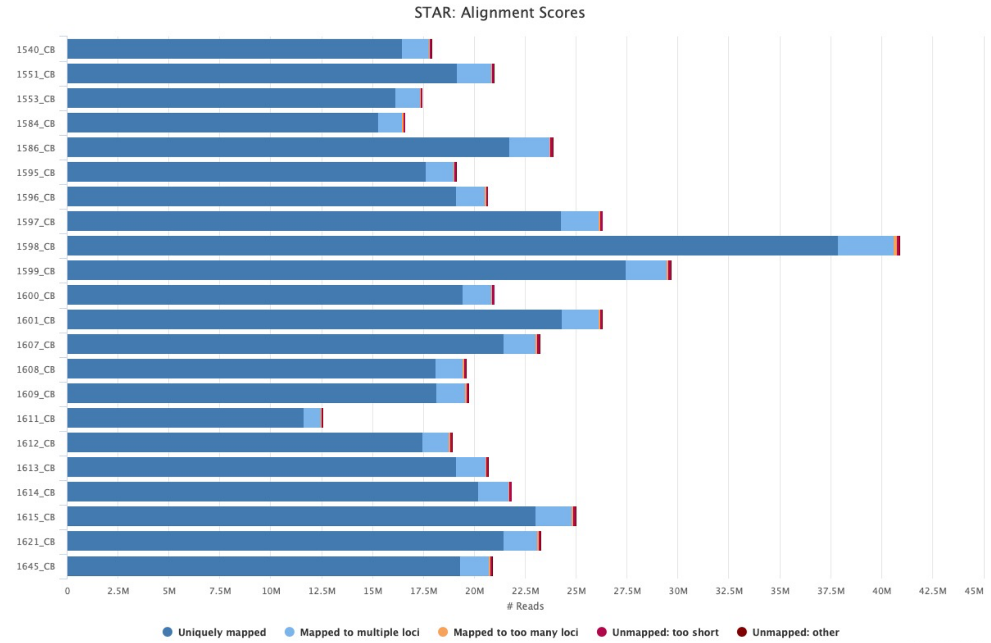
STAR: Alignment Scores
1540_CB
1551_CB
1553_CB
1584_CB
1586_CB
1595_CB
1596_CB
1597_CB
1598_CB
1599_CB
1600_CB
1601_CB
1607_CB
1608_CB
1609_CB
1611_CB
1612_CB
1613_CB
1614_CB
1615_CB
1621_CB
1645_CB
0
2.5M
5M
7.5M
10M
12.5M
15M
17.5M
20M
22.5M
25M
27.5M
30M
32.5M
35M
37.5M
40M
42.5M
45M
Reads
Uniquely mapped
Mapped to multiple loci
Mapped to too many loci
Unmapped: too short
Unmapped: other

E

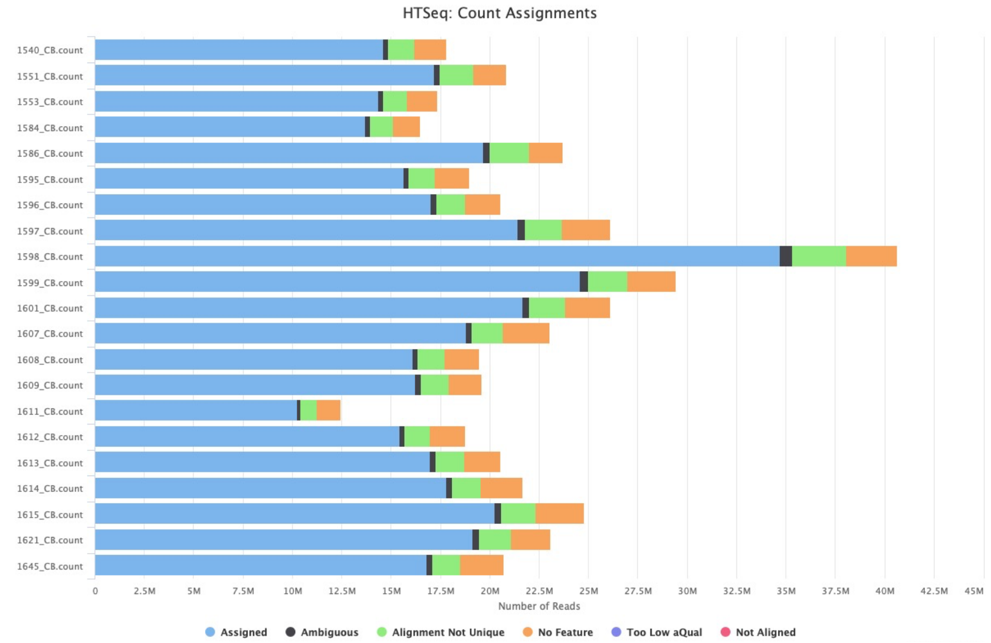
HTSeq: Count Assignments
1540_CB.count
1551_CB.count
1553_CB.count
1584_CB.count
1586_CB.count
1595_CB.count
1596_CB.count
1597_CB.count
1598_CB.count
1599_CB.count
1601_CB.count
1607_CB.count
1608_CB.count
1609_CB.count
1611_CB.count
1612_CB.count
1613_CB.count
1614_CB.count
1615_CB.count
1621_CB.count
1645_CB.count
0
2.5M
5M
7.5M
10M
12.5M
15M
17.5M
20M
22.5M
25M
27.5M
30M
32.5M
35M
37.5M
40M
42.5M
45M
Number of Reads
Assigned
Ambiguous
Alignment Not Unique
No Feature
Too Low aQual
Not Aligned
