# Supplementary figures and images for: Neural precursor cells rescue symptoms of Rett syndrome by activation of the Interferon γ pathway (part 1 of 2)
Source: EMBO Mol Med. 2024 Sep 20;16(12):3218–46. doi: 10.1038/s44321-024-00144-9 (PMC11628625; doi:10.1038/s44321-024-00144-9)

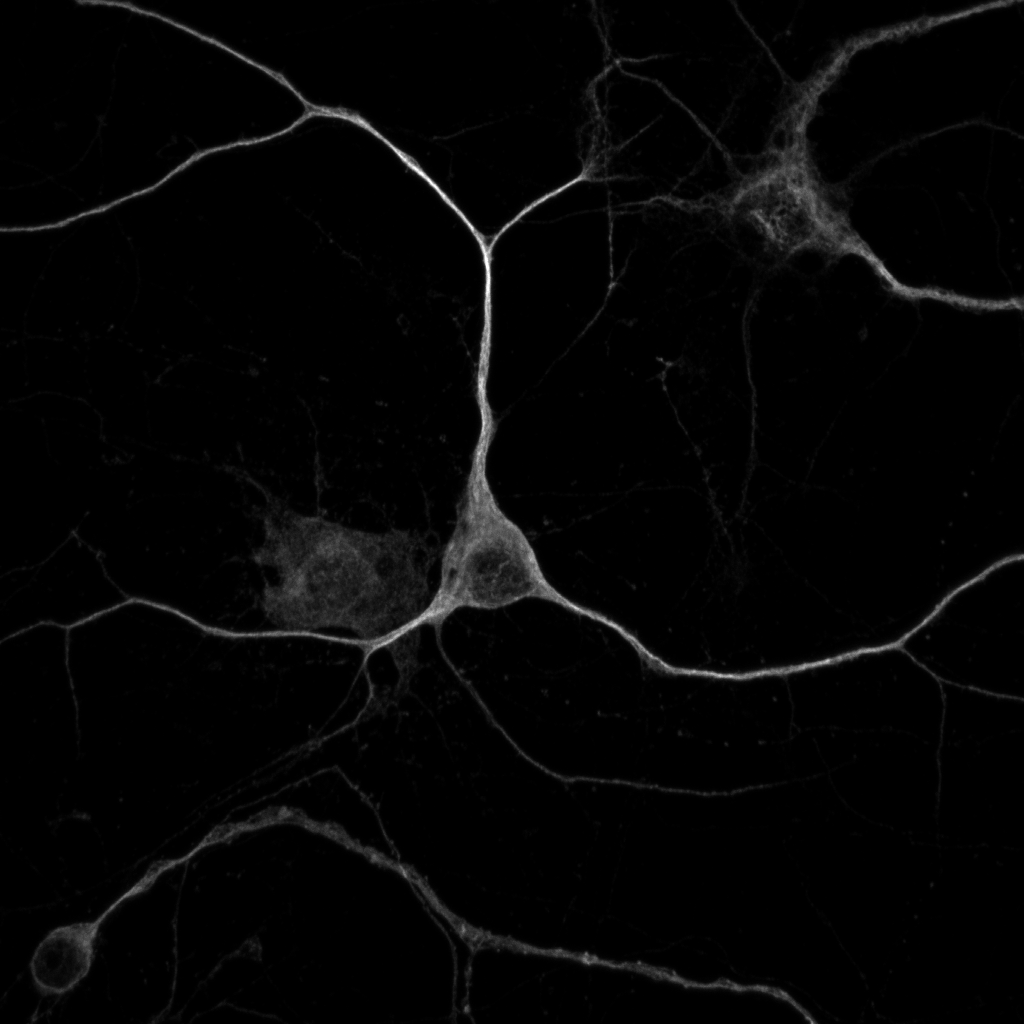

Supplement: Supplementary file 3 — Source data Fig. 1 [file 44321_2024_144_MOESM3_ESM.zip › Figure 1/1E/KO_3T3/MAP2_KO_3T3.tif]

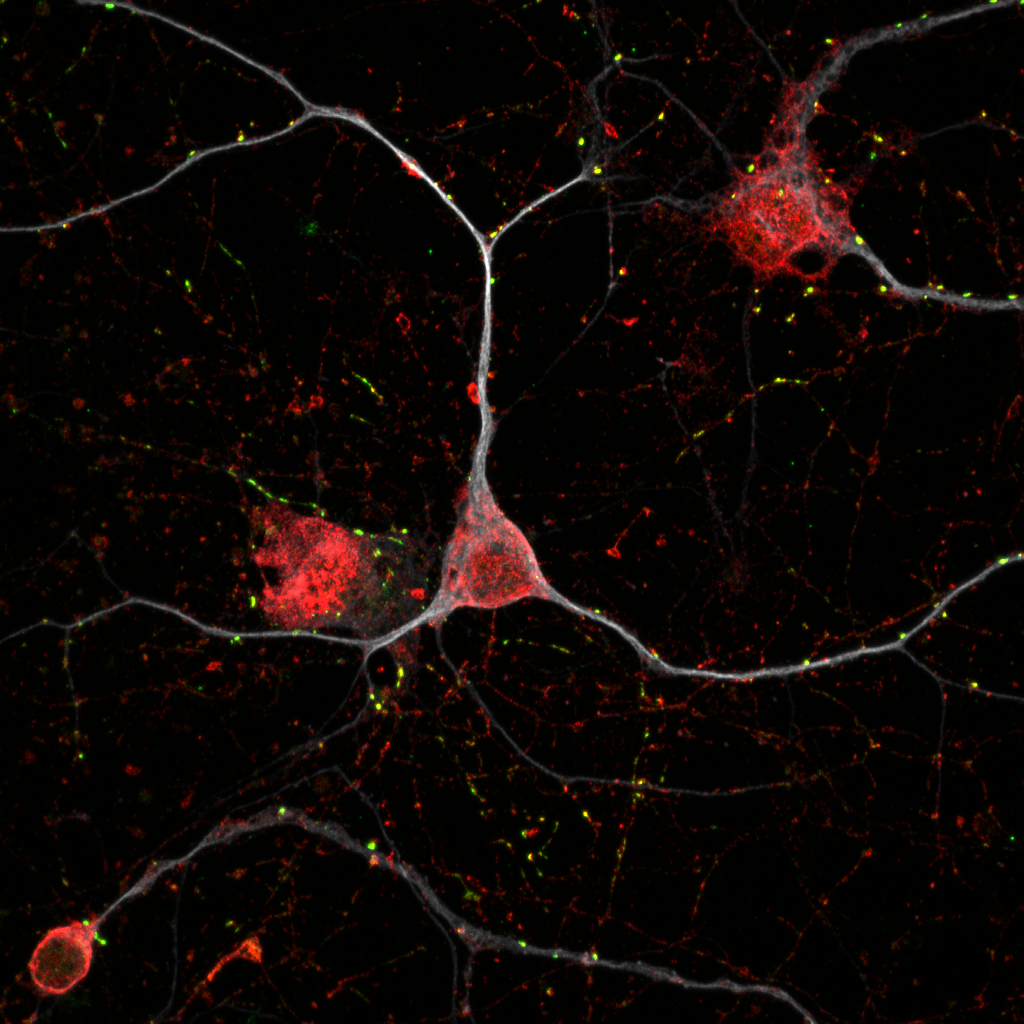

Supplement: Supplementary file 3 — Source data Fig. 1 [file 44321_2024_144_MOESM3_ESM.zip › Figure 1/1E/KO_3T3/MERGE_KO_3T3.tif]

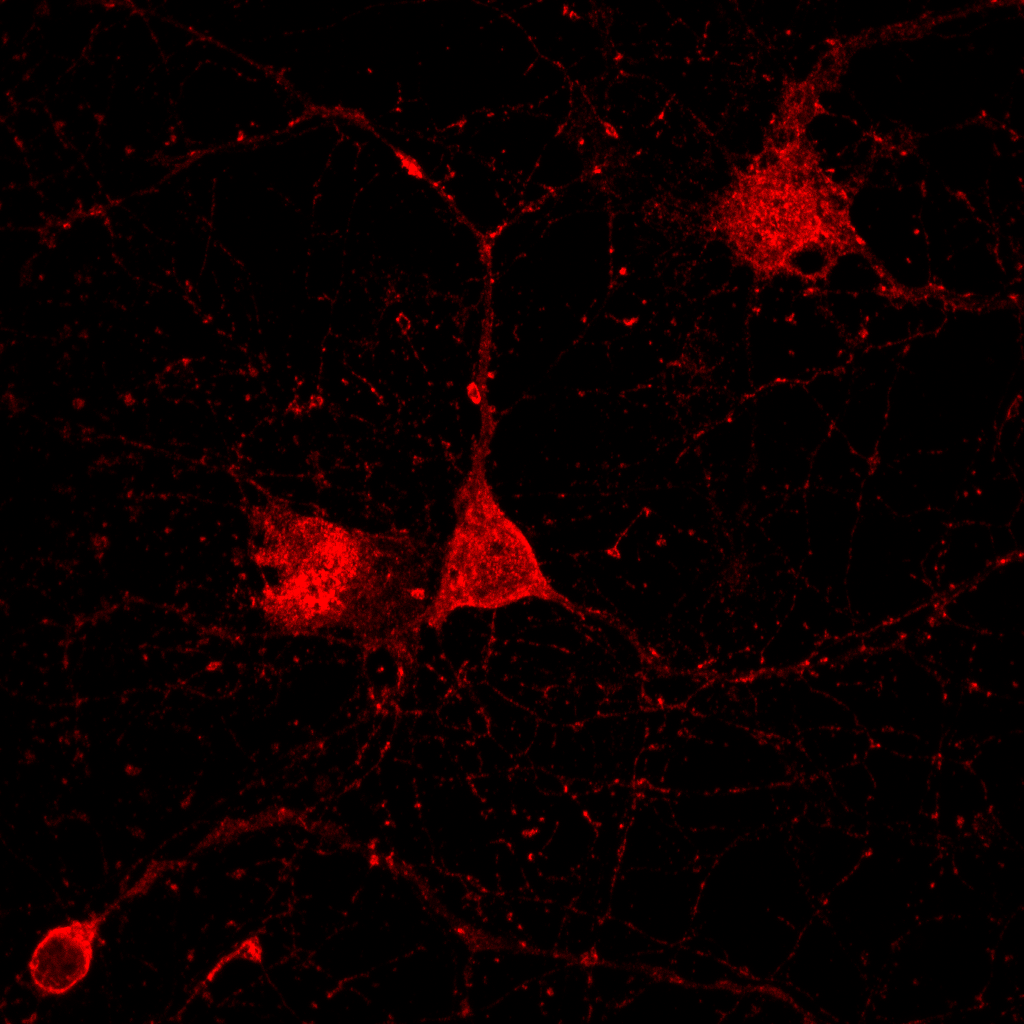

Supplement: Supplementary file 3 — Source data Fig. 1 [file 44321_2024_144_MOESM3_ESM.zip › Figure 1/1E/KO_3T3/SHANK_KO_3T3.tif]

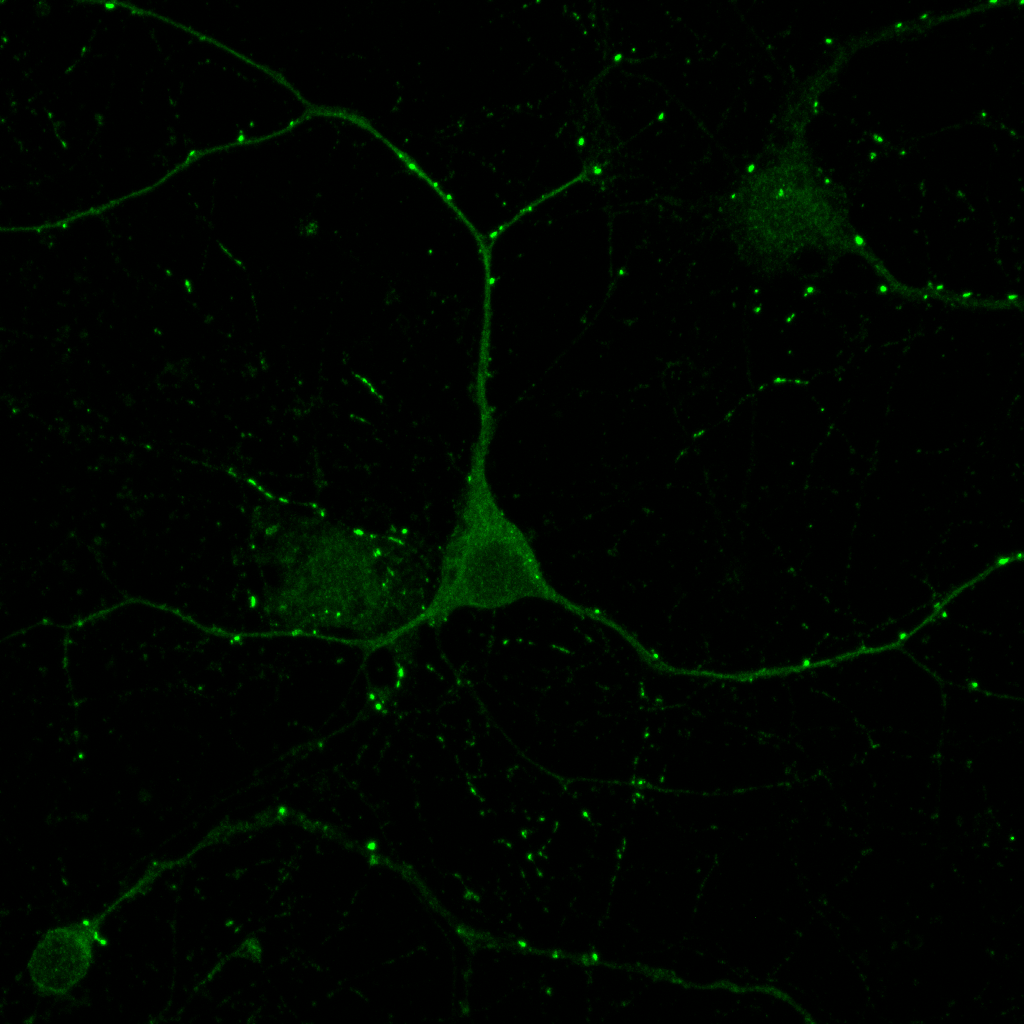

Supplement: Supplementary file 3 — Source data Fig. 1 [file 44321_2024_144_MOESM3_ESM.zip › Figure 1/1E/KO_3T3/SYN_KO_3T3.tif]

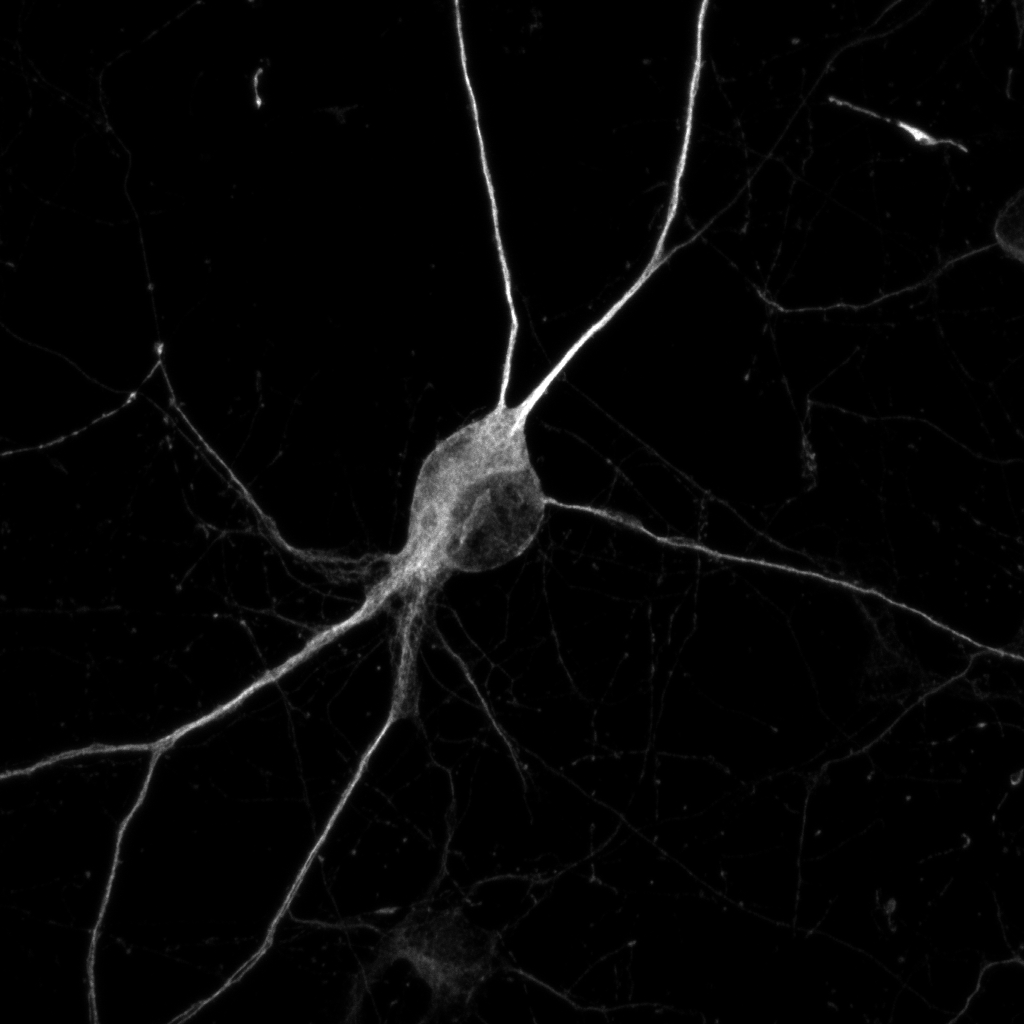

Supplement: Supplementary file 3 — Source data Fig. 1 [file 44321_2024_144_MOESM3_ESM.zip › Figure 1/1E/KO_NPCs/MAP2_KO_NPCs.tif]

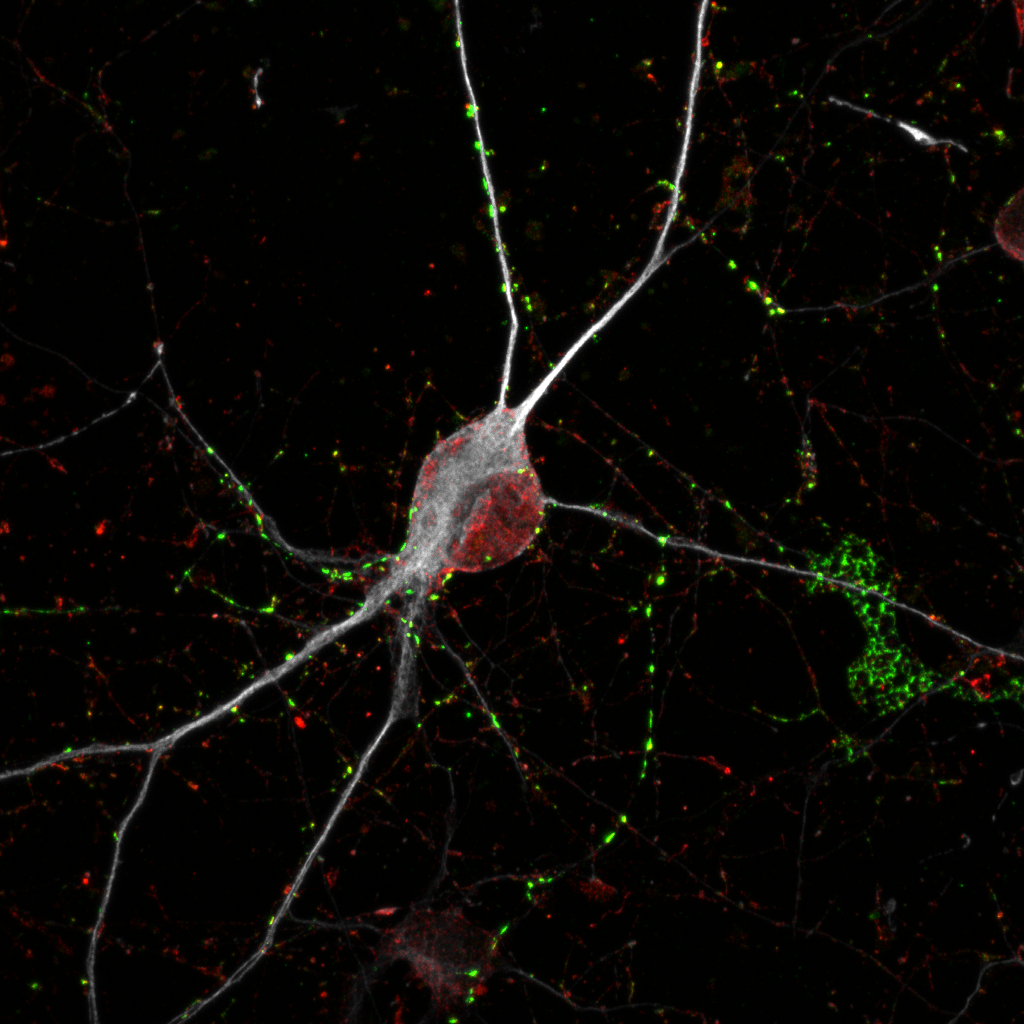

Supplement: Supplementary file 3 — Source data Fig. 1 [file 44321_2024_144_MOESM3_ESM.zip › Figure 1/1E/KO_NPCs/MERGE_KO_NPCs.tif]

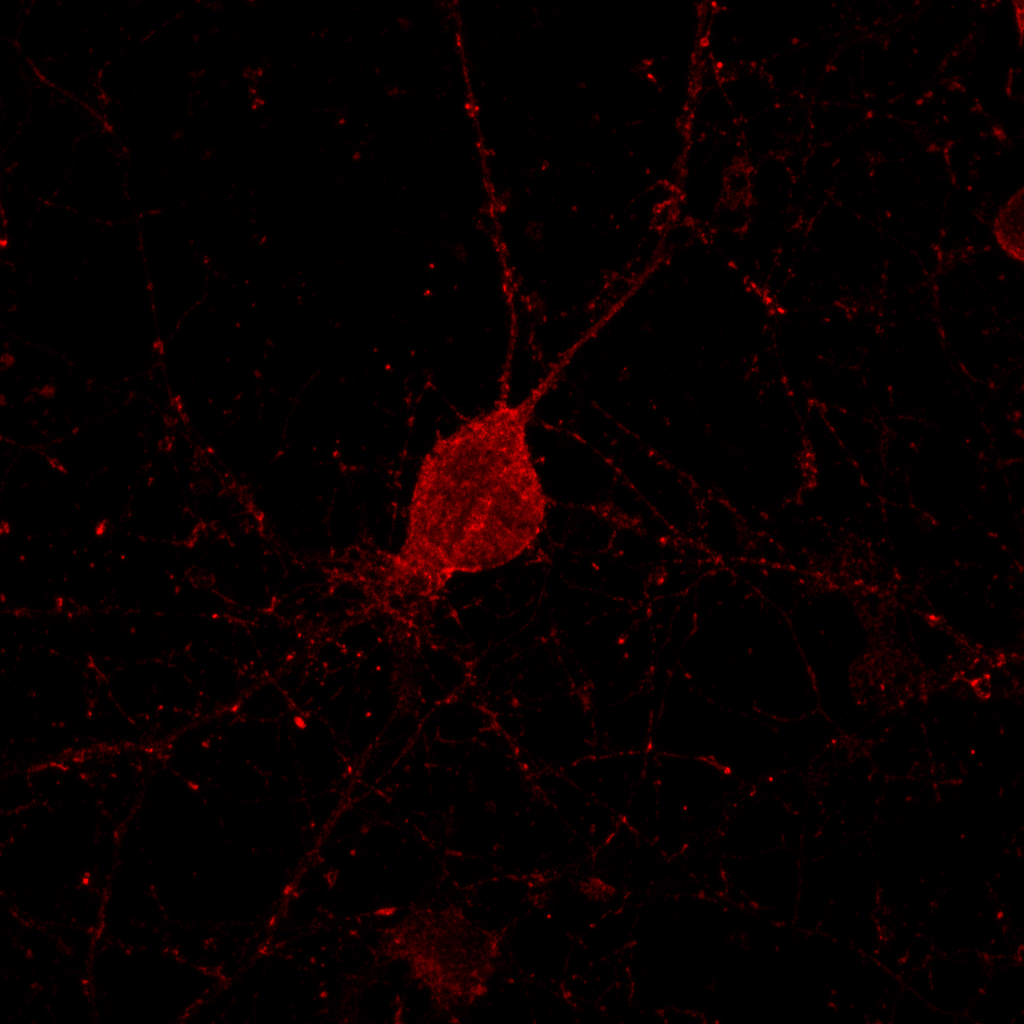

Supplement: Supplementary file 3 — Source data Fig. 1 [file 44321_2024_144_MOESM3_ESM.zip › Figure 1/1E/KO_NPCs/SHANK_KO_NPCs.tif]

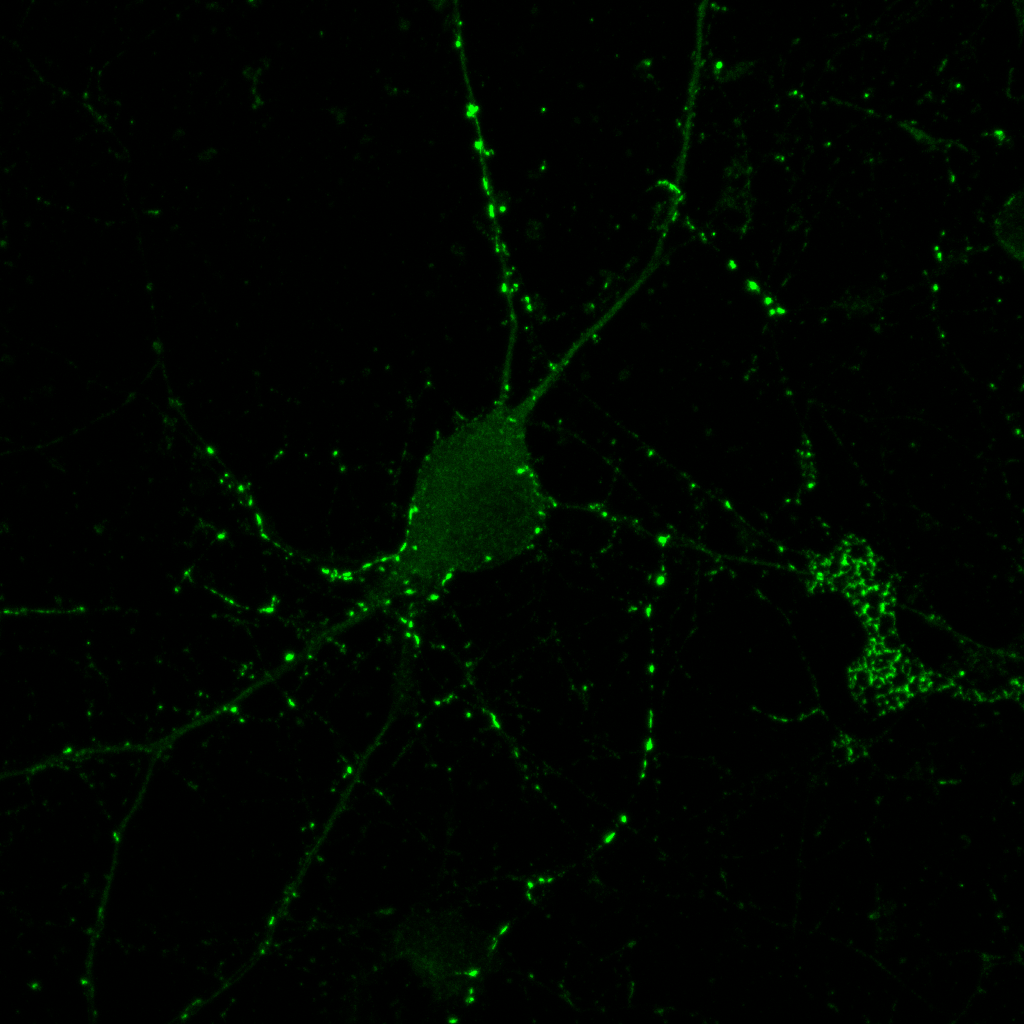

Supplement: Supplementary file 3 — Source data Fig. 1 [file 44321_2024_144_MOESM3_ESM.zip › Figure 1/1E/KO_NPCs/SYN_KO_NPCs.tif]

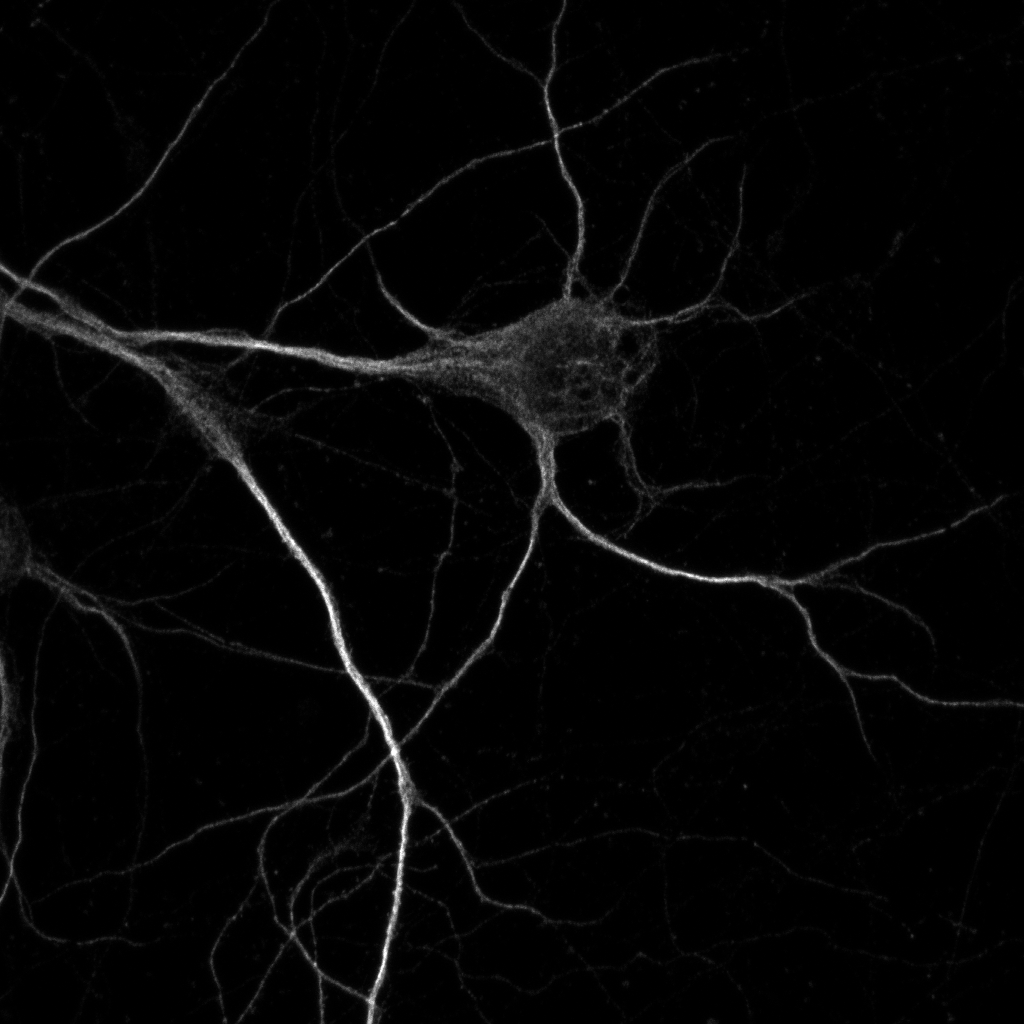

Supplement: Supplementary file 3 — Source data Fig. 1 [file 44321_2024_144_MOESM3_ESM.zip › Figure 1/1E/KO_UT/MAP2_KO_UT.tif]

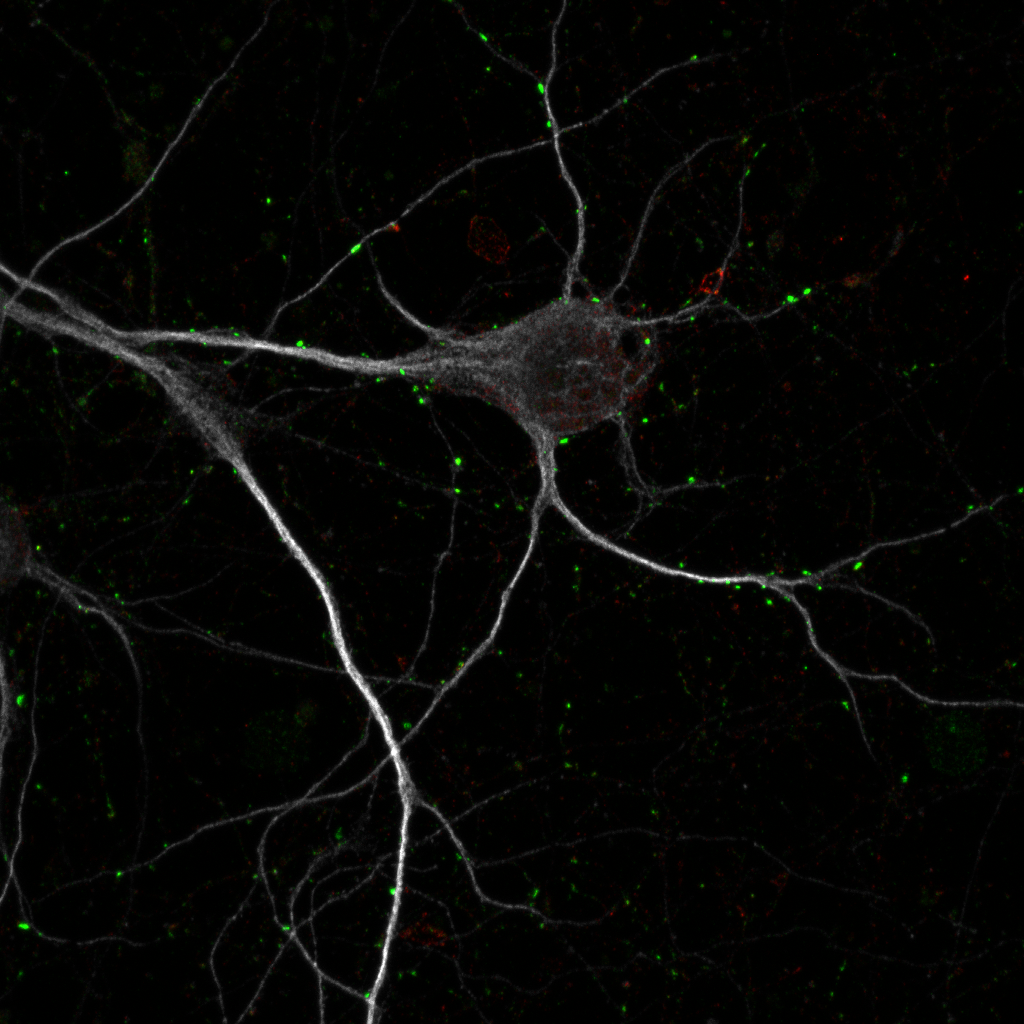

Supplement: Supplementary file 3 — Source data Fig. 1 [file 44321_2024_144_MOESM3_ESM.zip › Figure 1/1E/KO_UT/MERGE_KO_UT.tif]

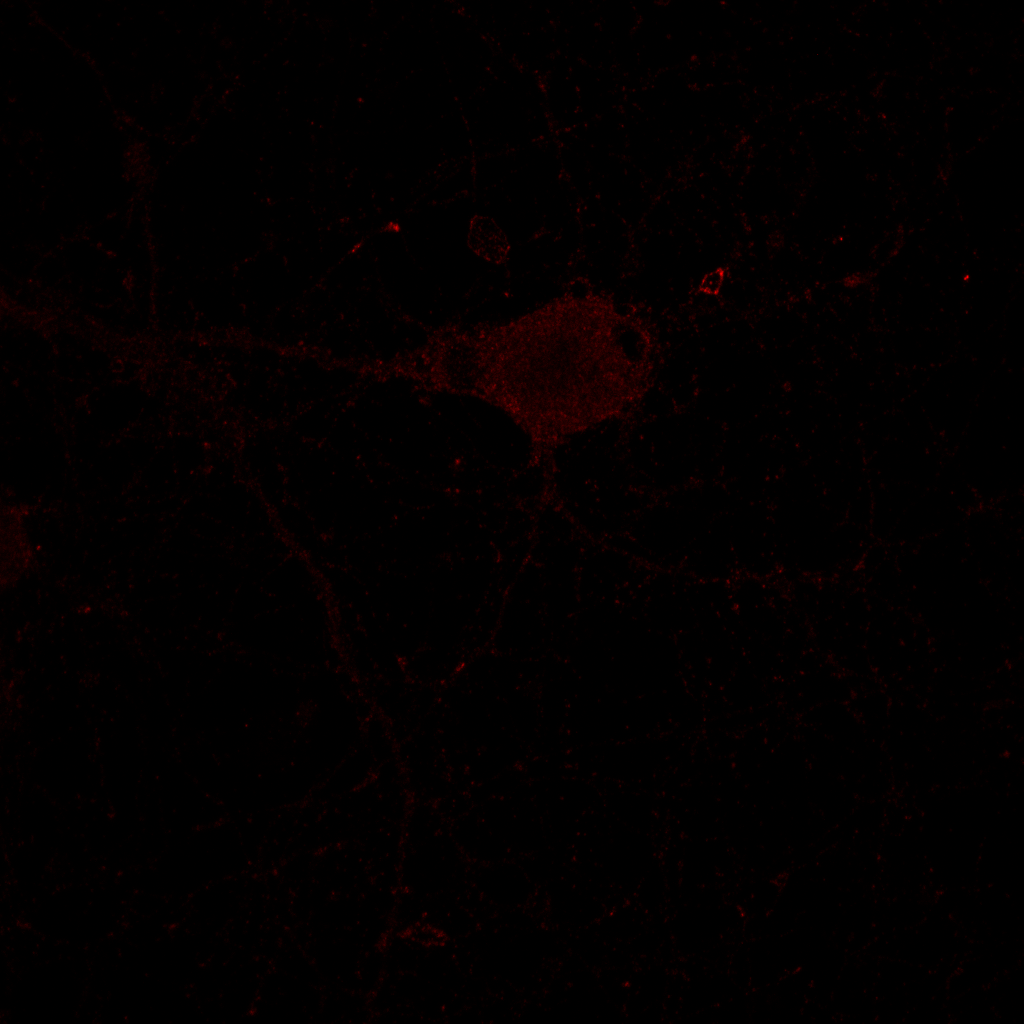

Supplement: Supplementary file 3 — Source data Fig. 1 [file 44321_2024_144_MOESM3_ESM.zip › Figure 1/1E/KO_UT/SHANK_KO_UT.tif]

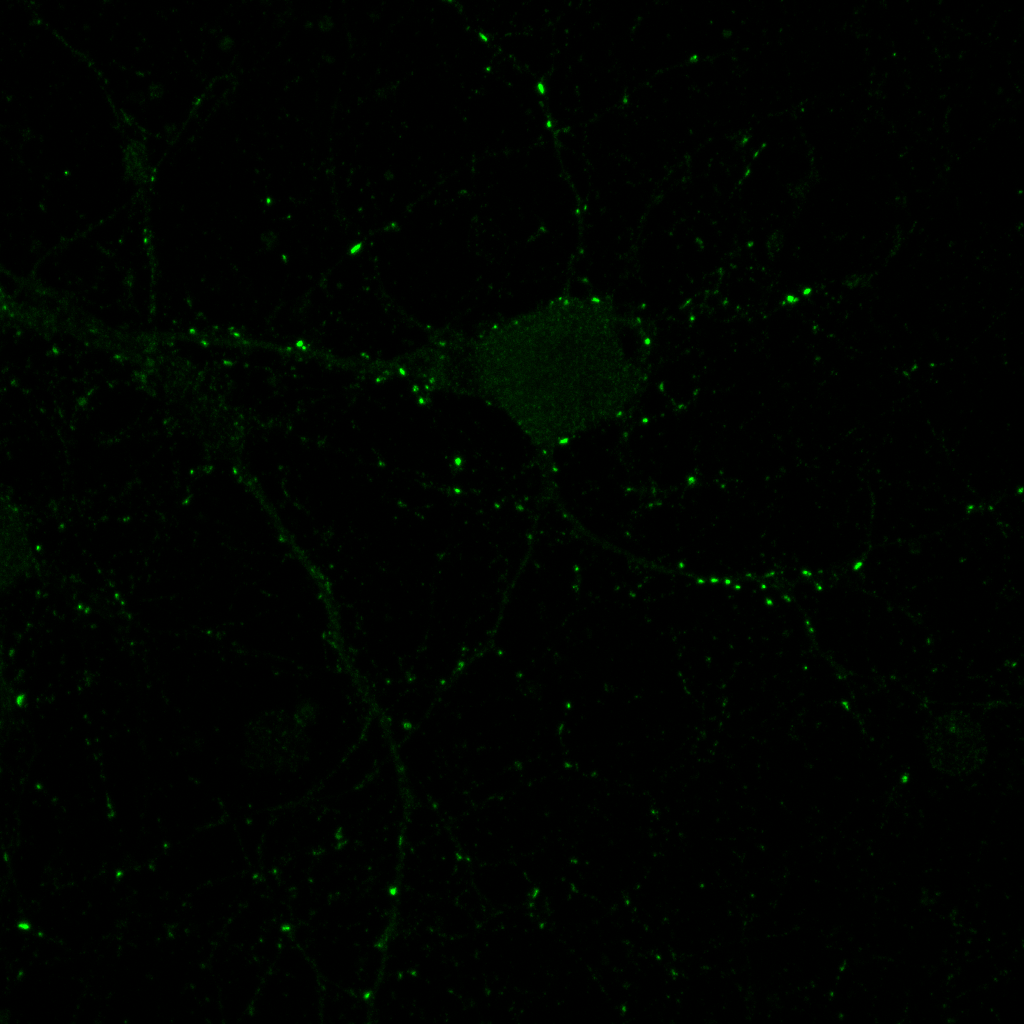

Supplement: Supplementary file 3 — Source data Fig. 1 [file 44321_2024_144_MOESM3_ESM.zip › Figure 1/1E/KO_UT/SYN_KO_UT.tif]

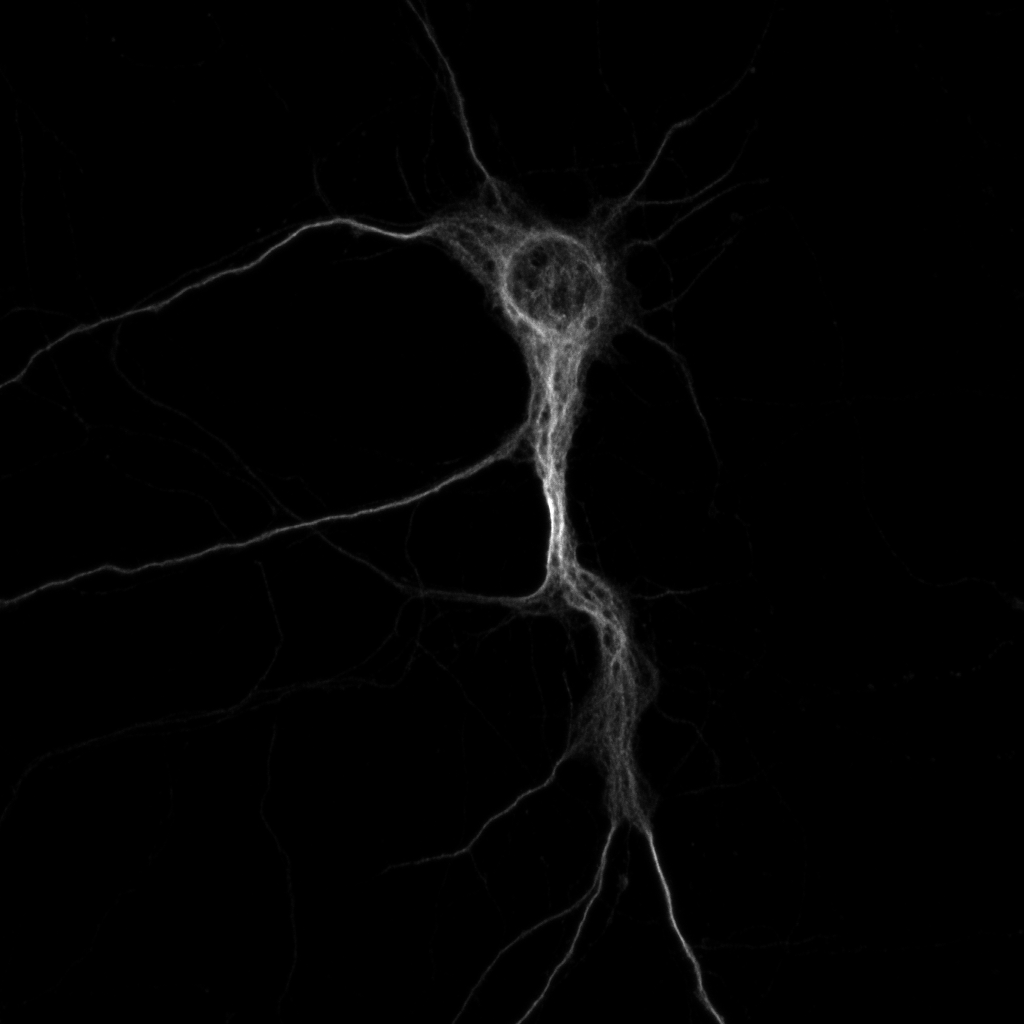

Supplement: Supplementary file 3 — Source data Fig. 1 [file 44321_2024_144_MOESM3_ESM.zip › Figure 1/1E/WT_UT/MAP2_WT_UT.tif]

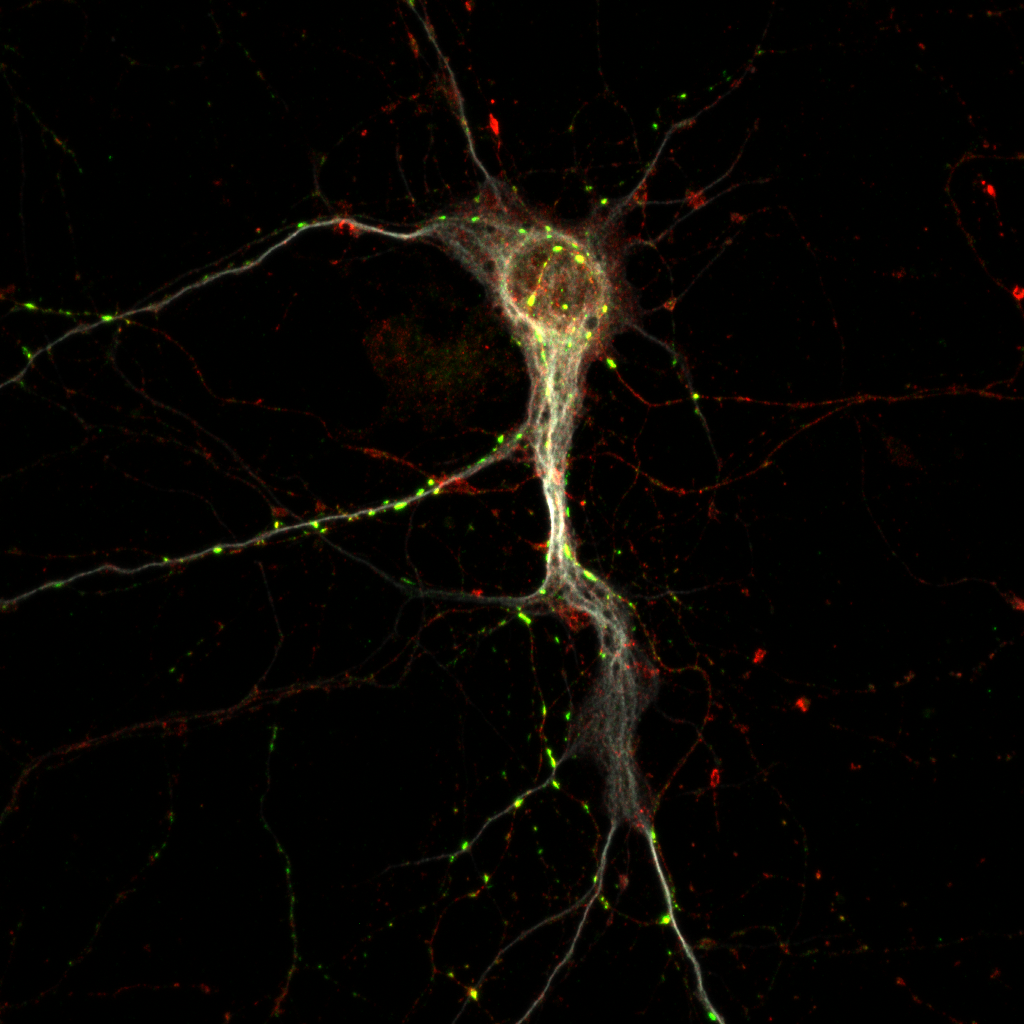

Supplement: Supplementary file 3 — Source data Fig. 1 [file 44321_2024_144_MOESM3_ESM.zip › Figure 1/1E/WT_UT/MERGE_WT_UT.tif]

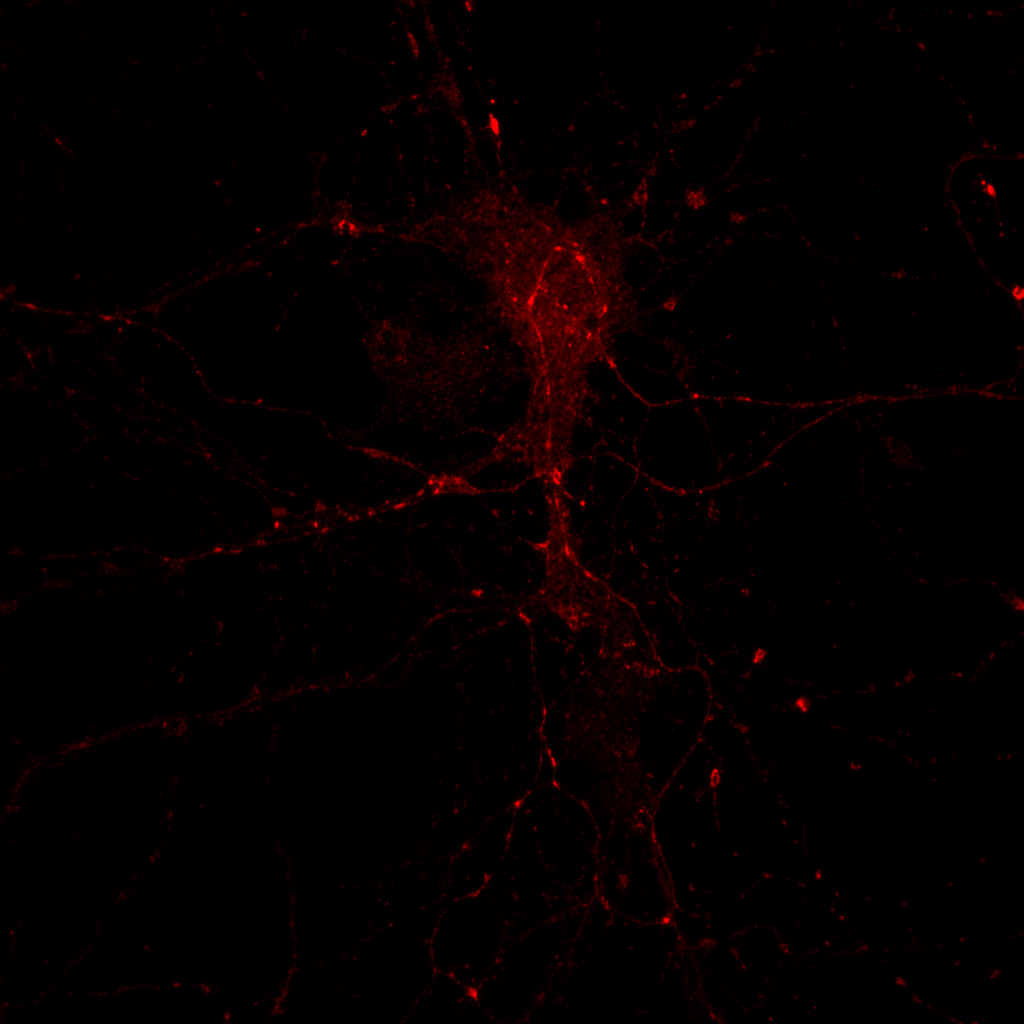

Supplement: Supplementary file 3 — Source data Fig. 1 [file 44321_2024_144_MOESM3_ESM.zip › Figure 1/1E/WT_UT/SHANK_WT_UT.tif]

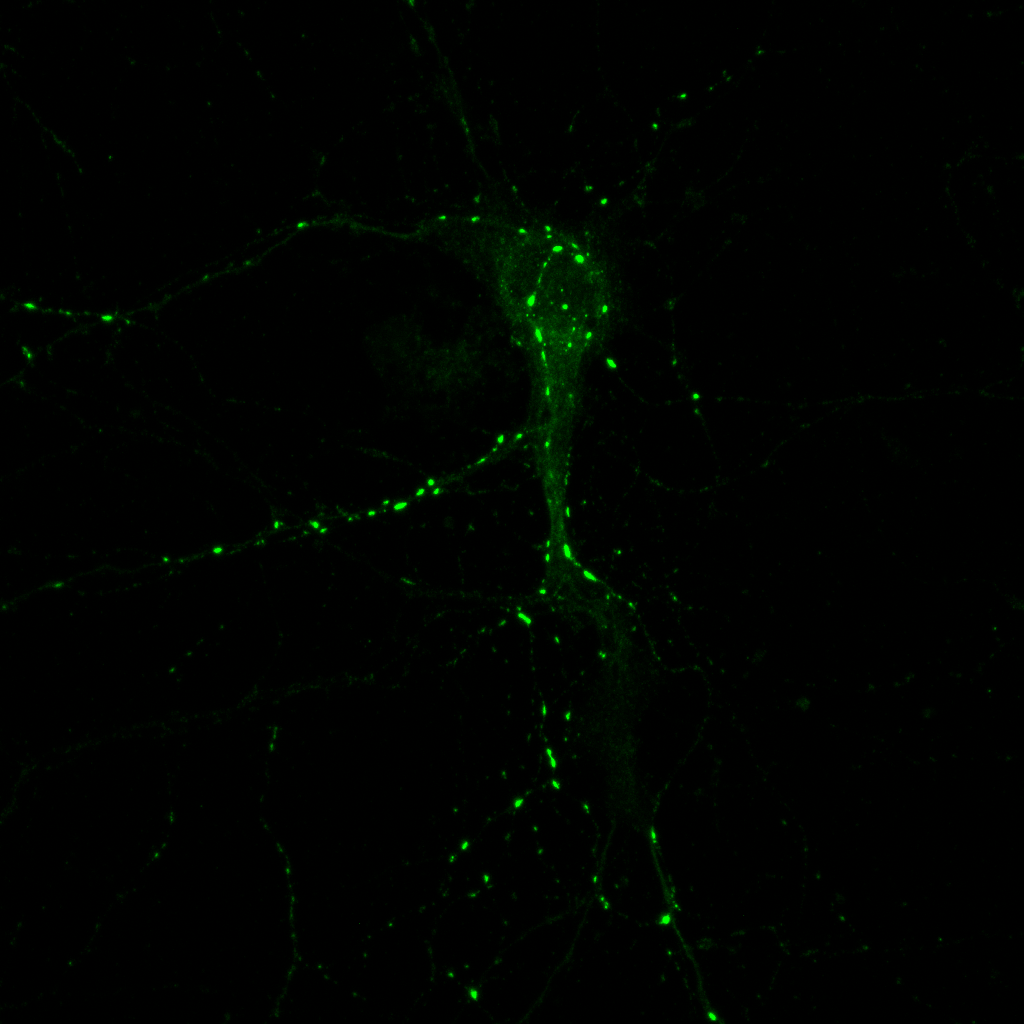

Supplement: Supplementary file 3 — Source data Fig. 1 [file 44321_2024_144_MOESM3_ESM.zip › Figure 1/1E/WT_UT/SYN_WT_UT.tif]

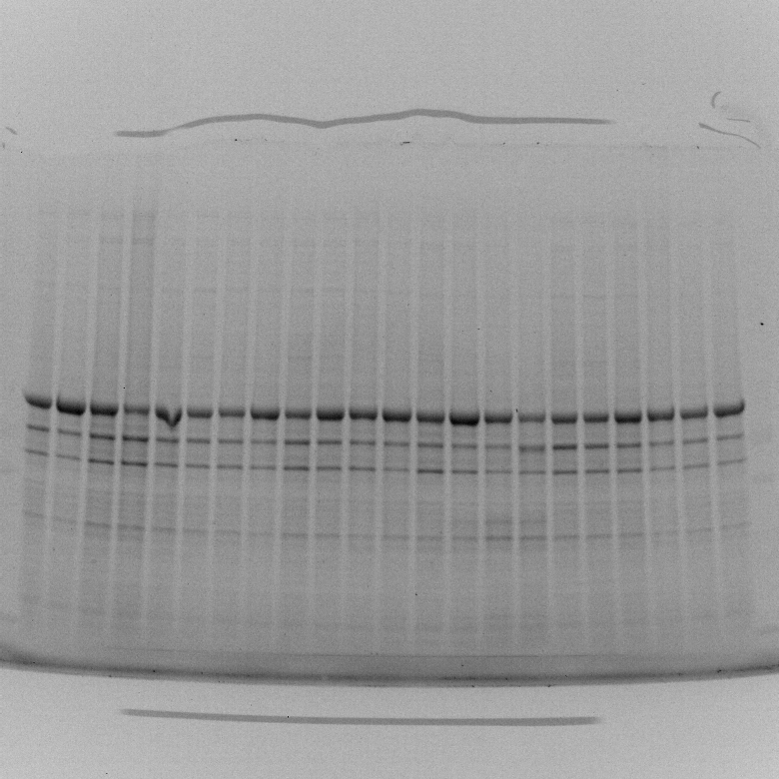

Supplement: Supplementary file 3 — Source data Fig. 1 [file 44321_2024_144_MOESM3_ESM.zip › Figure 1/1I/1I_TGX-stain free gel.tif]

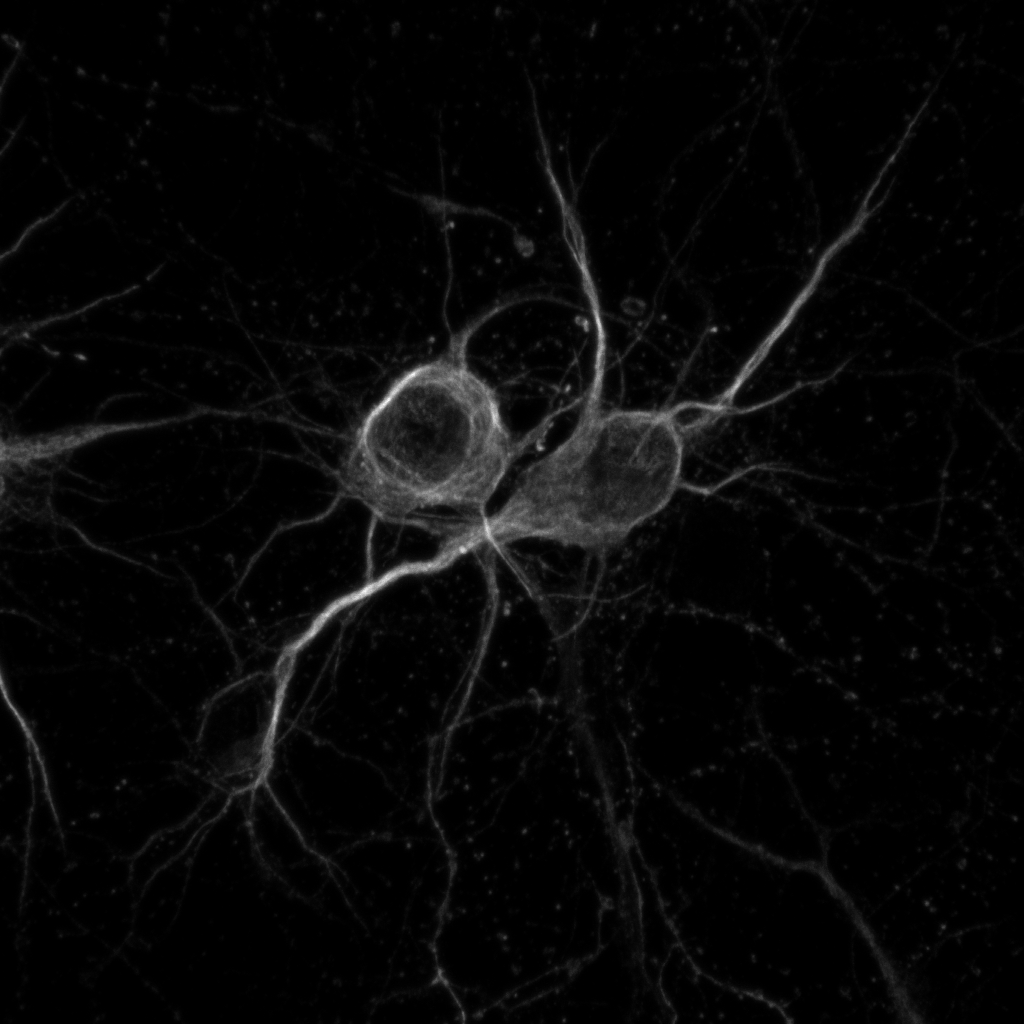

Supplement: Supplementary file 3 — Source data Fig. 1 [file 44321_2024_144_MOESM3_ESM.zip › Figure 1/1J/Mecp2 HET/MAP2_Mecp2_HET.tif]

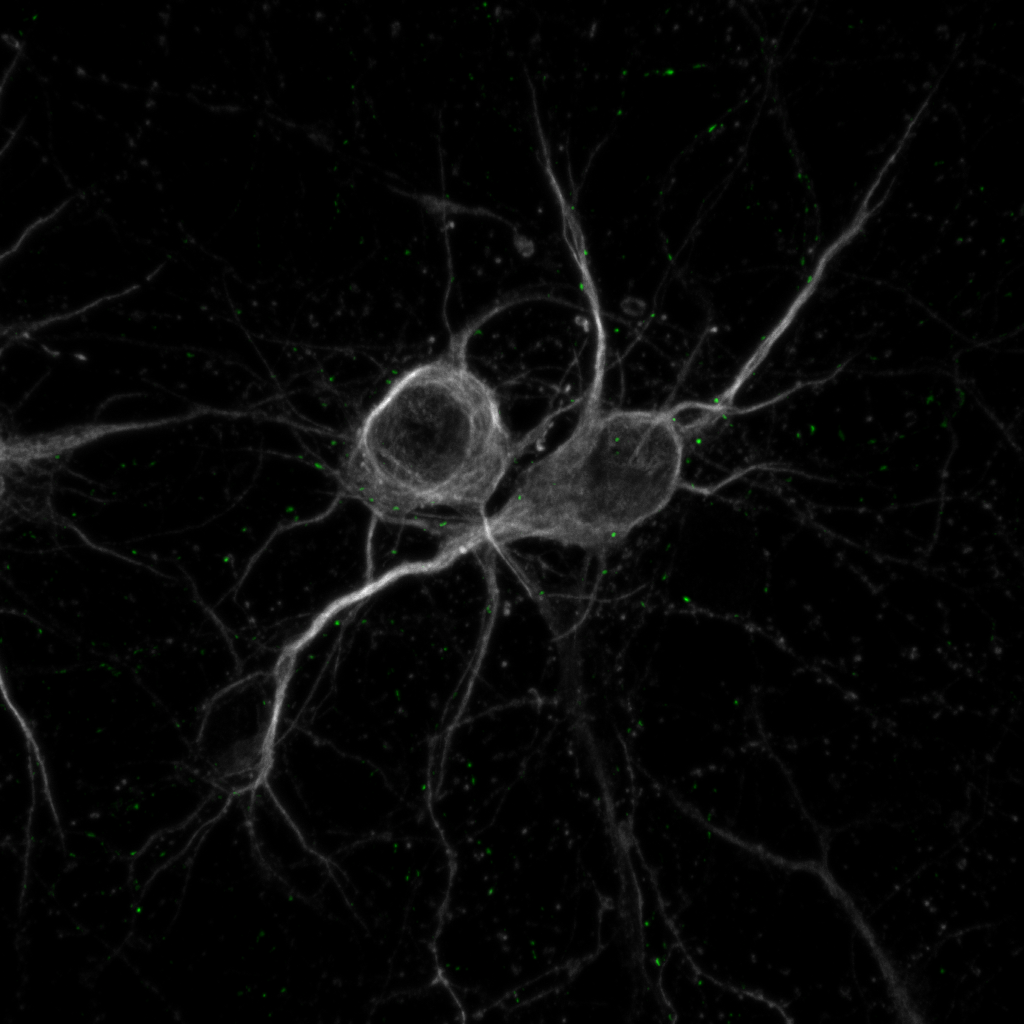

Supplement: Supplementary file 3 — Source data Fig. 1 [file 44321_2024_144_MOESM3_ESM.zip › Figure 1/1J/Mecp2 HET/MERGE_Mecp2_HET.tif]

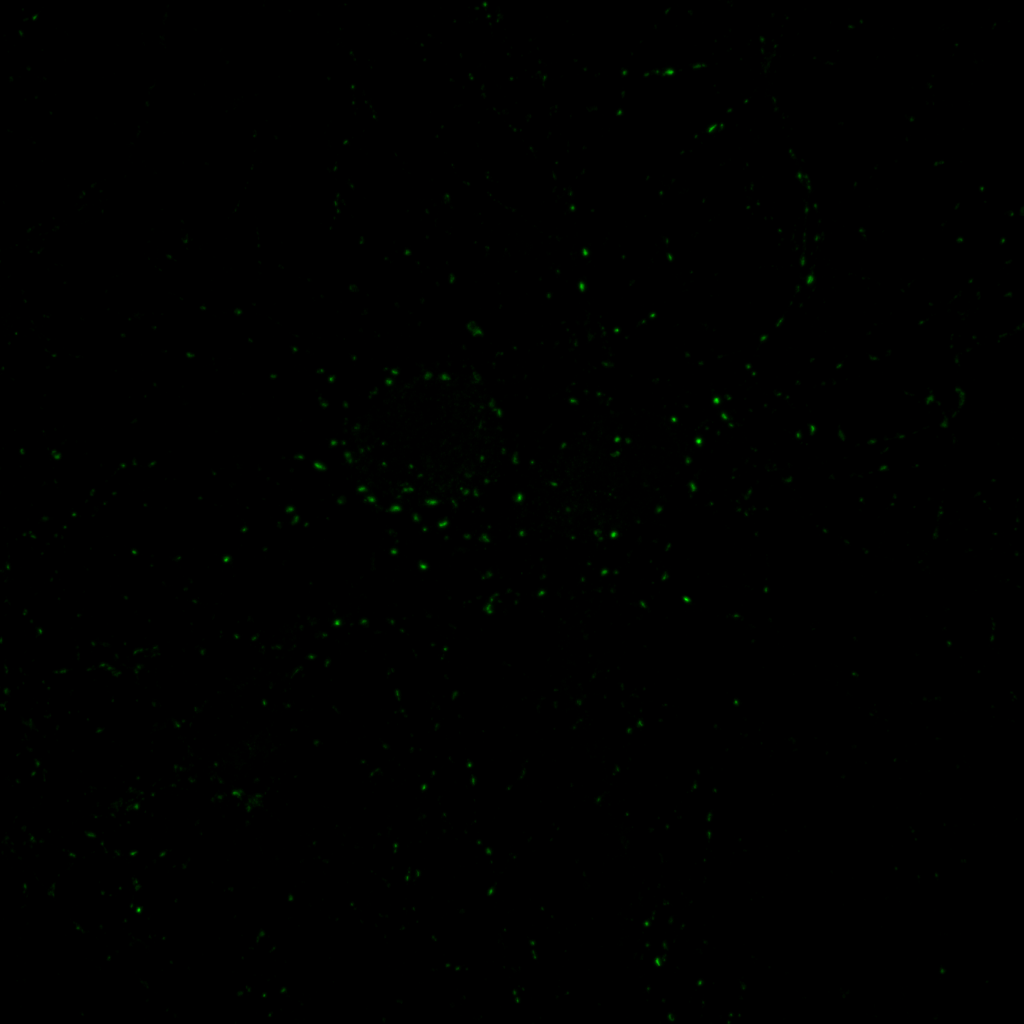

Supplement: Supplementary file 3 — Source data Fig. 1 [file 44321_2024_144_MOESM3_ESM.zip › Figure 1/1J/Mecp2 HET/SYN_Mecp2_HET.tif]

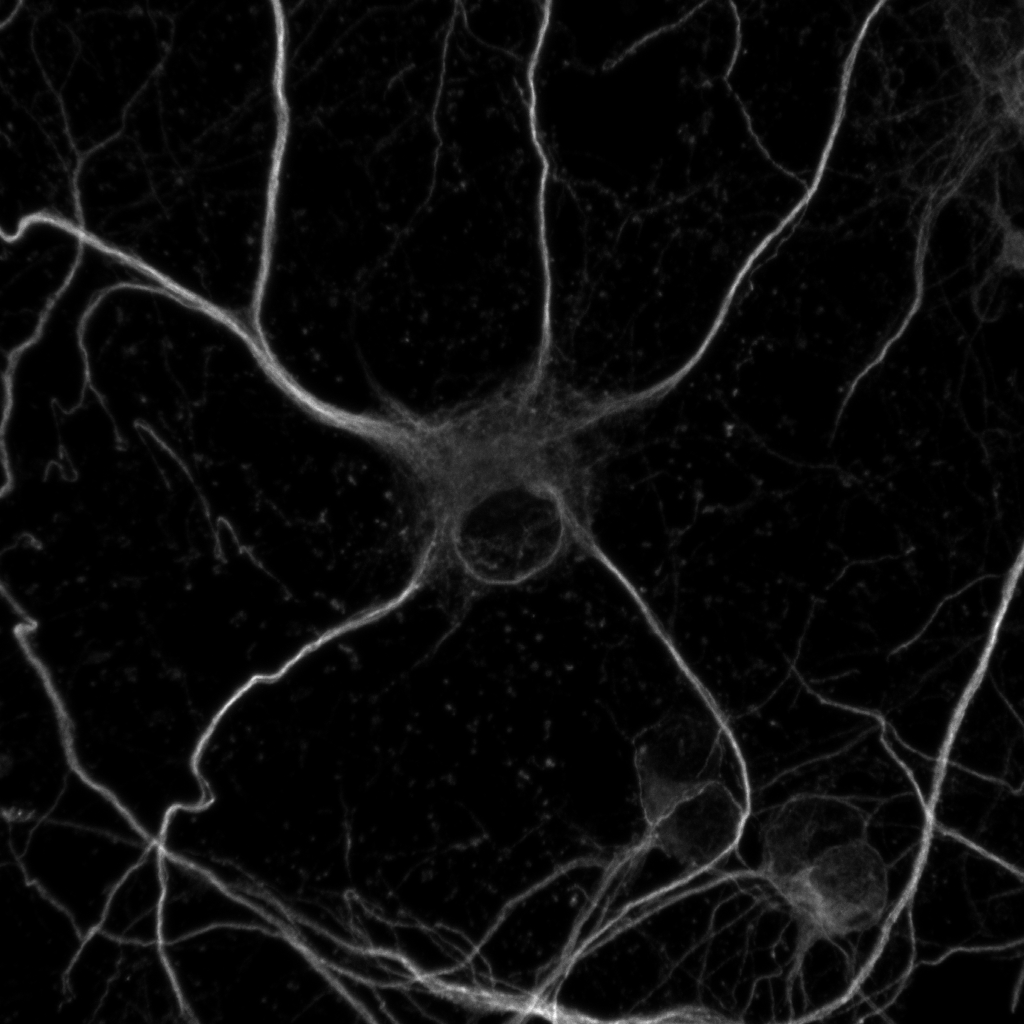

Supplement: Supplementary file 3 — Source data Fig. 1 [file 44321_2024_144_MOESM3_ESM.zip › Figure 1/1J/Mecp2 HET+NPCs/MAP2_Mecp2_HET+NPCs.tif]

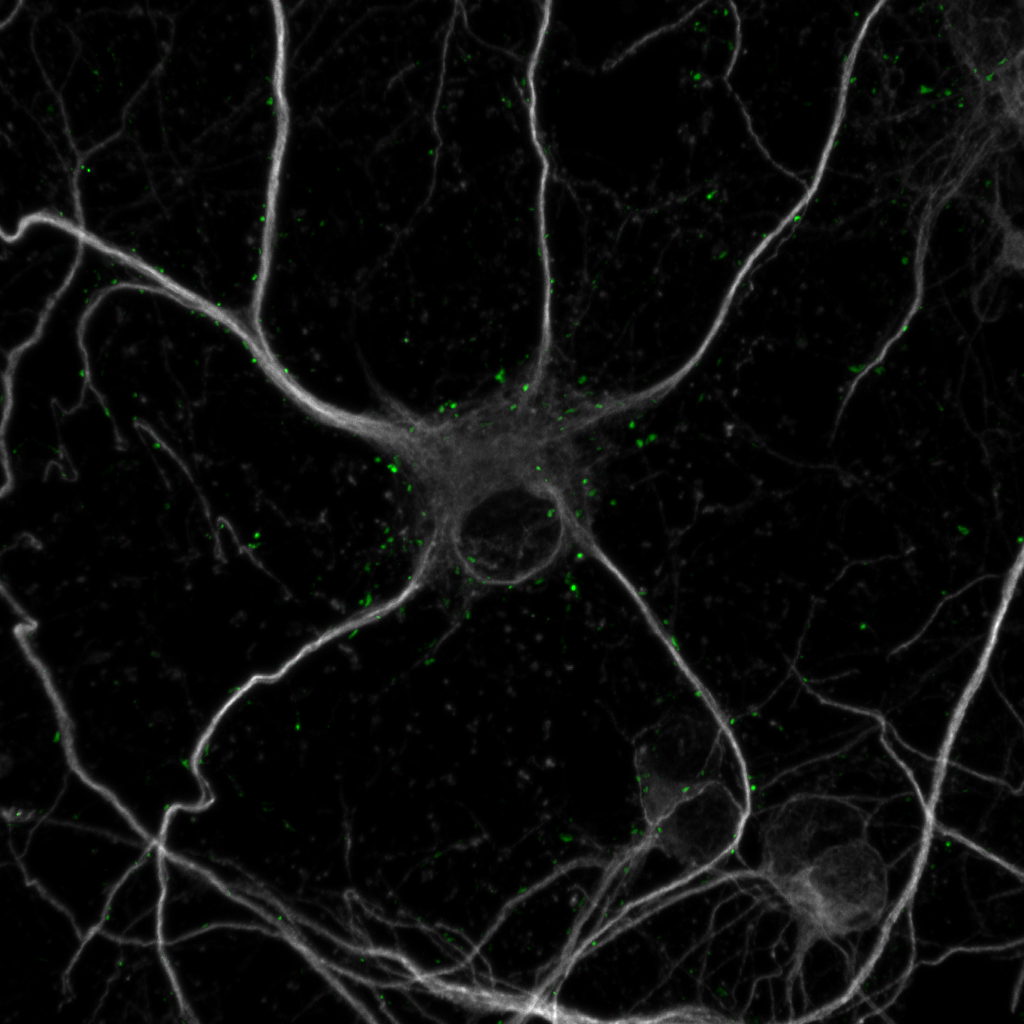

Supplement: Supplementary file 3 — Source data Fig. 1 [file 44321_2024_144_MOESM3_ESM.zip › Figure 1/1J/Mecp2 HET+NPCs/MERGE_Mecp2_HET+NPCs.tif]

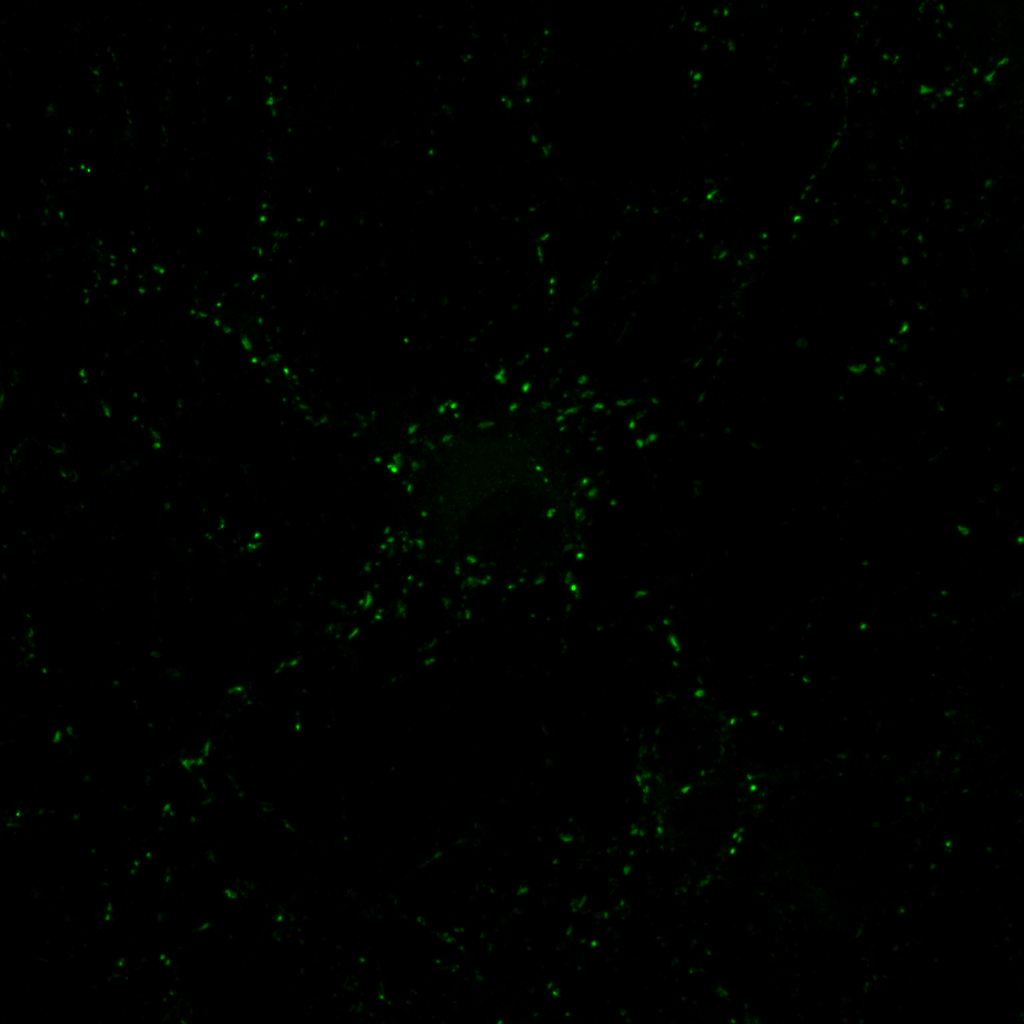

Supplement: Supplementary file 3 — Source data Fig. 1 [file 44321_2024_144_MOESM3_ESM.zip › Figure 1/1J/Mecp2 HET+NPCs/SYN_Mecp2_HET+NPCs.tif]

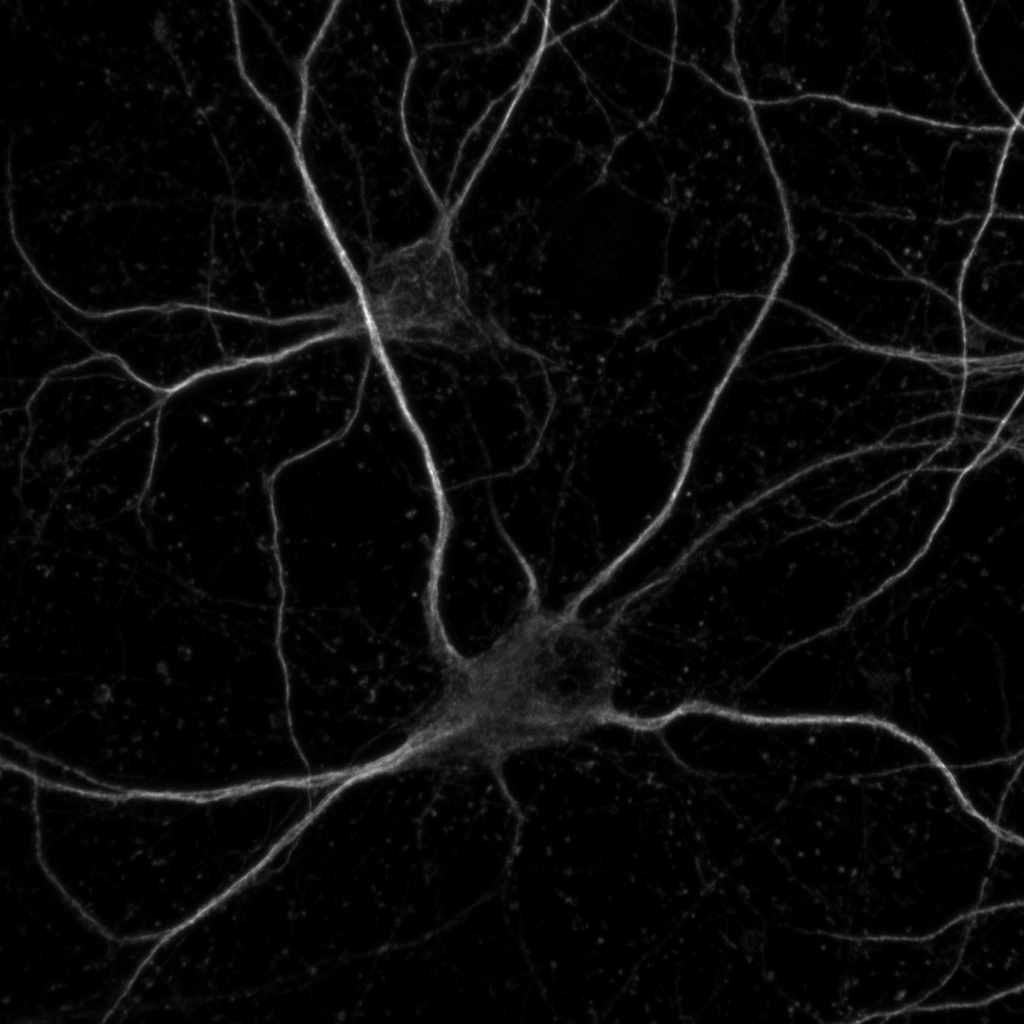

Supplement: Supplementary file 3 — Source data Fig. 1 [file 44321_2024_144_MOESM3_ESM.zip › Figure 1/1J/WT/MAP2_WT.tif]

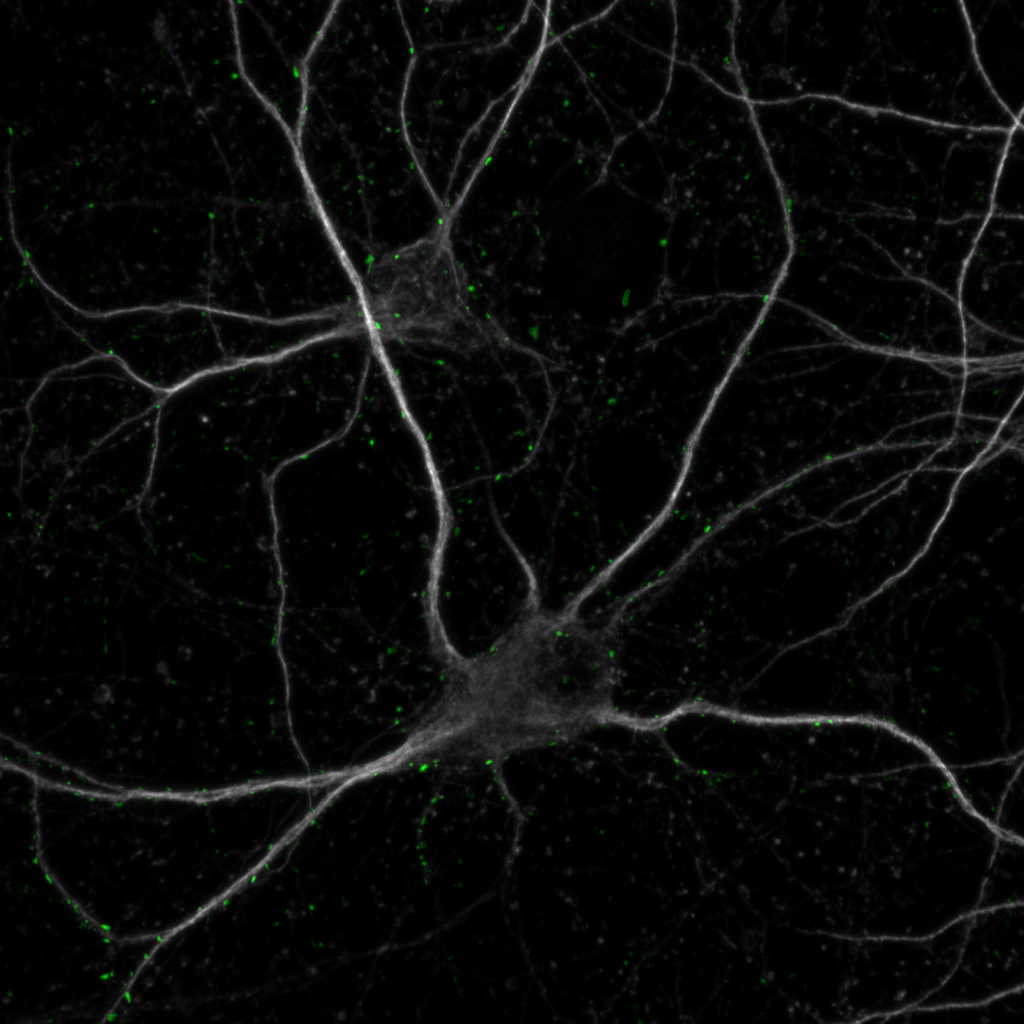

Supplement: Supplementary file 3 — Source data Fig. 1 [file 44321_2024_144_MOESM3_ESM.zip › Figure 1/1J/WT/MERGE_WT.tif]

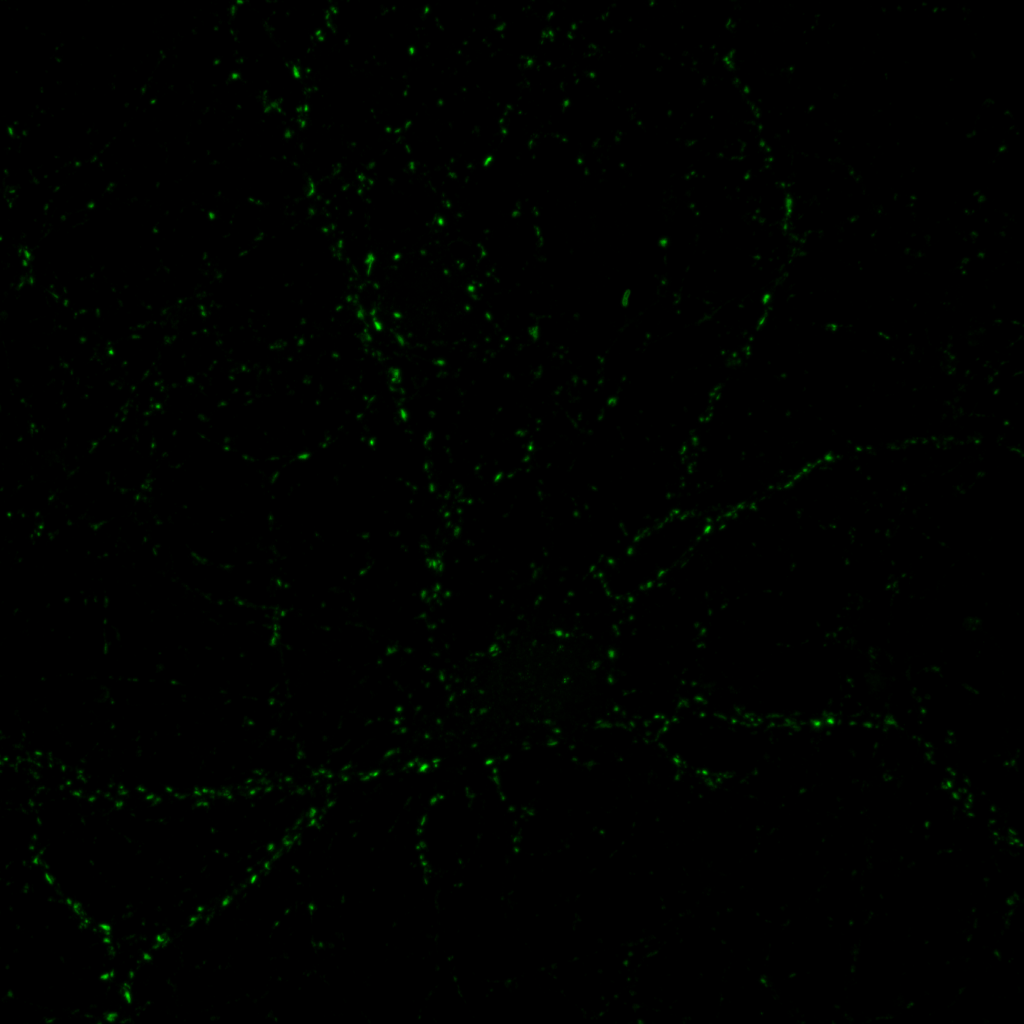

Supplement: Supplementary file 3 — Source data Fig. 1 [file 44321_2024_144_MOESM3_ESM.zip › Figure 1/1J/WT/SYN_WT.tif]

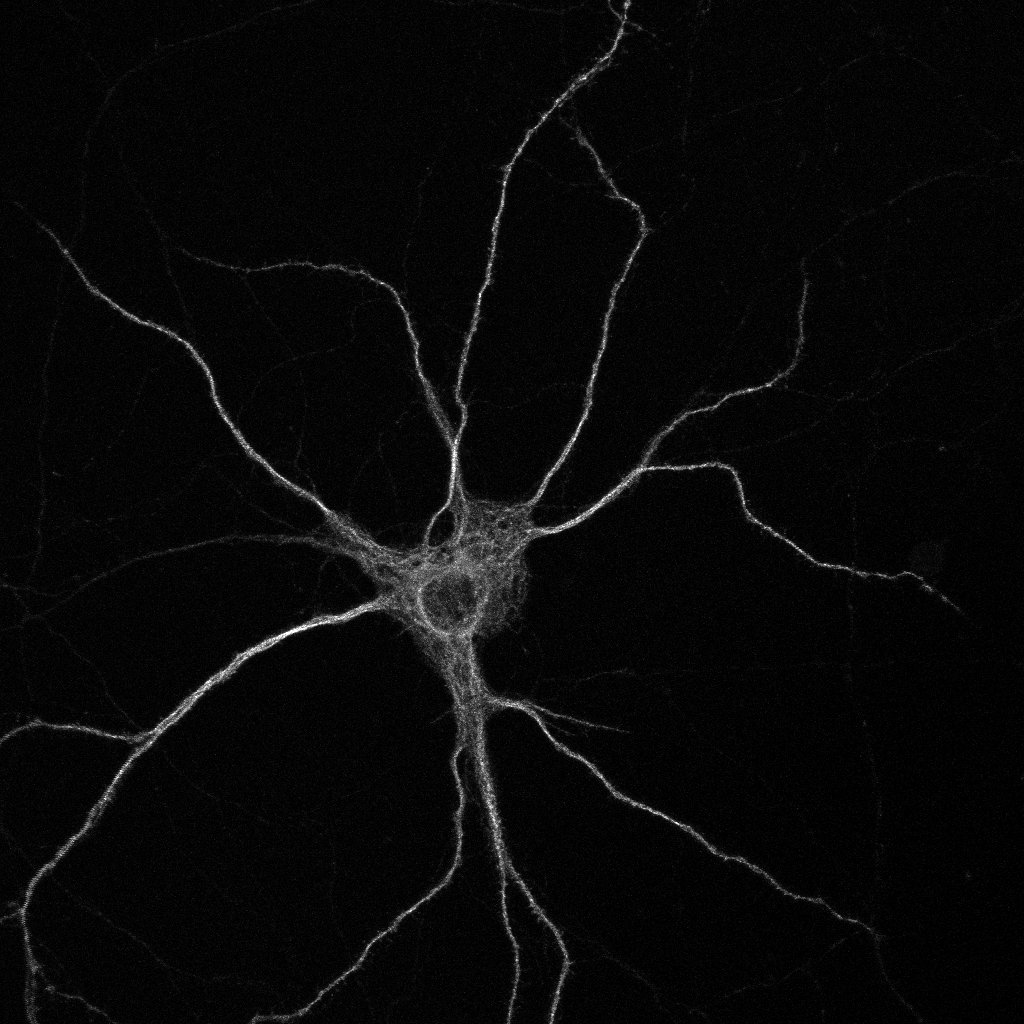

Supplement: Supplementary file 4 — Source data Fig. 2 [file 44321_2024_144_MOESM4_ESM.zip › Figure 2/2B/KO_NPCs/MAP2_KO_NPCs.tif]

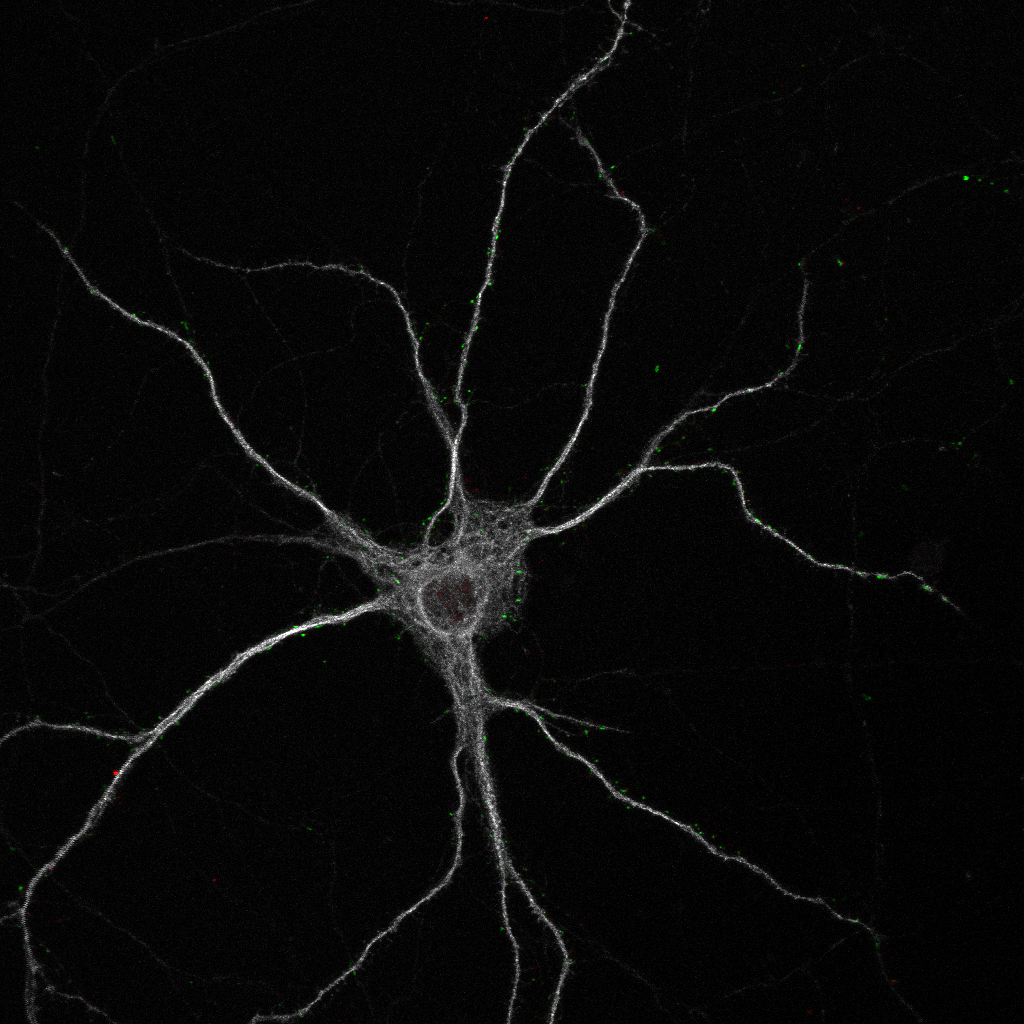

Supplement: Supplementary file 4 — Source data Fig. 2 [file 44321_2024_144_MOESM4_ESM.zip › Figure 2/2B/KO_NPCs/MERGE_KO_NPCs.tif]

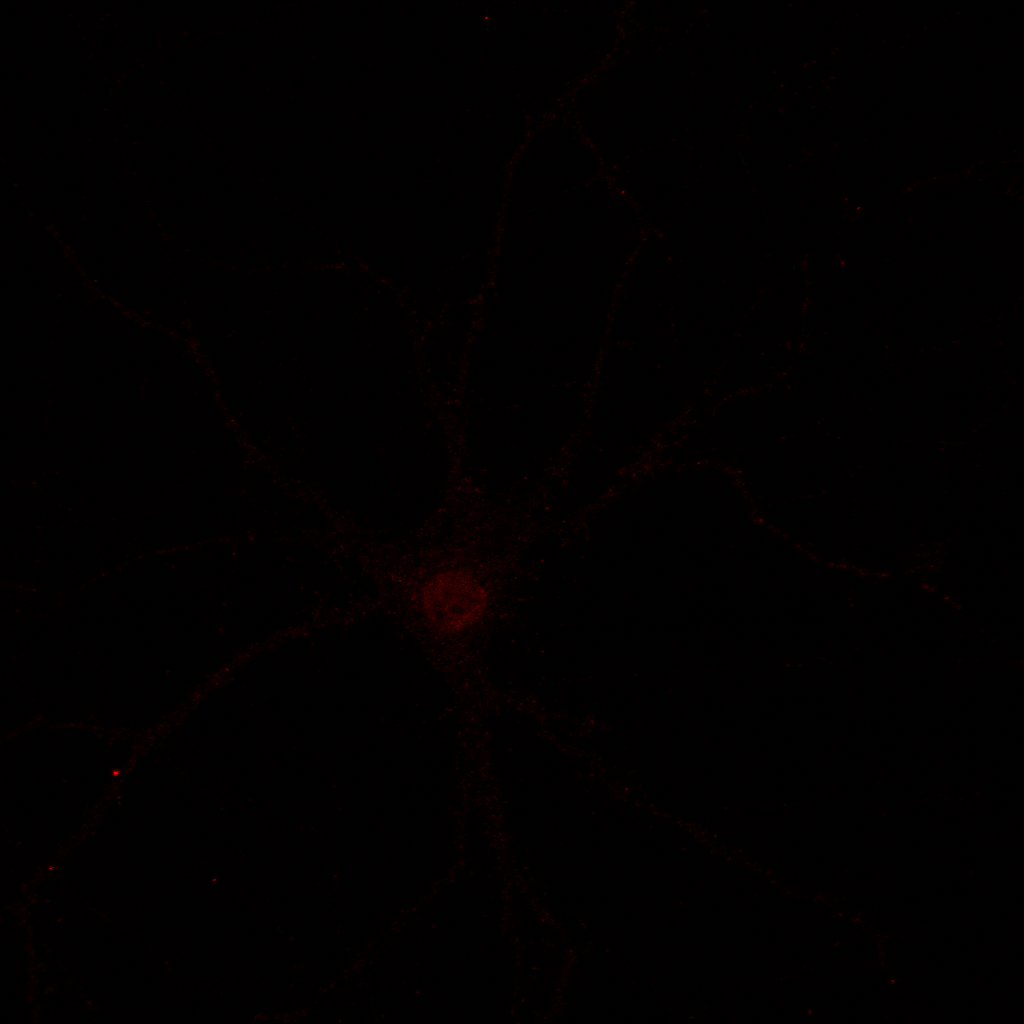

Supplement: Supplementary file 4 — Source data Fig. 2 [file 44321_2024_144_MOESM4_ESM.zip › Figure 2/2B/KO_NPCs/SHANK_KO_NPCs.tif]

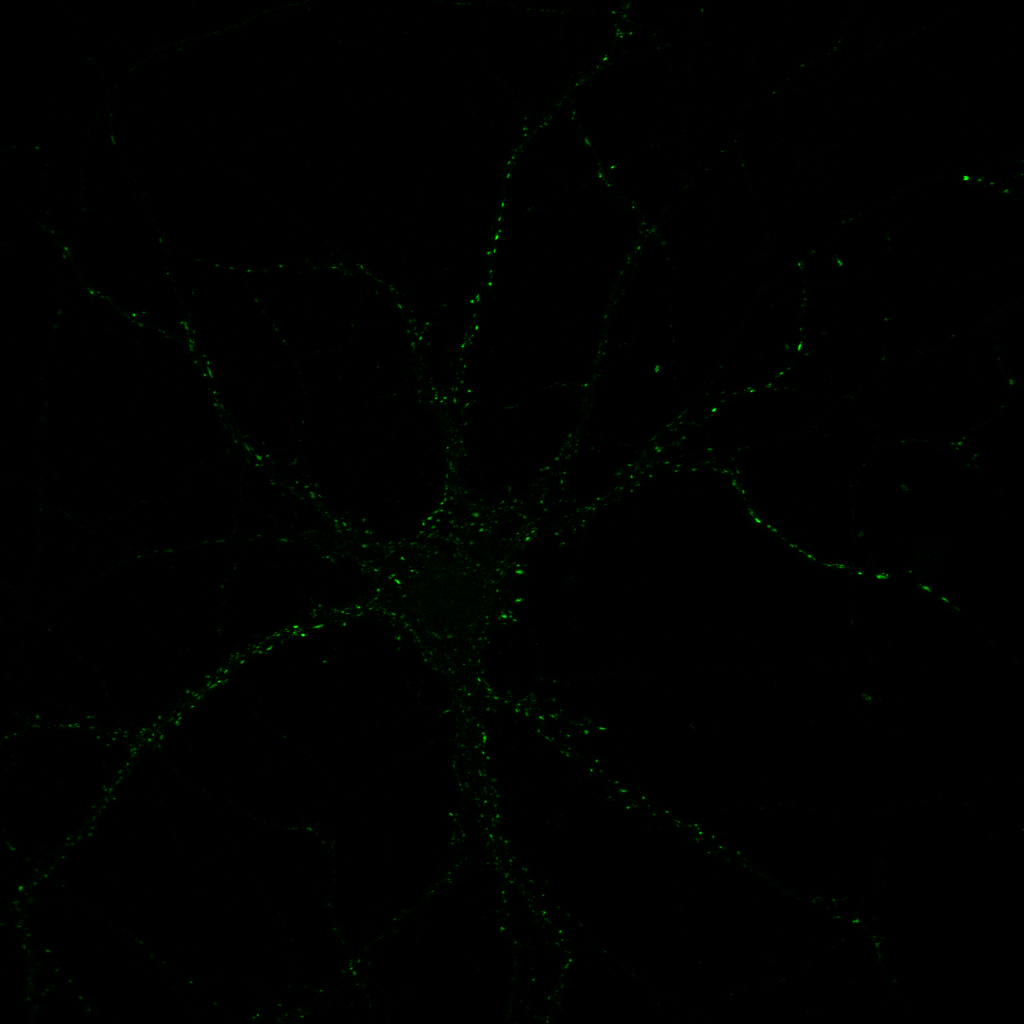

Supplement: Supplementary file 4 — Source data Fig. 2 [file 44321_2024_144_MOESM4_ESM.zip › Figure 2/2B/KO_NPCs/SYN_KO_NPCs.tif]

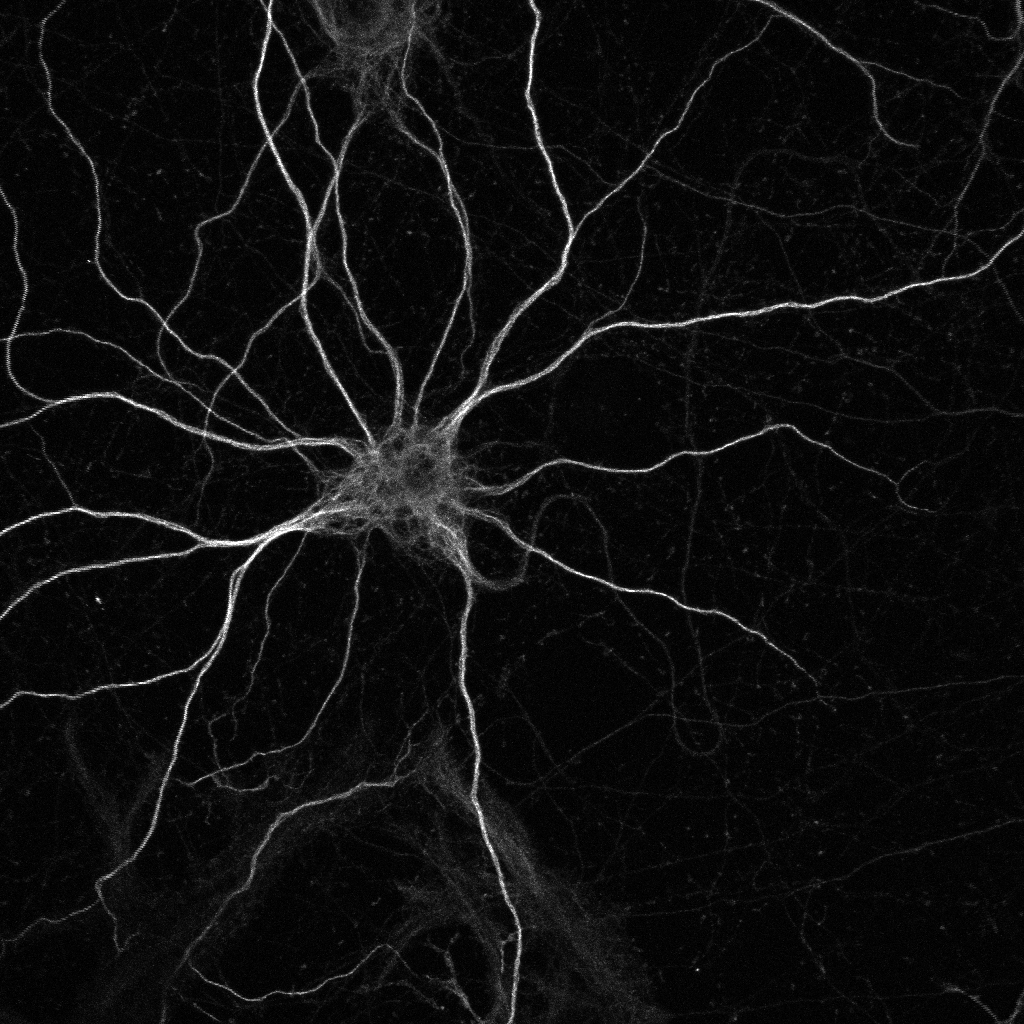

Supplement: Supplementary file 4 — Source data Fig. 2 [file 44321_2024_144_MOESM4_ESM.zip › Figure 2/2B/KO_NPCsKO/MAP2_KO_NPCsKO.tif]

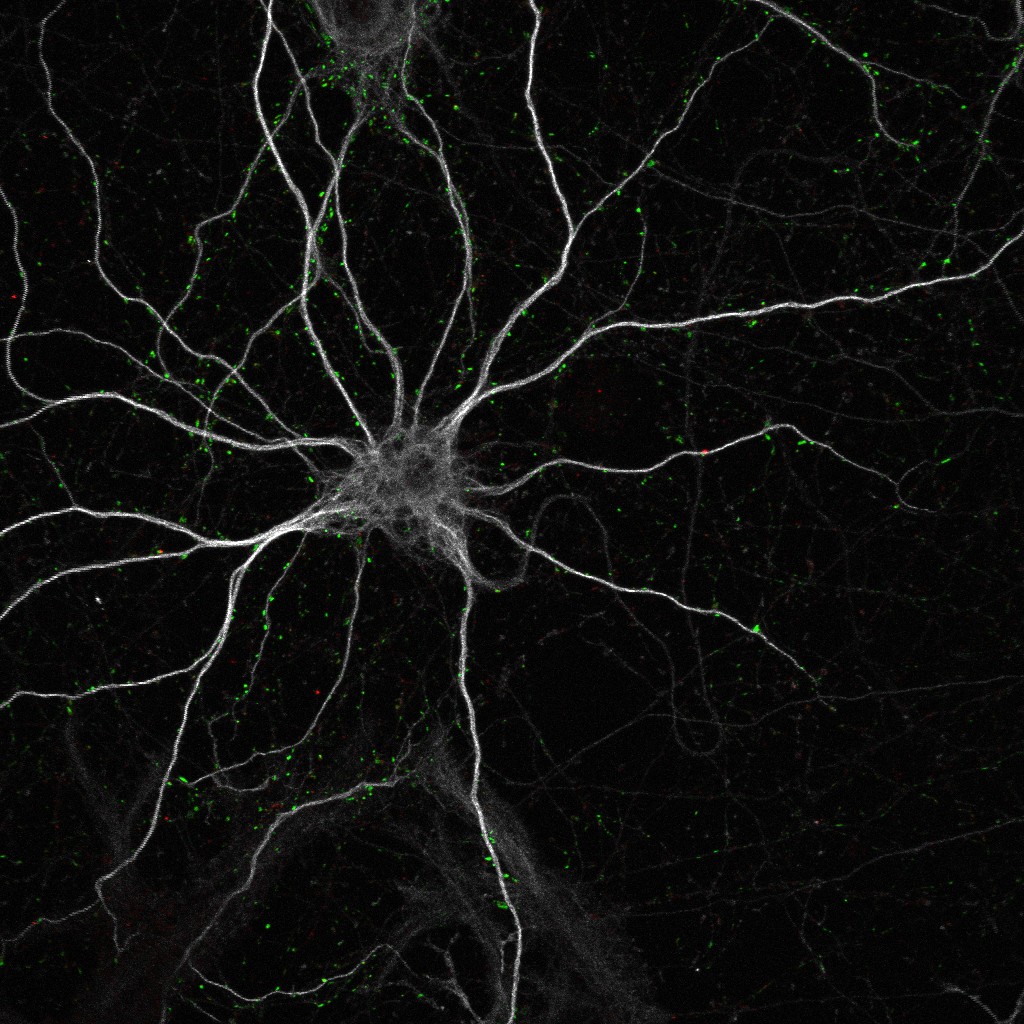

Supplement: Supplementary file 4 — Source data Fig. 2 [file 44321_2024_144_MOESM4_ESM.zip › Figure 2/2B/KO_NPCsKO/MERGE_KO_NPCsKO.tif]

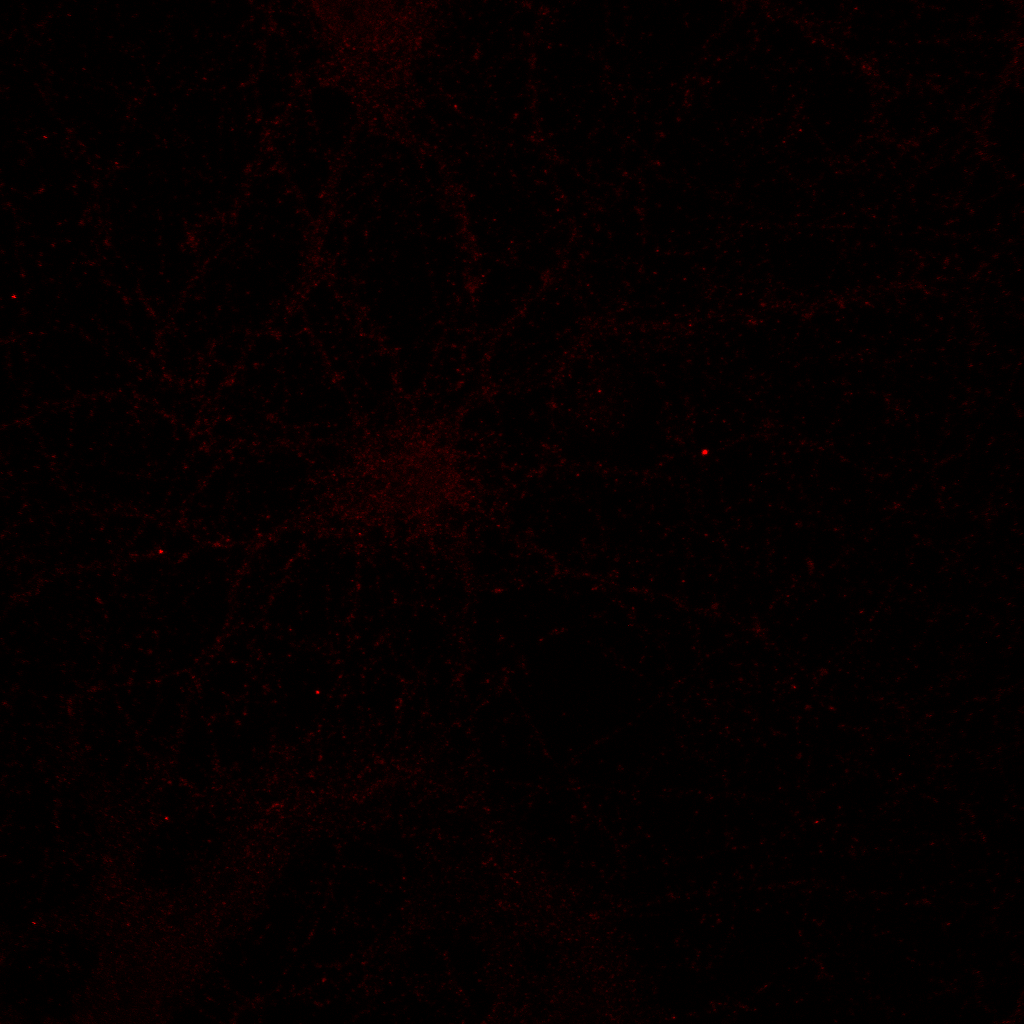

Supplement: Supplementary file 4 — Source data Fig. 2 [file 44321_2024_144_MOESM4_ESM.zip › Figure 2/2B/KO_NPCsKO/SHANK_KO_NPCsKO.tif]

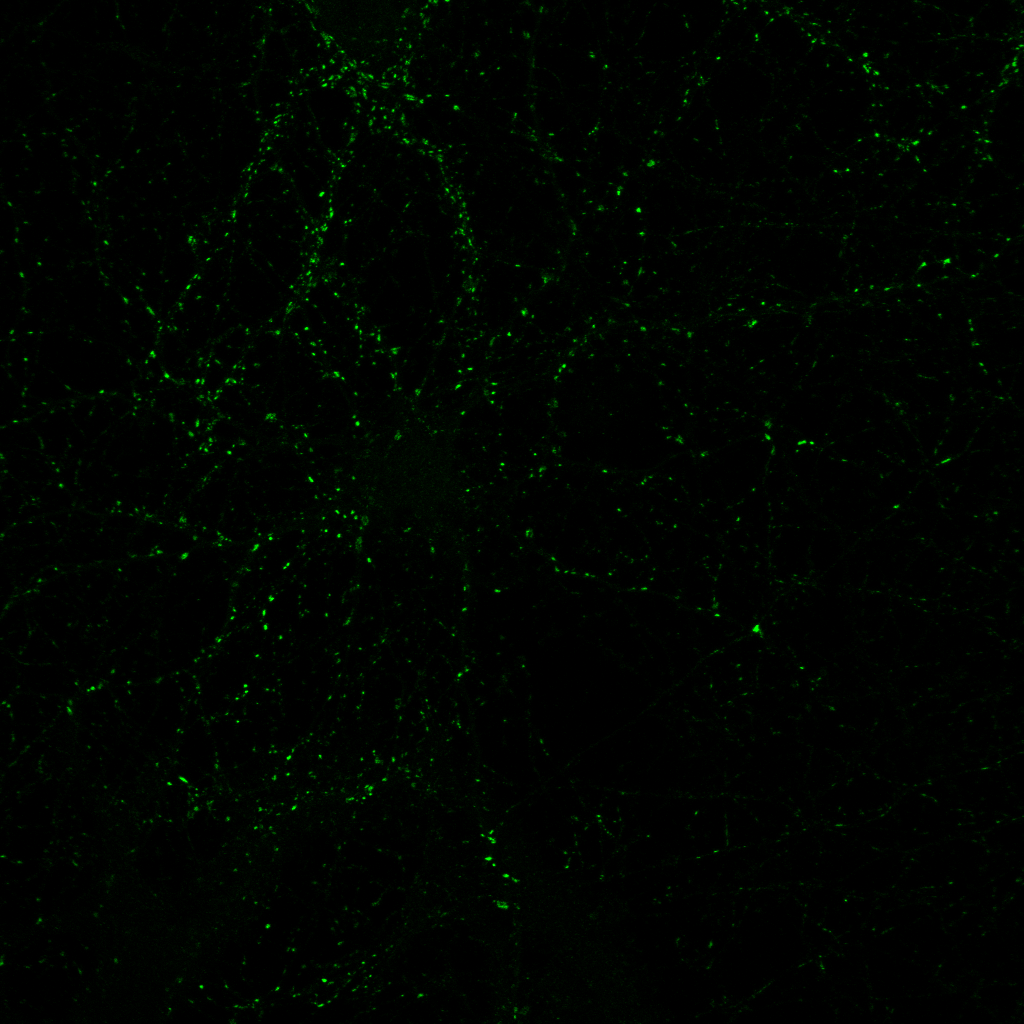

Supplement: Supplementary file 4 — Source data Fig. 2 [file 44321_2024_144_MOESM4_ESM.zip › Figure 2/2B/KO_NPCsKO/SYN_KO_NPCsKO.tif]

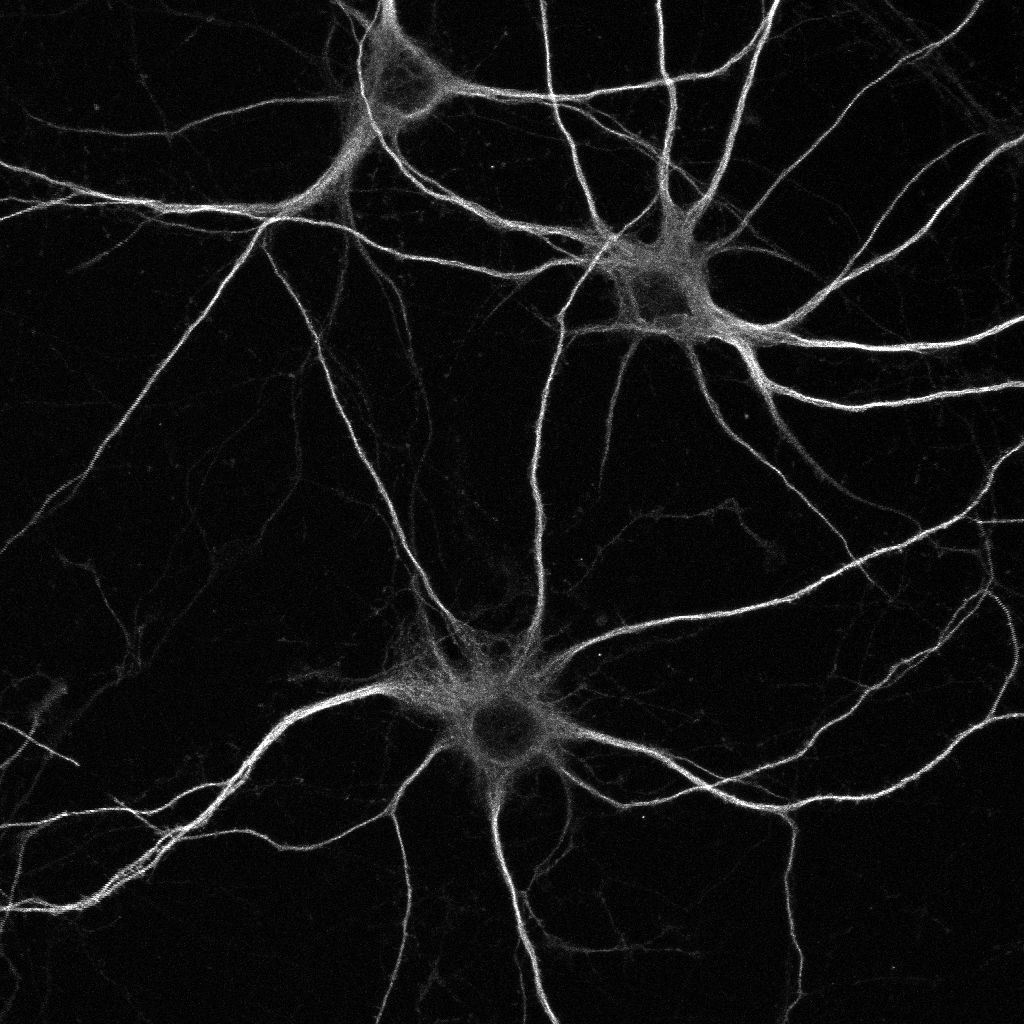

Supplement: Supplementary file 4 — Source data Fig. 2 [file 44321_2024_144_MOESM4_ESM.zip › Figure 2/2B/KO_UT/MAP2_UT.tif]

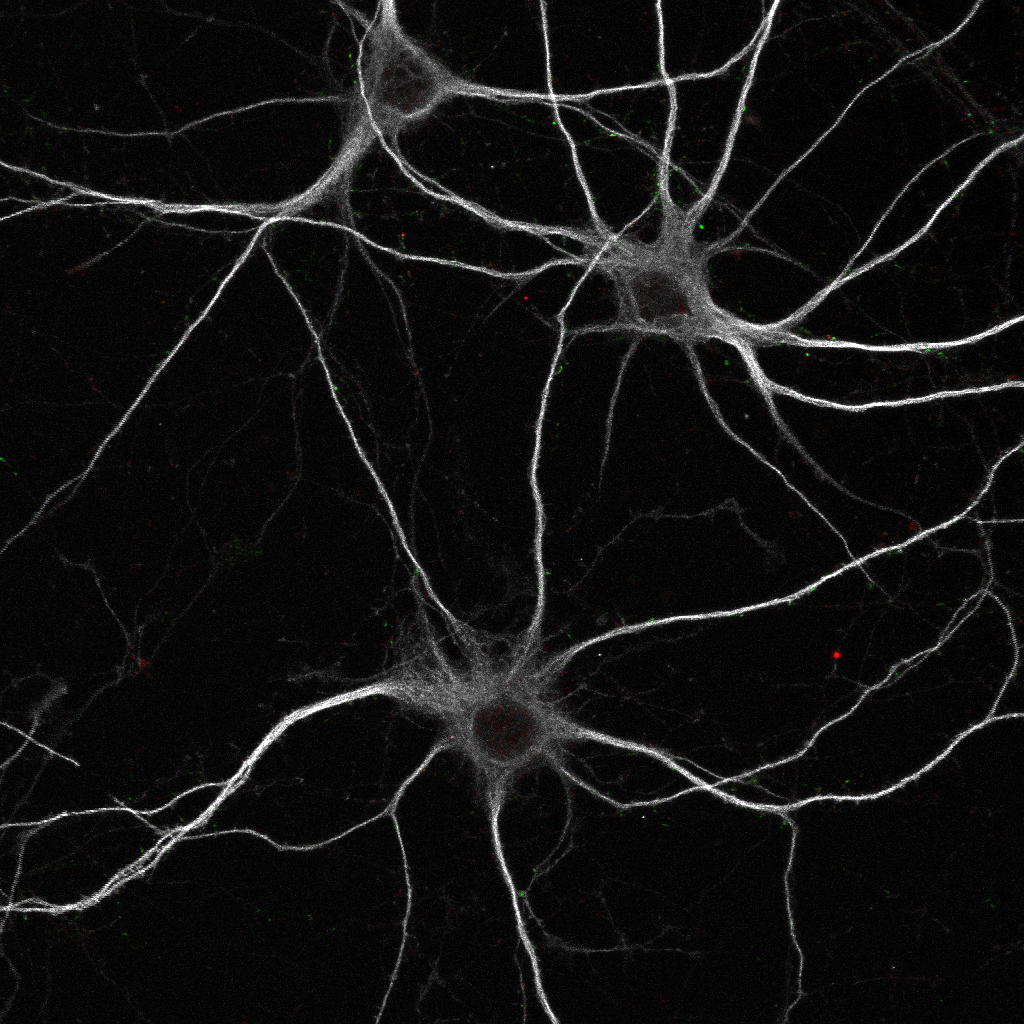

Supplement: Supplementary file 4 — Source data Fig. 2 [file 44321_2024_144_MOESM4_ESM.zip › Figure 2/2B/KO_UT/MERGE_KO_UT.tif]

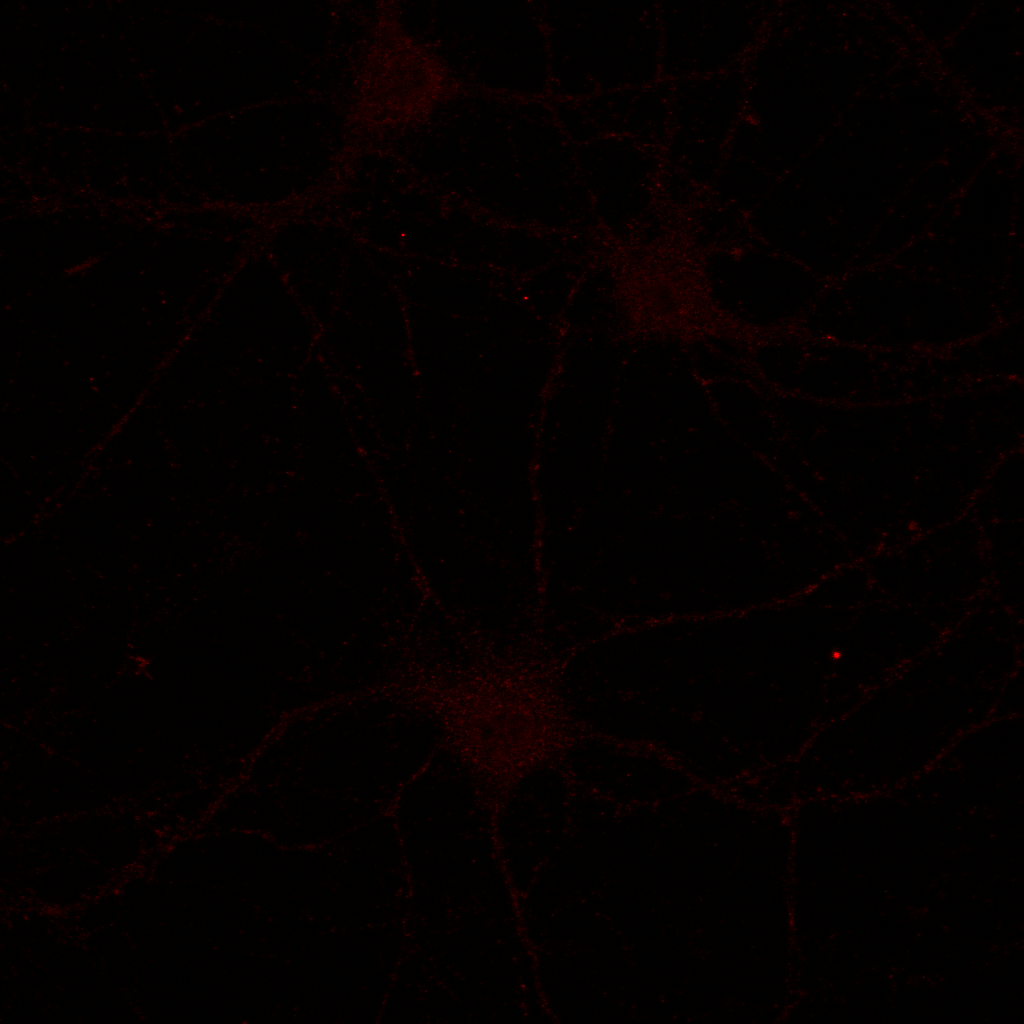

Supplement: Supplementary file 4 — Source data Fig. 2 [file 44321_2024_144_MOESM4_ESM.zip › Figure 2/2B/KO_UT/SHANK_KO_UT.tif]

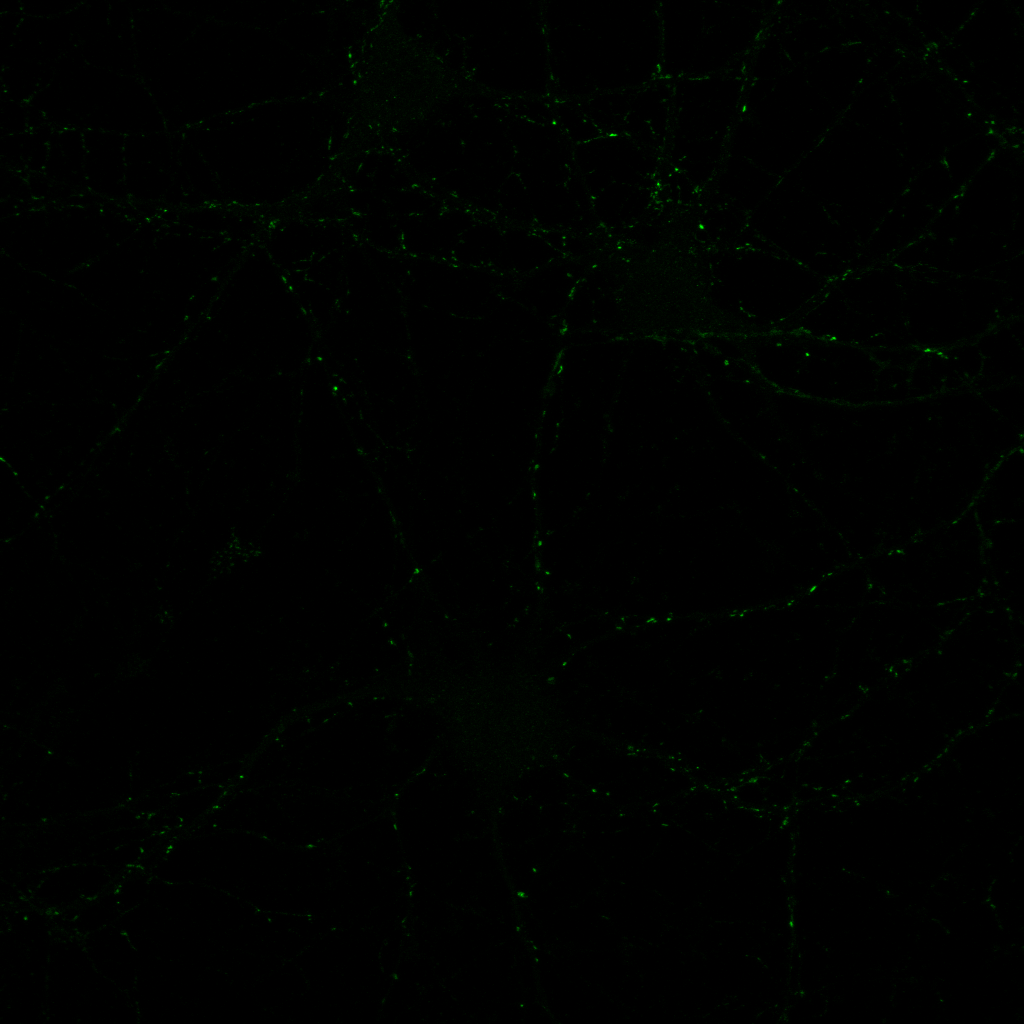

Supplement: Supplementary file 4 — Source data Fig. 2 [file 44321_2024_144_MOESM4_ESM.zip › Figure 2/2B/KO_UT/SYN_KO_UT.tif]

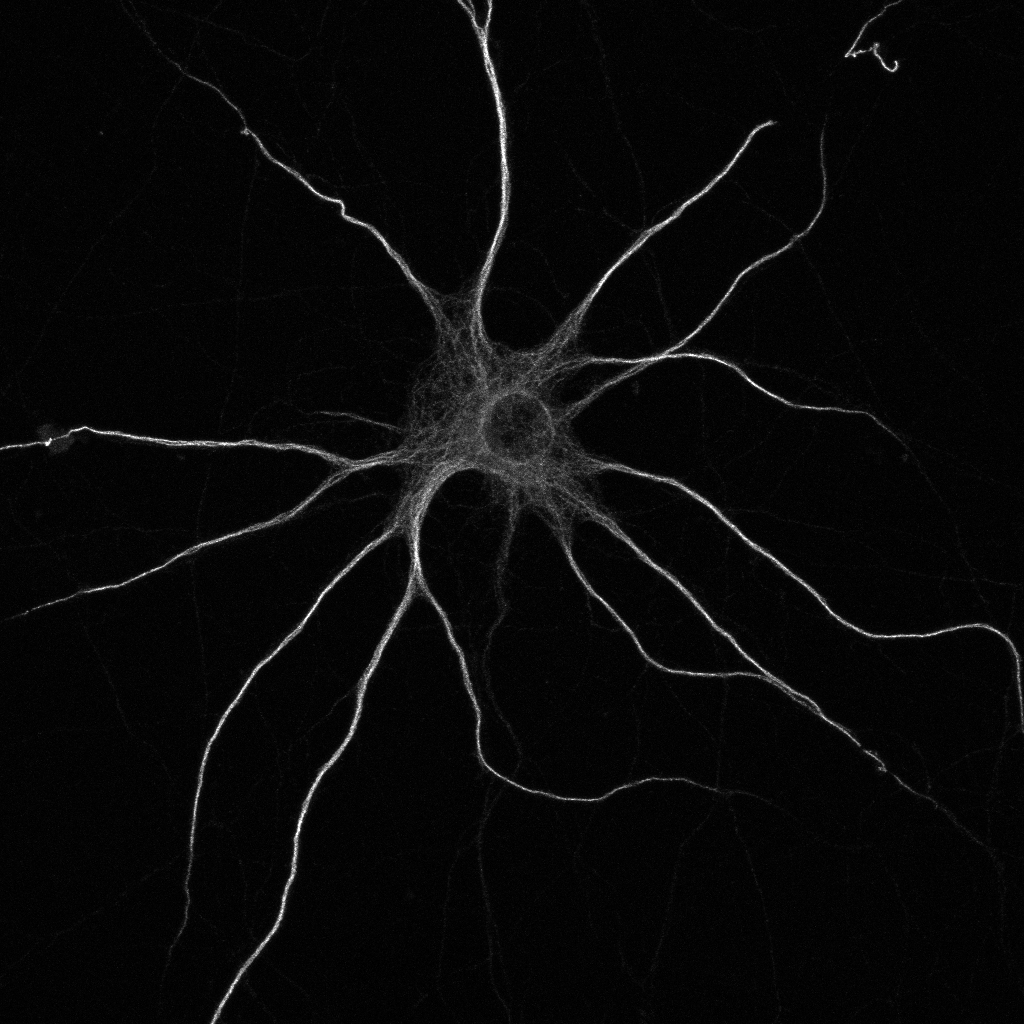

Supplement: Supplementary file 4 — Source data Fig. 2 [file 44321_2024_144_MOESM4_ESM.zip › Figure 2/2B/WT_UT/MAP2_WT_UT.tif]

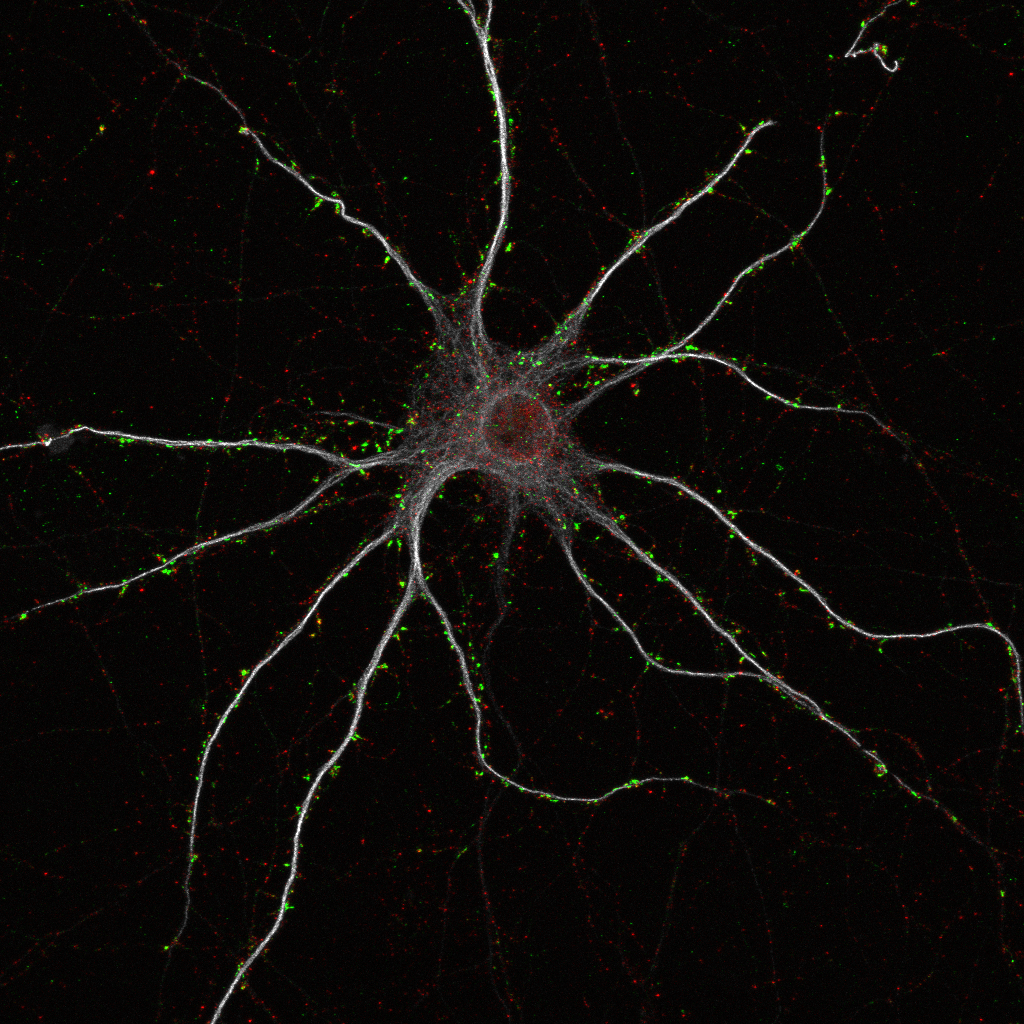

Supplement: Supplementary file 4 — Source data Fig. 2 [file 44321_2024_144_MOESM4_ESM.zip › Figure 2/2B/WT_UT/MERGE_WT_UT.tif]

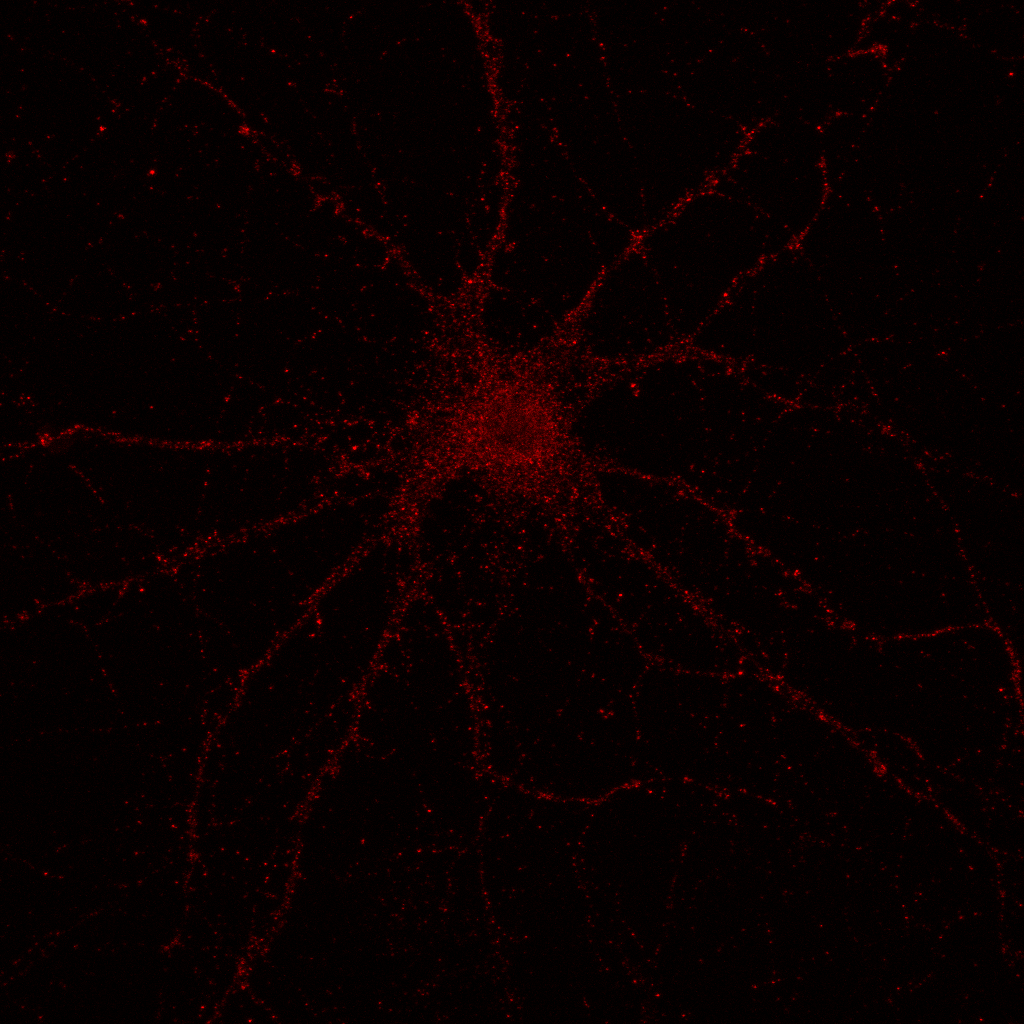

Supplement: Supplementary file 4 — Source data Fig. 2 [file 44321_2024_144_MOESM4_ESM.zip › Figure 2/2B/WT_UT/SHANK_WT_UT.tif]

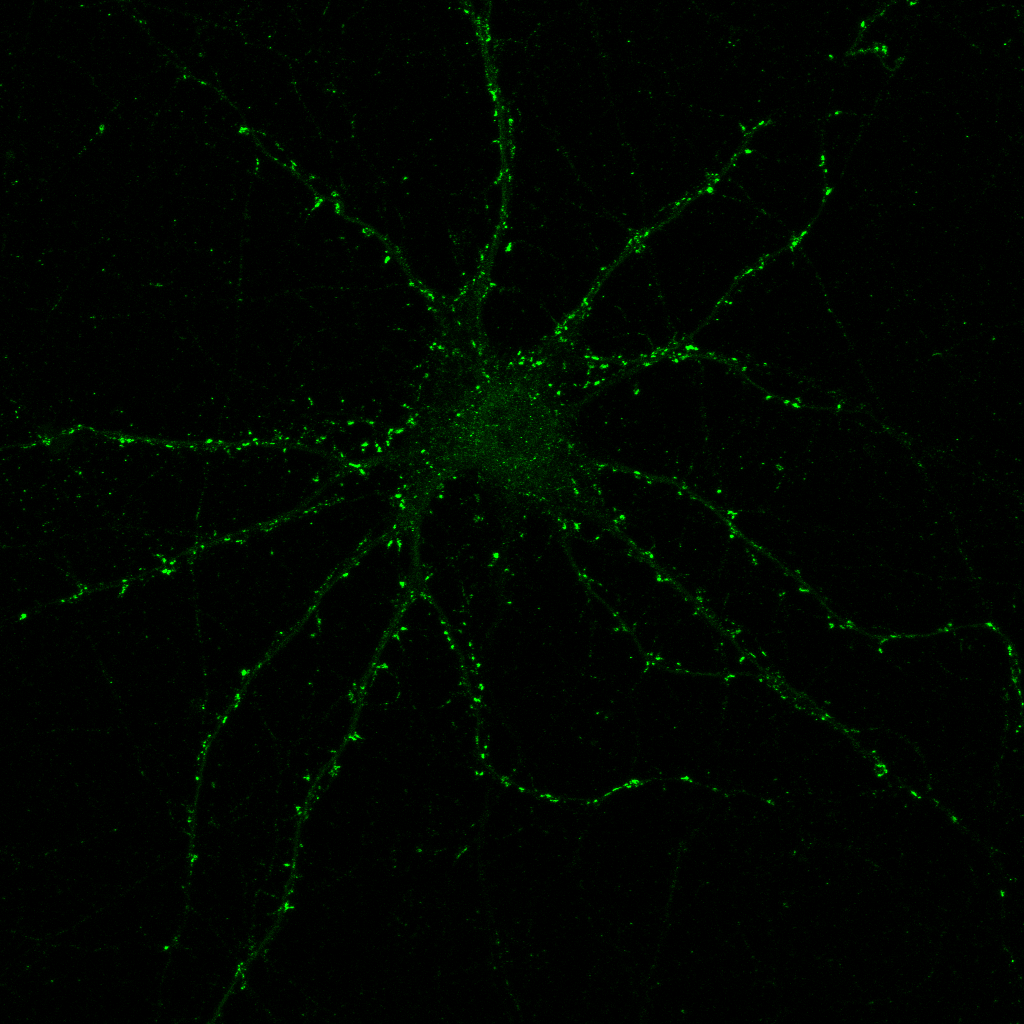

Supplement: Supplementary file 4 — Source data Fig. 2 [file 44321_2024_144_MOESM4_ESM.zip › Figure 2/2B/WT_UT/SYN_WT_UT.tif]

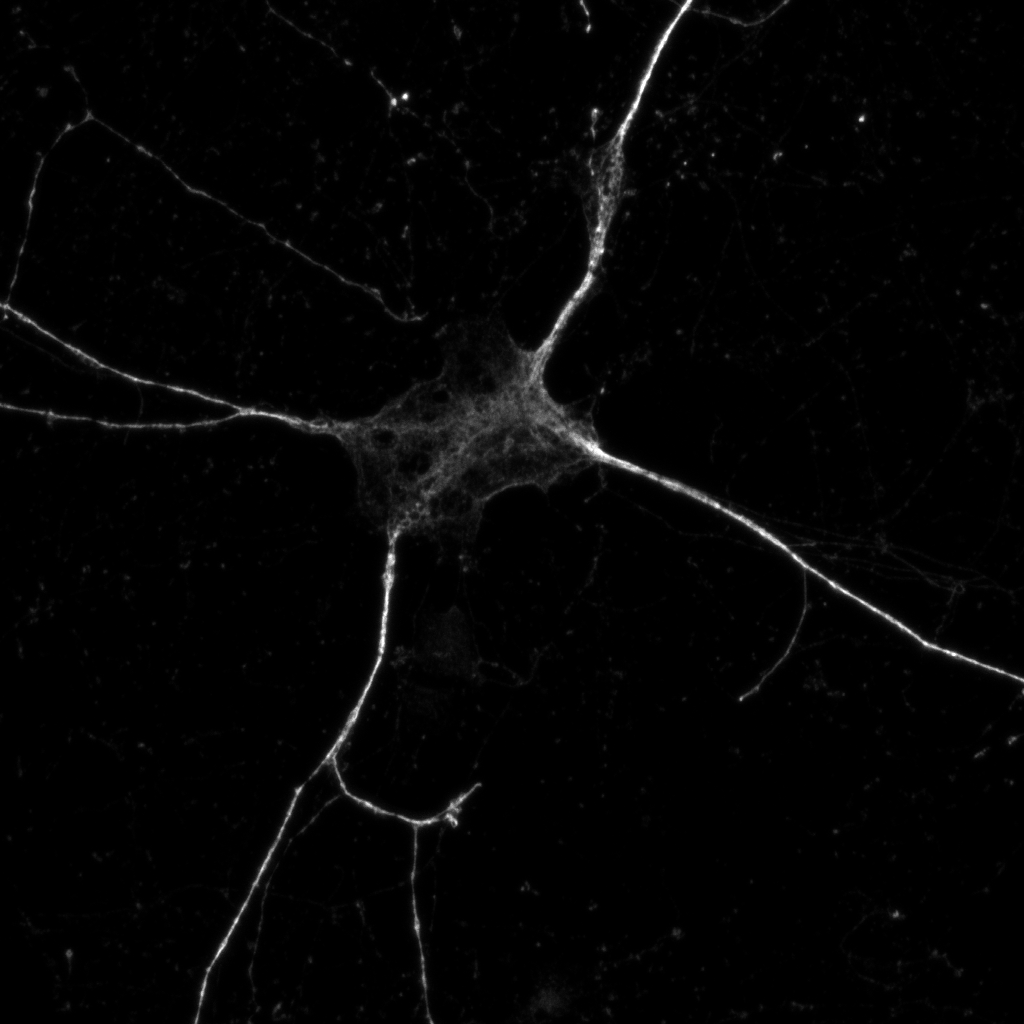

Supplement: Supplementary file 5 — Source data Fig. 3 [file 44321_2024_144_MOESM5_ESM.zip › Figure 3/3A/KO_CM_NIH3T3/MAP2_KO_CM_NIH3T3.tif]

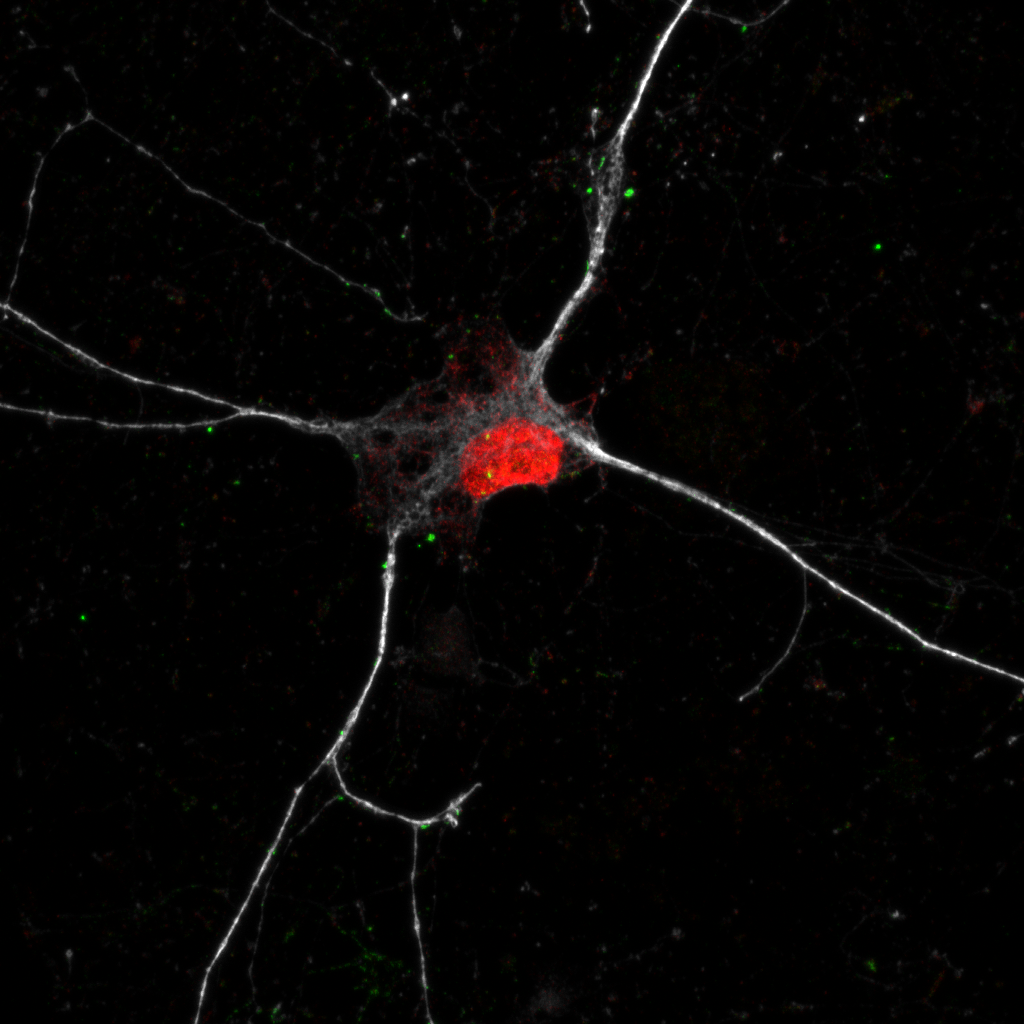

Supplement: Supplementary file 5 — Source data Fig. 3 [file 44321_2024_144_MOESM5_ESM.zip › Figure 3/3A/KO_CM_NIH3T3/MERGE_KO_CM_NIH3T3.tif]

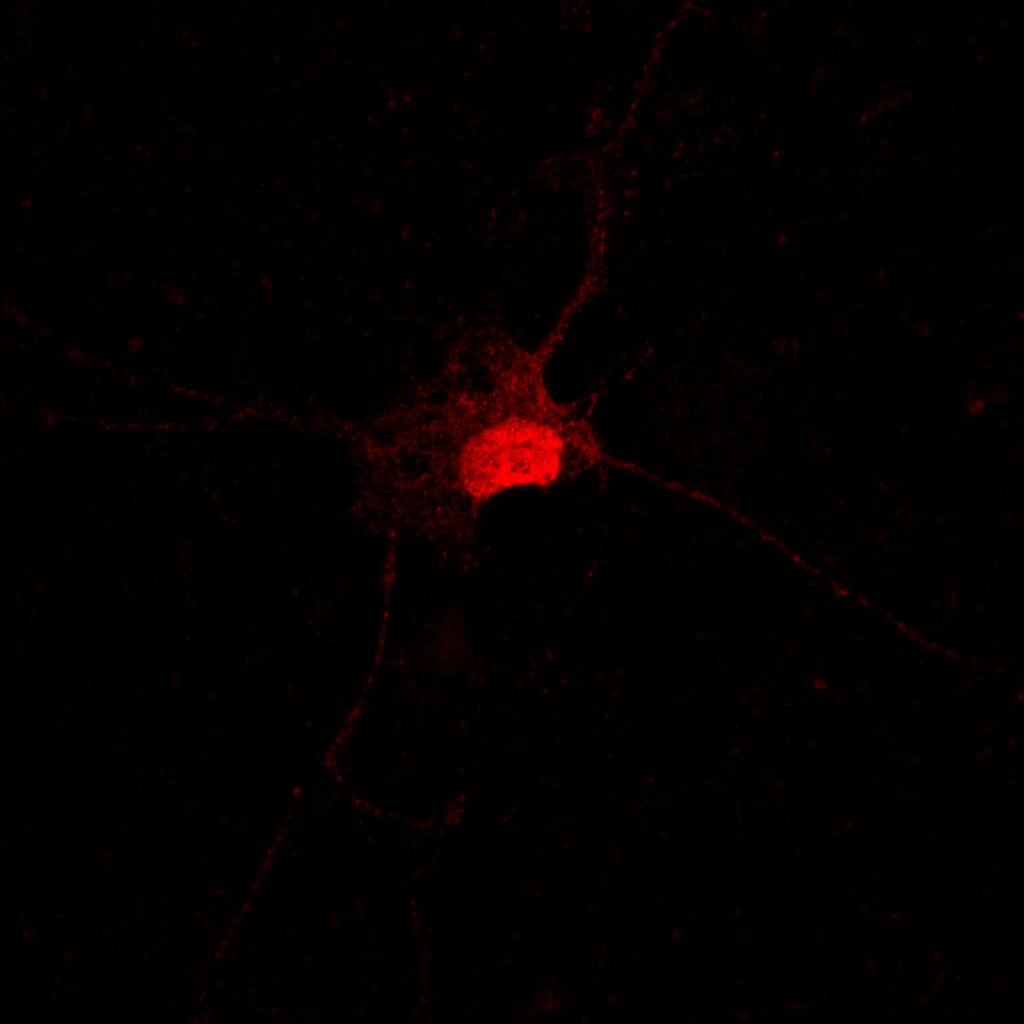

Supplement: Supplementary file 5 — Source data Fig. 3 [file 44321_2024_144_MOESM5_ESM.zip › Figure 3/3A/KO_CM_NIH3T3/SHANK_KO_CM_NIH3T3.tif]

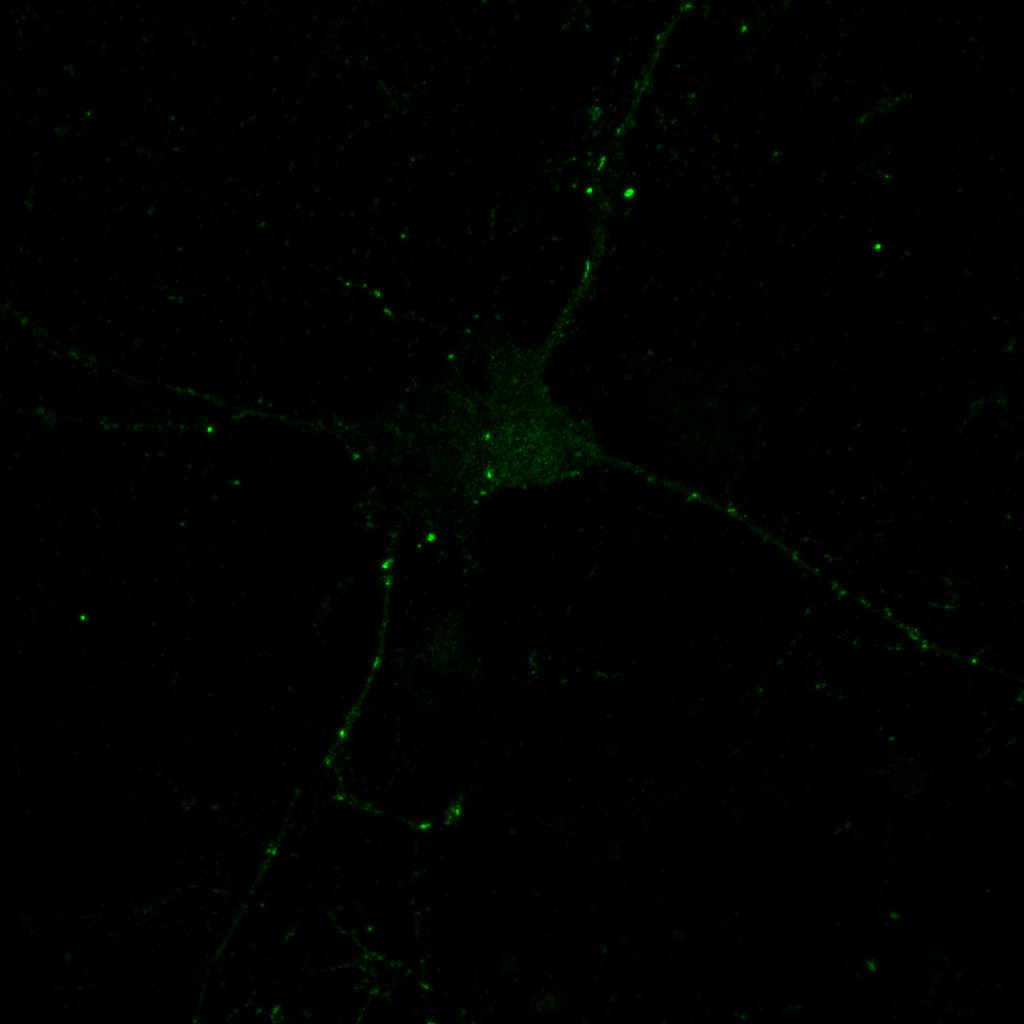

Supplement: Supplementary file 5 — Source data Fig. 3 [file 44321_2024_144_MOESM5_ESM.zip › Figure 3/3A/KO_CM_NIH3T3/SYN_KO_CM_NIH3T3.tif]

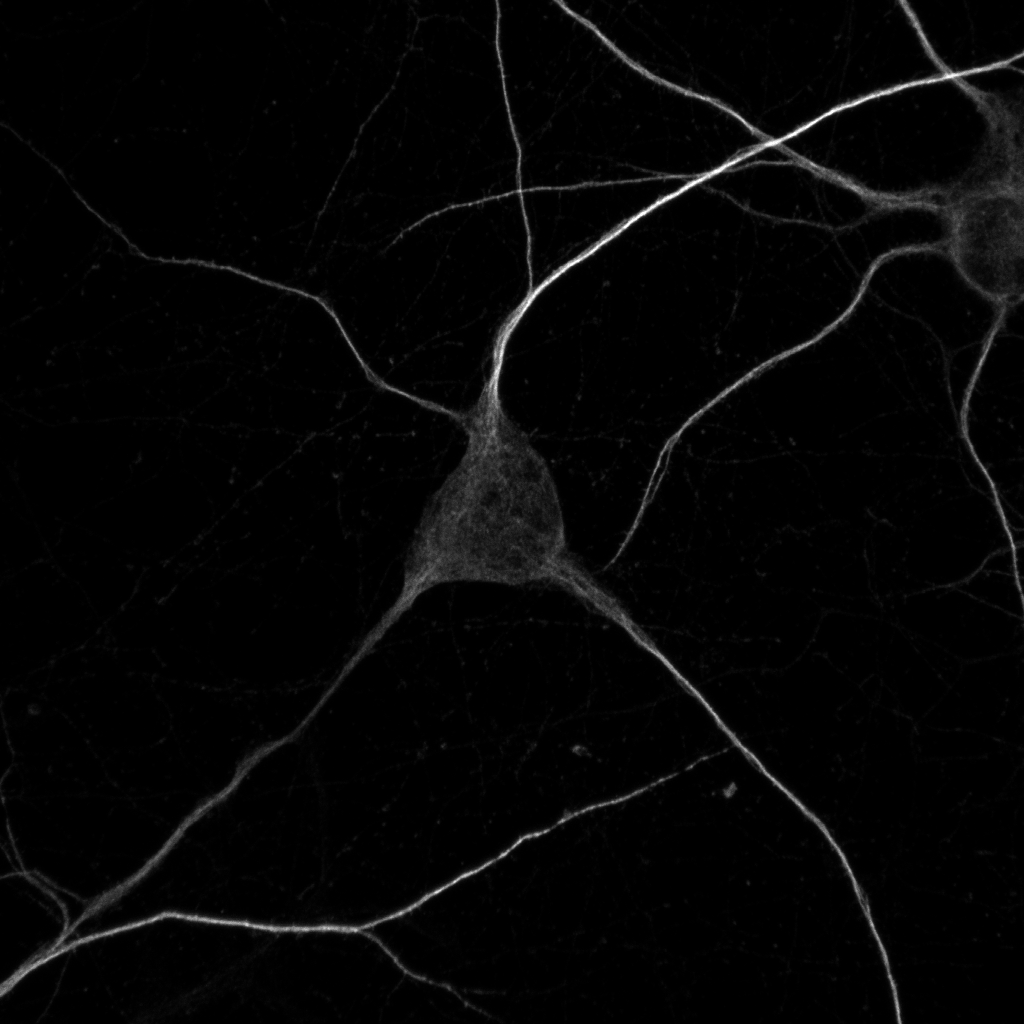

Supplement: Supplementary file 5 — Source data Fig. 3 [file 44321_2024_144_MOESM5_ESM.zip › Figure 3/3A/KO_CM_NPCs_KO/MAP2_KO_CM_NPCs_KO.tif]

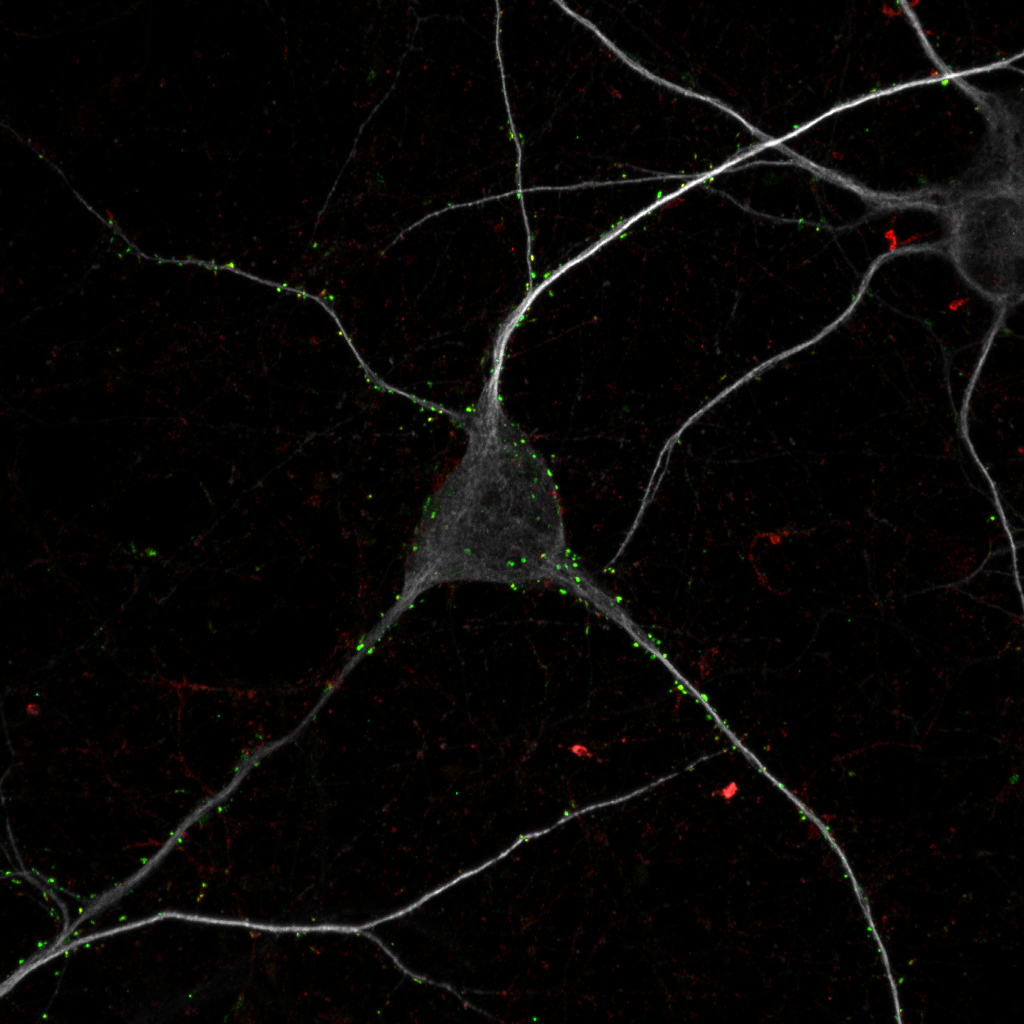

Supplement: Supplementary file 5 — Source data Fig. 3 [file 44321_2024_144_MOESM5_ESM.zip › Figure 3/3A/KO_CM_NPCs_KO/MERGE_KO_CM_NPCs_KO.tif]

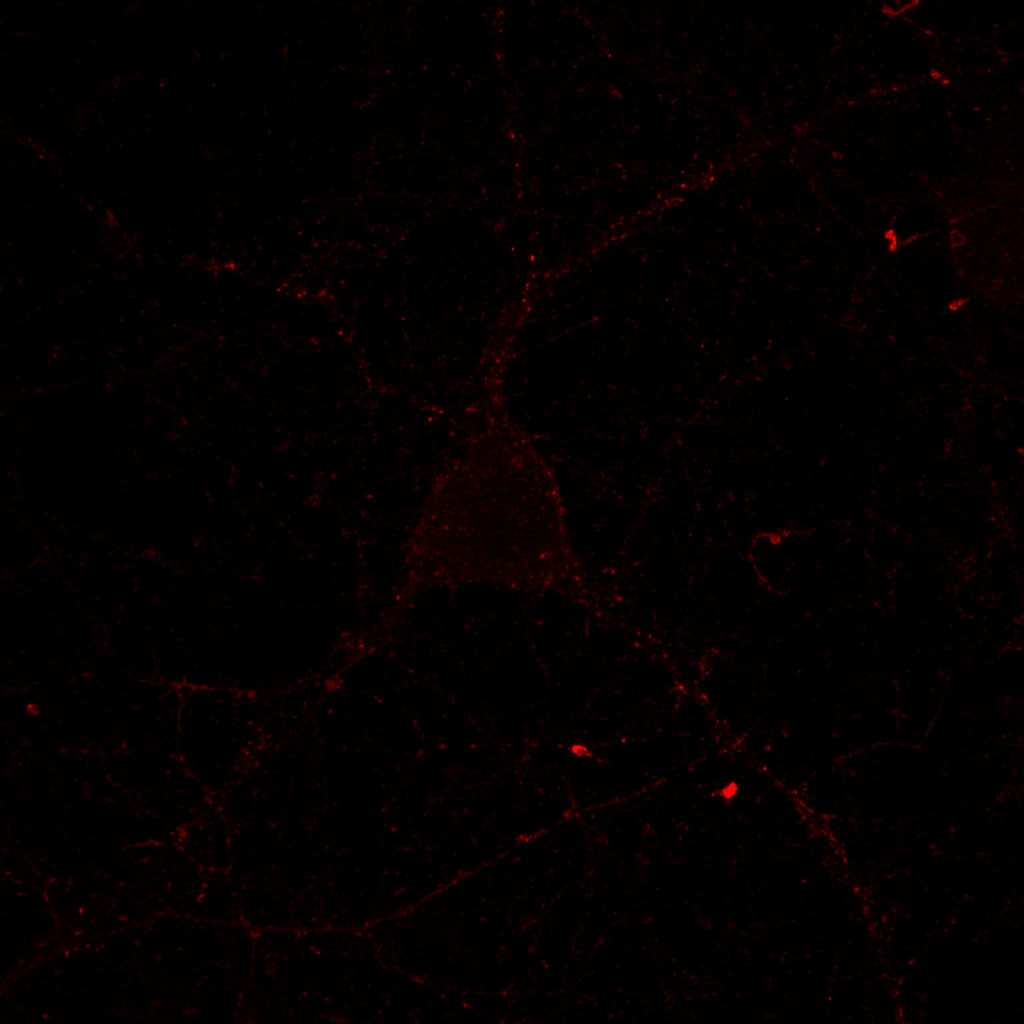

Supplement: Supplementary file 5 — Source data Fig. 3 [file 44321_2024_144_MOESM5_ESM.zip › Figure 3/3A/KO_CM_NPCs_KO/SHANK_KO_CM_NPCs_KO.tif]

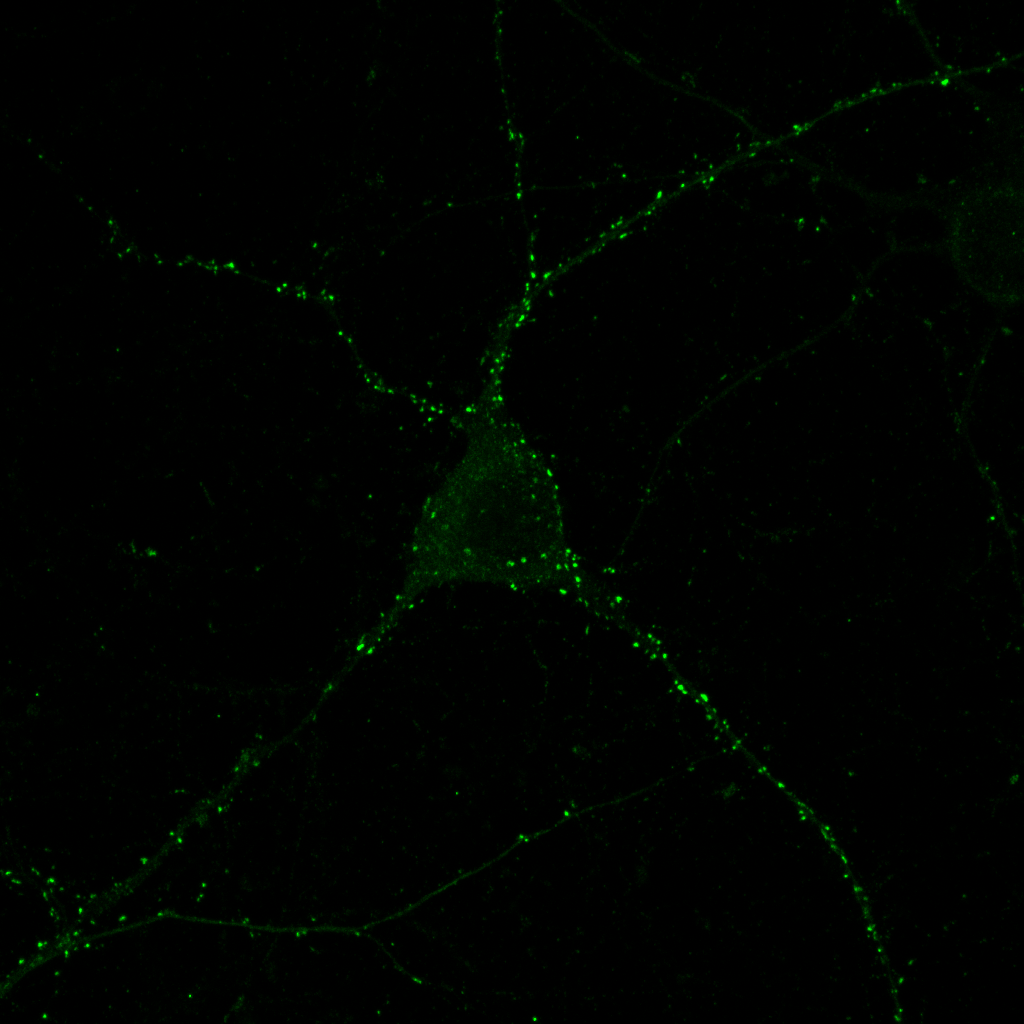

Supplement: Supplementary file 5 — Source data Fig. 3 [file 44321_2024_144_MOESM5_ESM.zip › Figure 3/3A/KO_CM_NPCs_KO/SYN_KO_CM_NPCs_KO.tif]

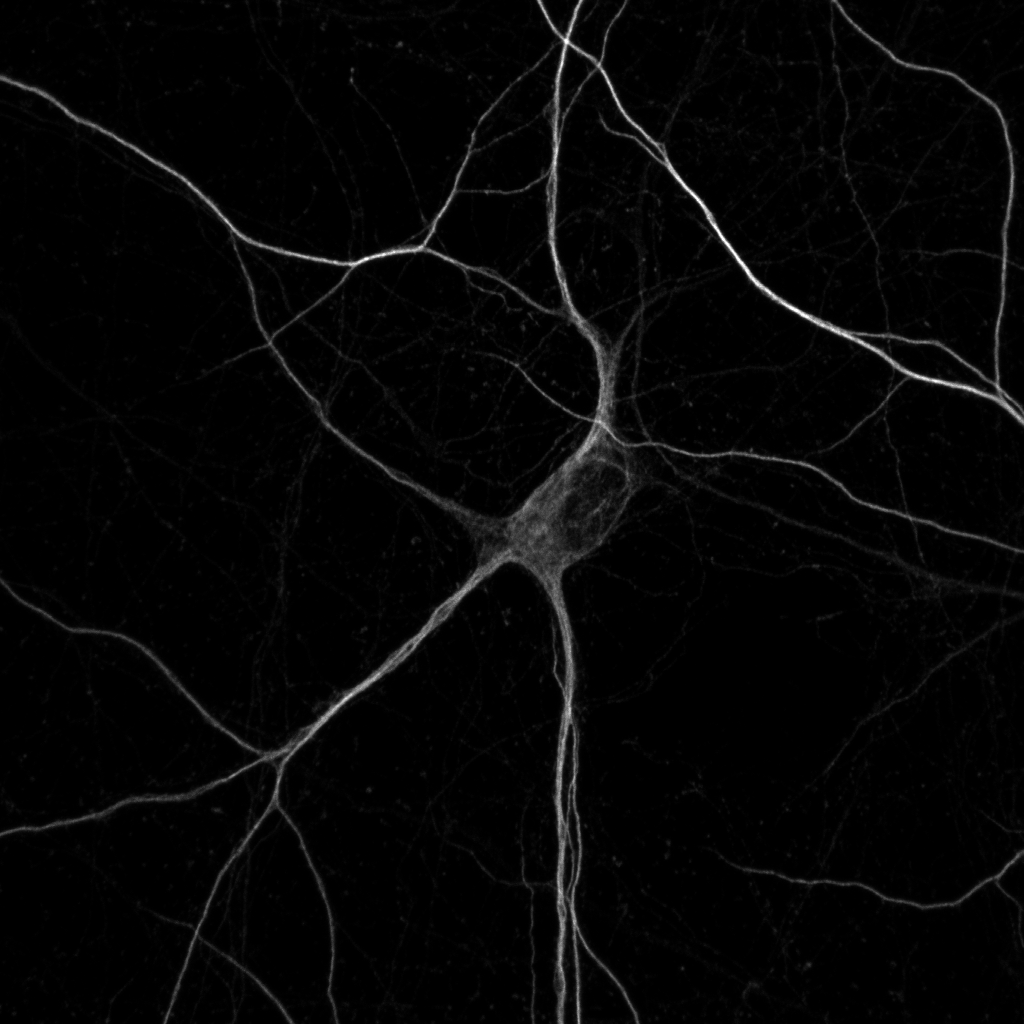

Supplement: Supplementary file 5 — Source data Fig. 3 [file 44321_2024_144_MOESM5_ESM.zip › Figure 3/3A/KO_CM_NPCs_WT/MAP2_KO_CM_NPCs_WT.tif]

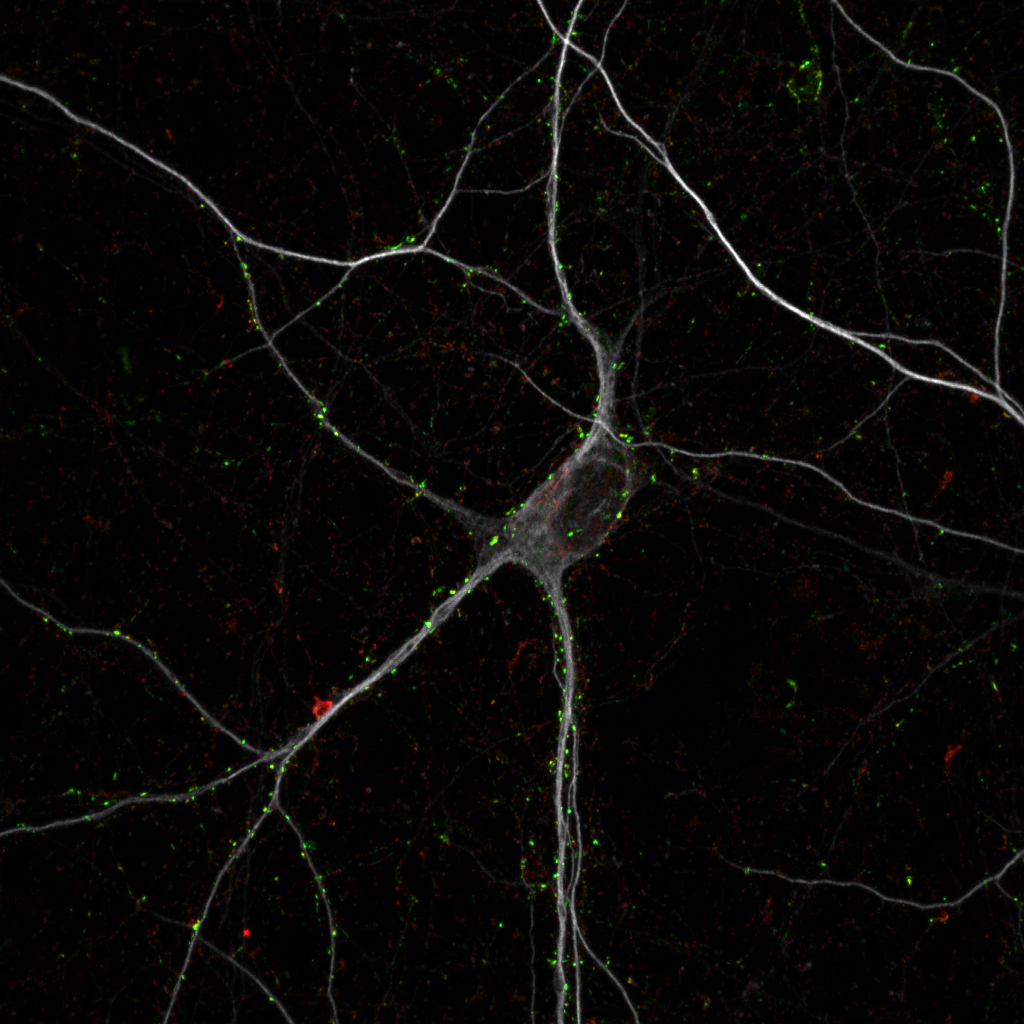

Supplement: Supplementary file 5 — Source data Fig. 3 [file 44321_2024_144_MOESM5_ESM.zip › Figure 3/3A/KO_CM_NPCs_WT/MERGE_KO_CM_NPCs_WT.tif]

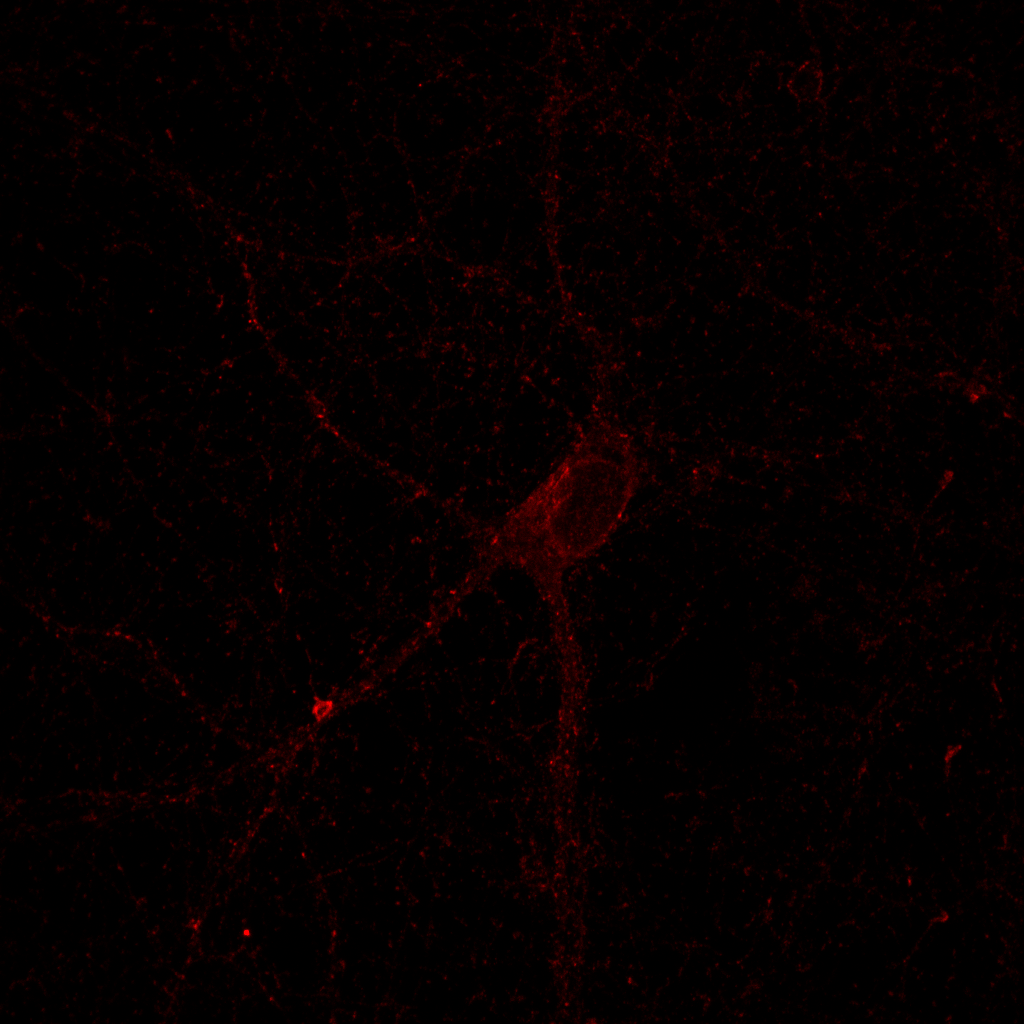

Supplement: Supplementary file 5 — Source data Fig. 3 [file 44321_2024_144_MOESM5_ESM.zip › Figure 3/3A/KO_CM_NPCs_WT/SHANK_KO_CM_NPCs_WT.tif]

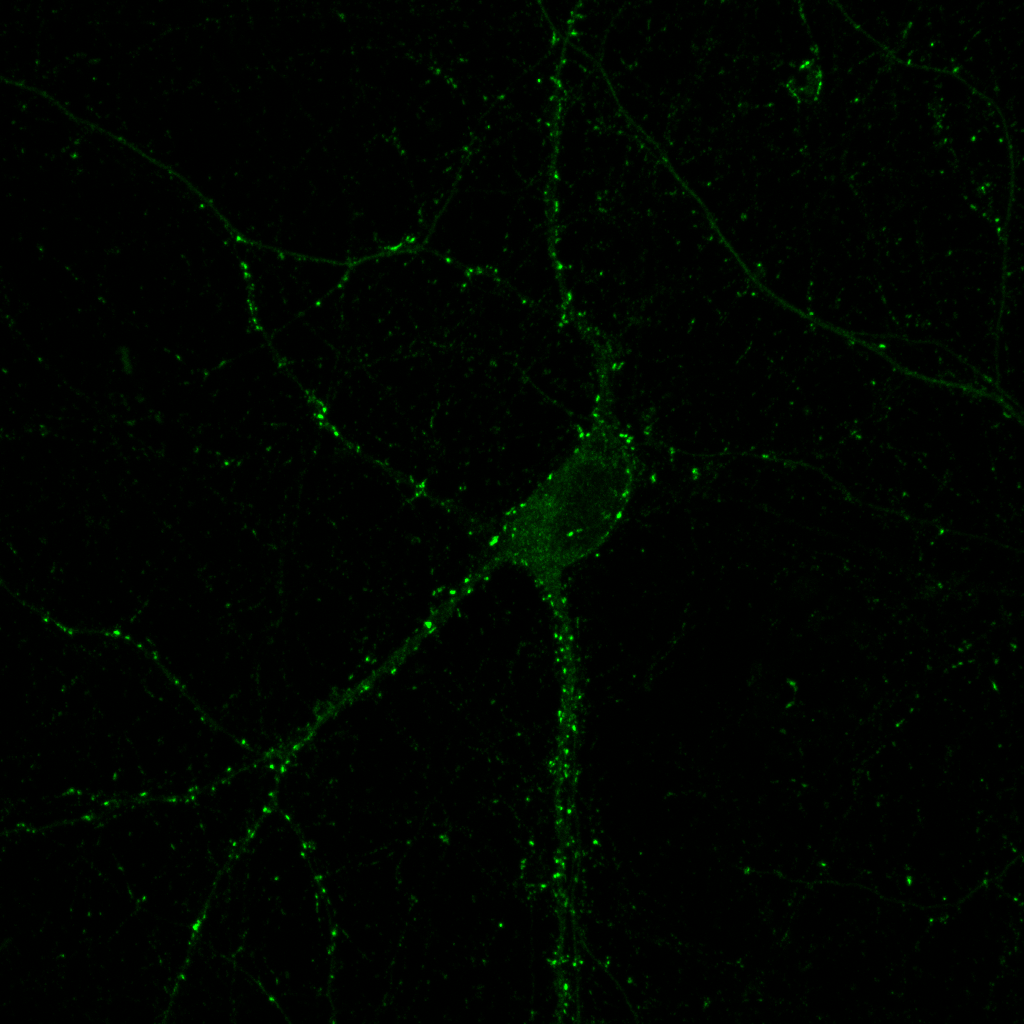

Supplement: Supplementary file 5 — Source data Fig. 3 [file 44321_2024_144_MOESM5_ESM.zip › Figure 3/3A/KO_CM_NPCs_WT/SYN_KO_CM_NPCs_WT.tif]

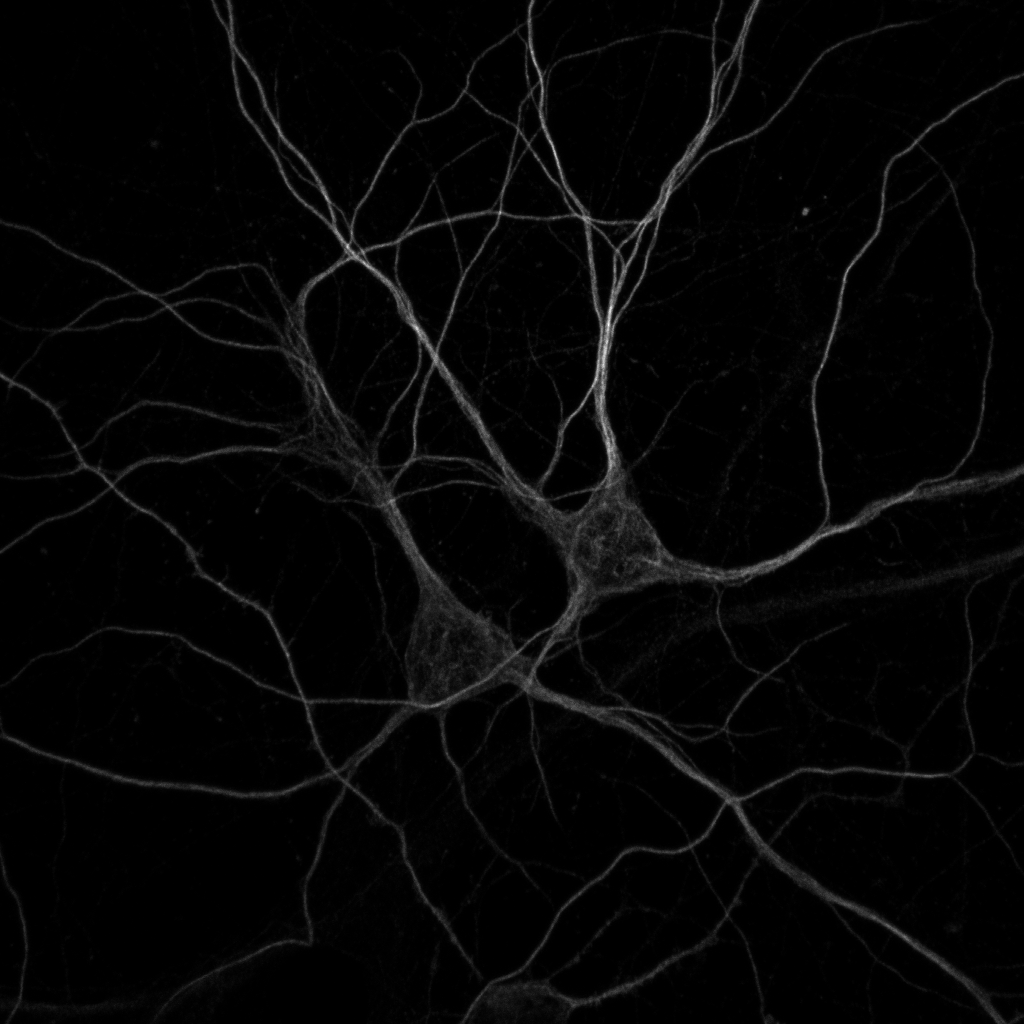

Supplement: Supplementary file 5 — Source data Fig. 3 [file 44321_2024_144_MOESM5_ESM.zip › Figure 3/3A/KO_UT/MAP2_KO_UT.tif]

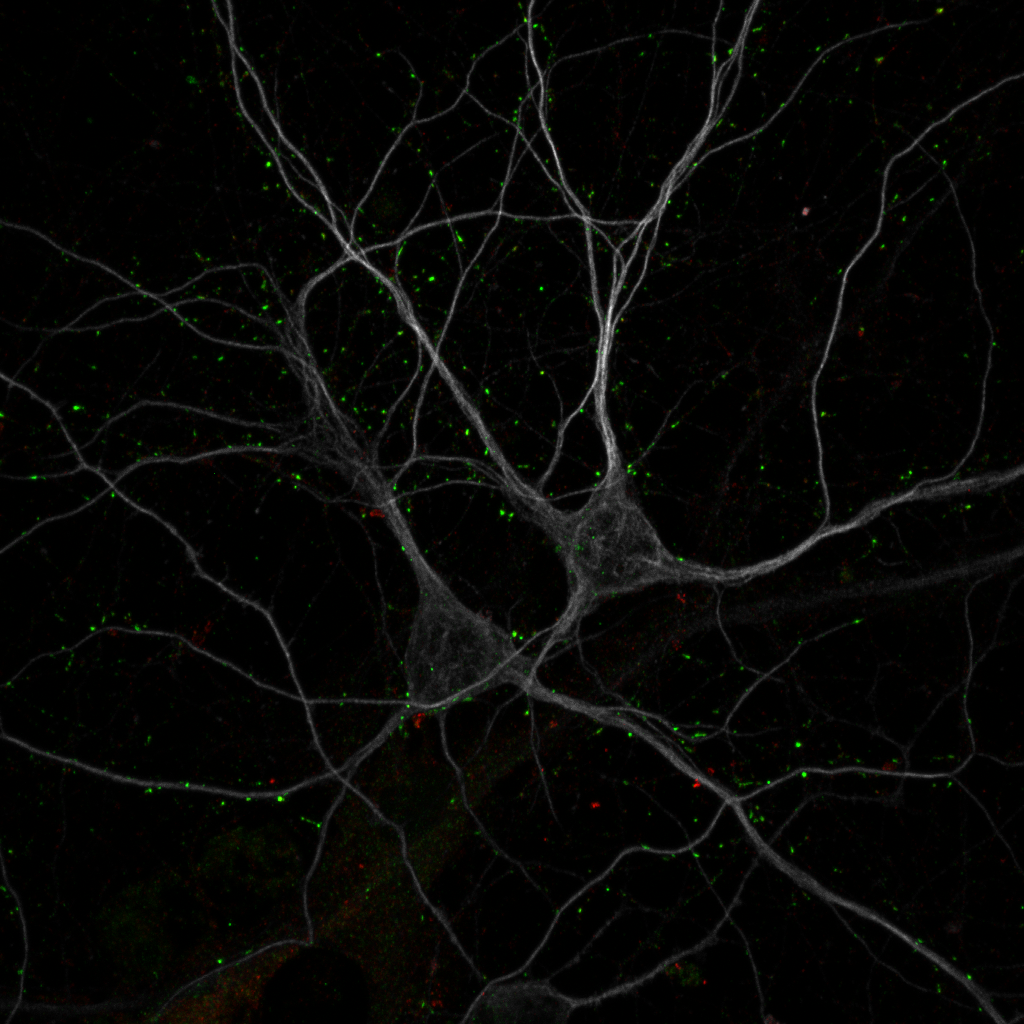

Supplement: Supplementary file 5 — Source data Fig. 3 [file 44321_2024_144_MOESM5_ESM.zip › Figure 3/3A/KO_UT/MERGE_KO_UT.tif]

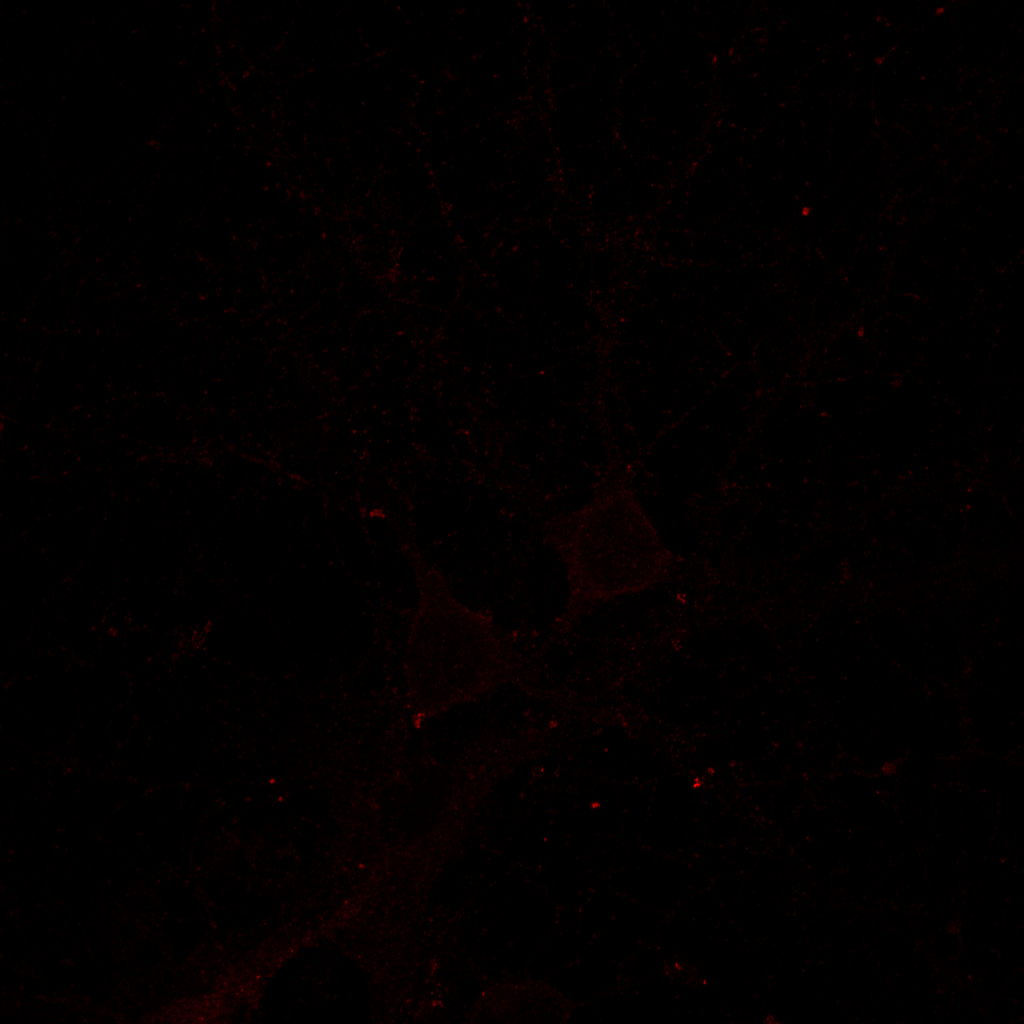

Supplement: Supplementary file 5 — Source data Fig. 3 [file 44321_2024_144_MOESM5_ESM.zip › Figure 3/3A/KO_UT/SHANK_KO_UT.tif]

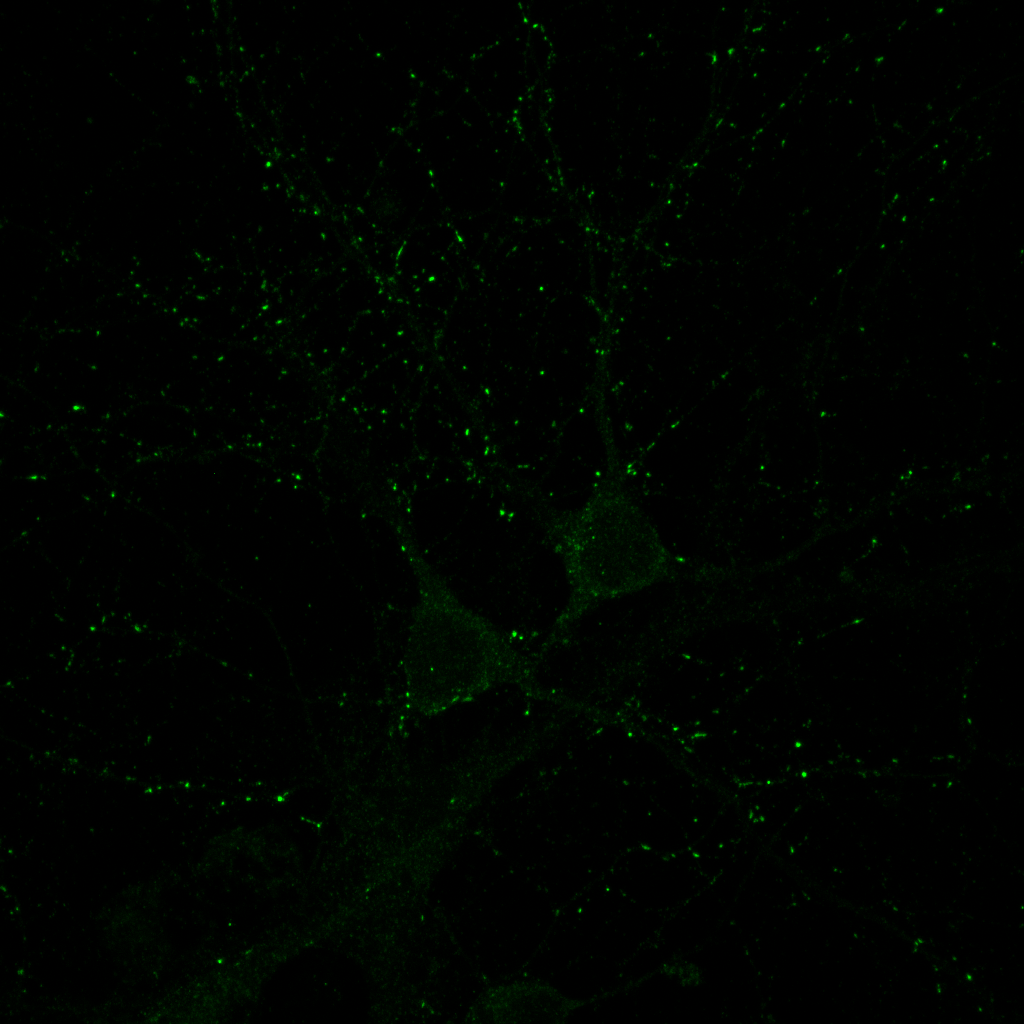

Supplement: Supplementary file 5 — Source data Fig. 3 [file 44321_2024_144_MOESM5_ESM.zip › Figure 3/3A/KO_UT/SYN_KO_UT.tif]

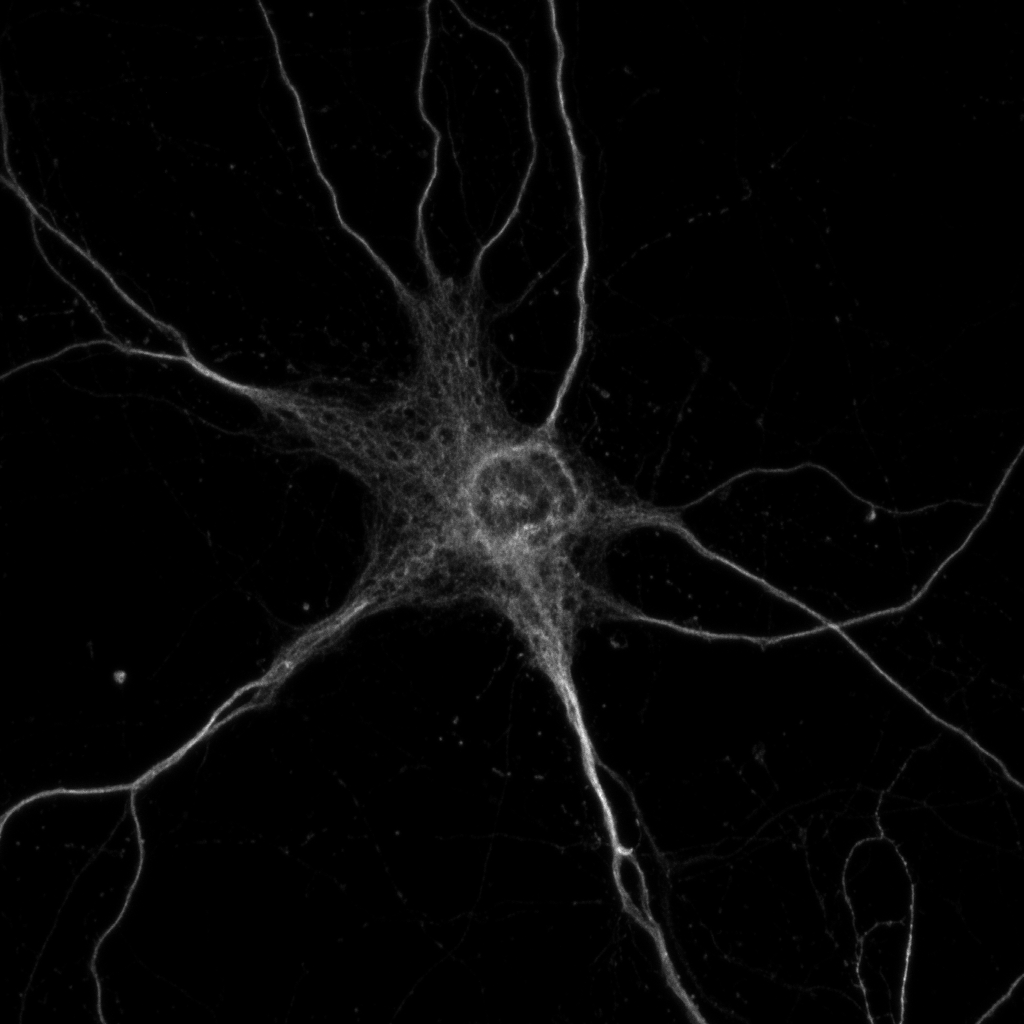

Supplement: Supplementary file 5 — Source data Fig. 3 [file 44321_2024_144_MOESM5_ESM.zip › Figure 3/3A/WT_UT/MAP2_WT_UT.tif]

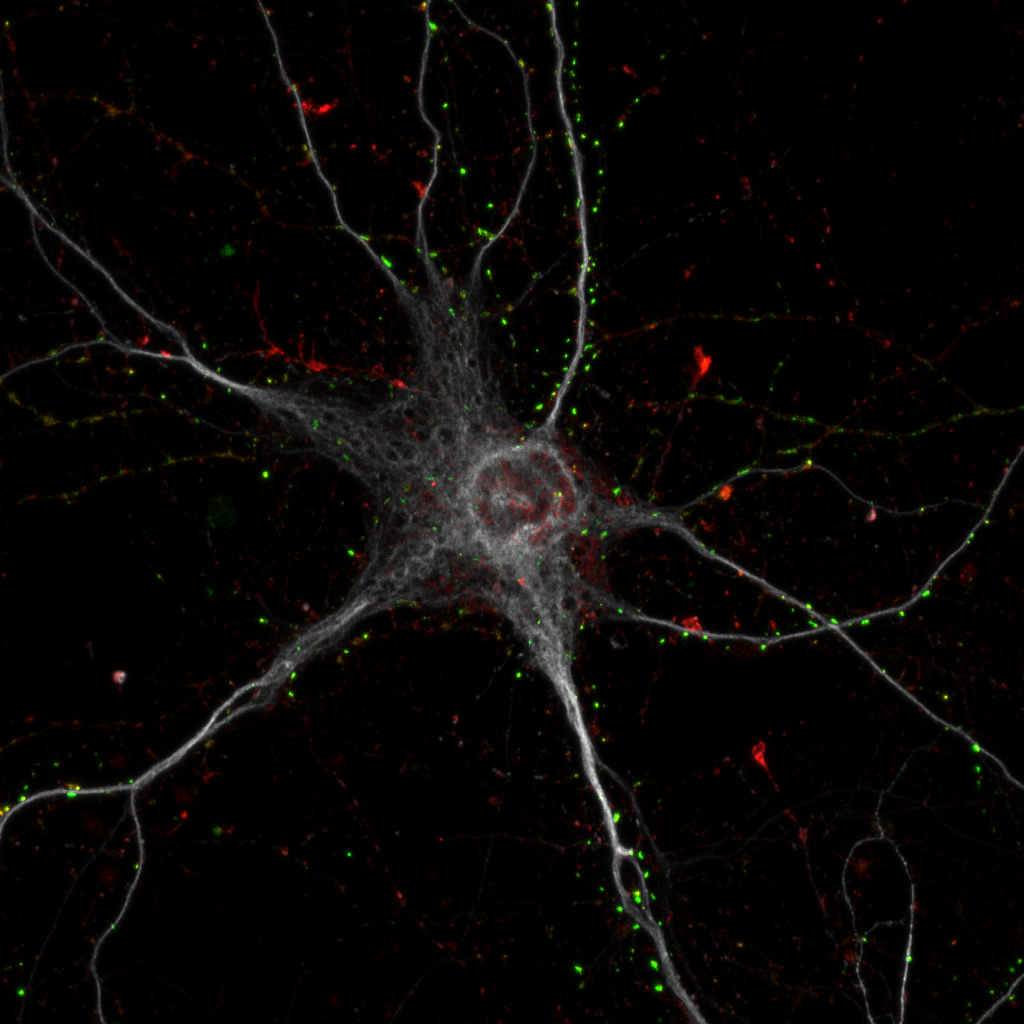

Supplement: Supplementary file 5 — Source data Fig. 3 [file 44321_2024_144_MOESM5_ESM.zip › Figure 3/3A/WT_UT/MERGE_WT_UT.tif]

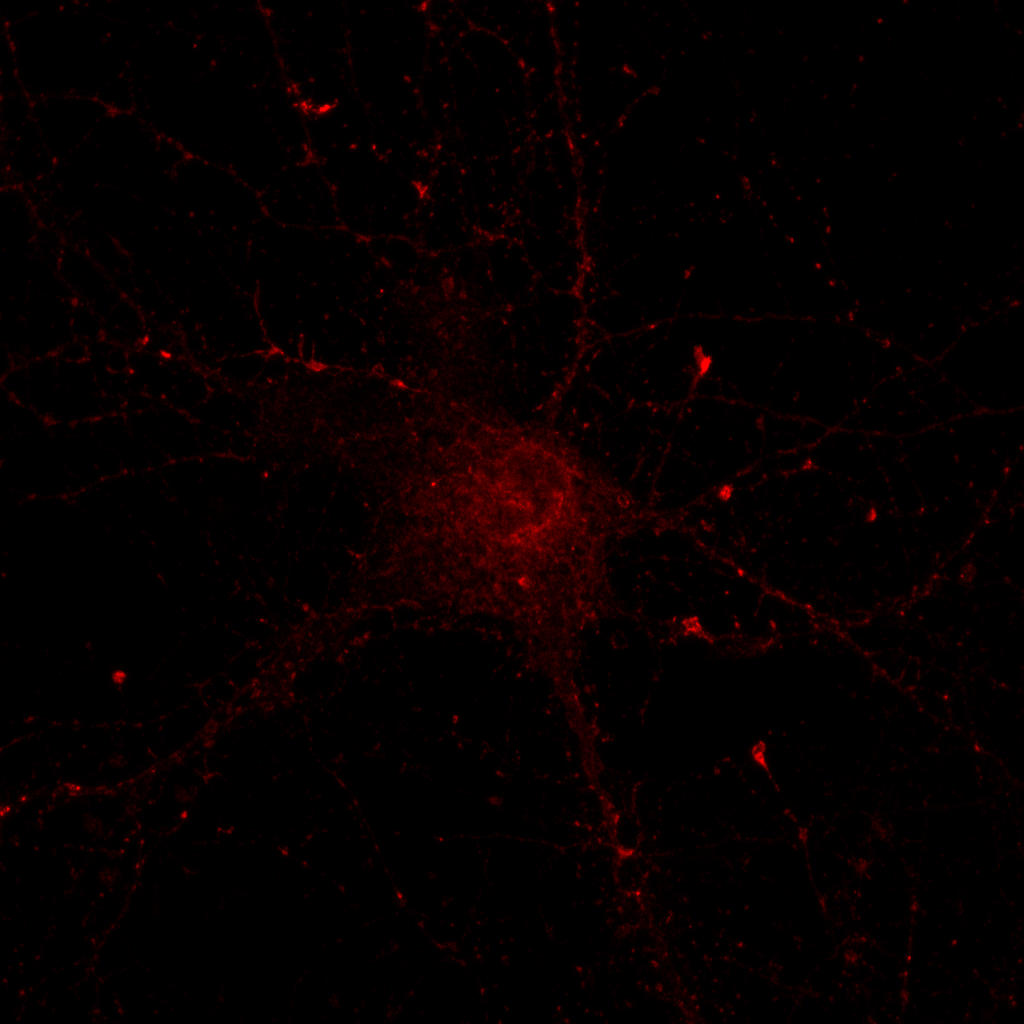

Supplement: Supplementary file 5 — Source data Fig. 3 [file 44321_2024_144_MOESM5_ESM.zip › Figure 3/3A/WT_UT/SHANK_WT_UT.tif]

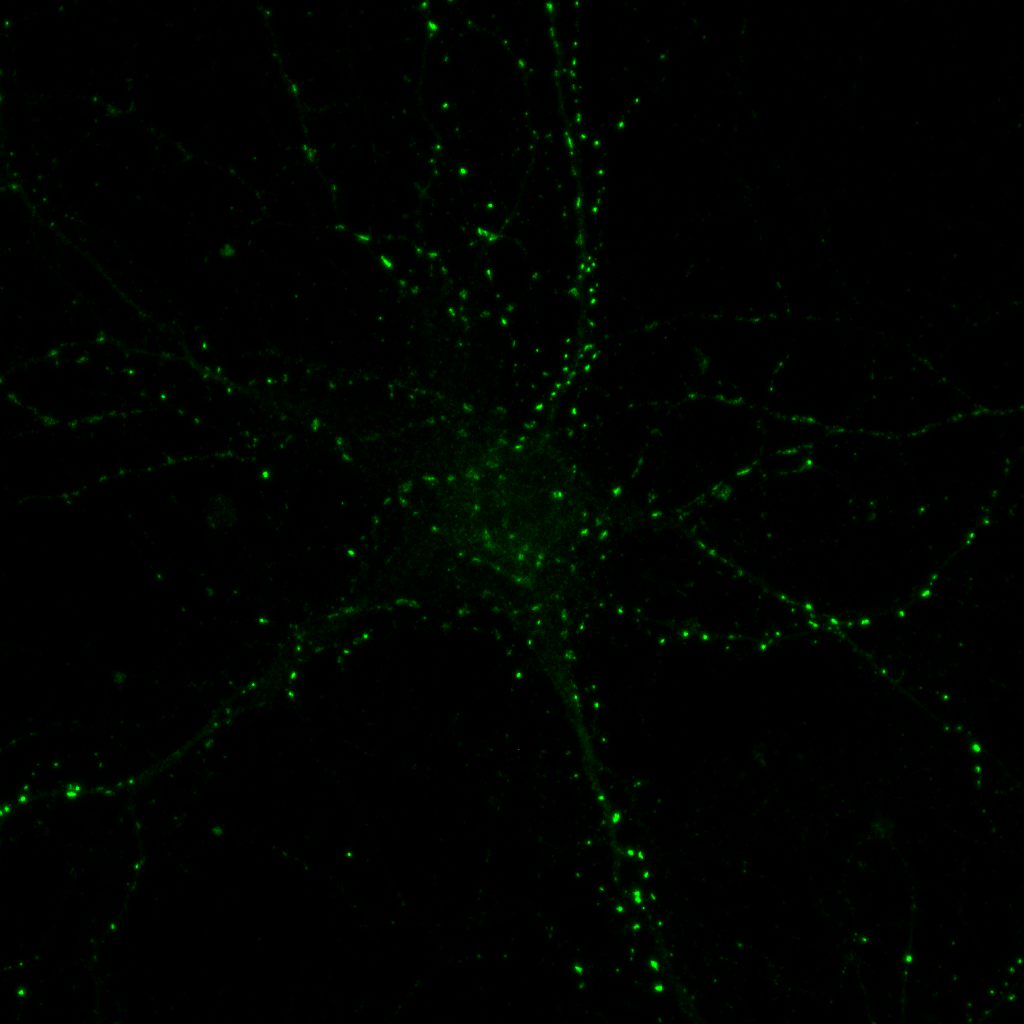

Supplement: Supplementary file 5 — Source data Fig. 3 [file 44321_2024_144_MOESM5_ESM.zip › Figure 3/3A/WT_UT/SYN_WT_UT.tif]

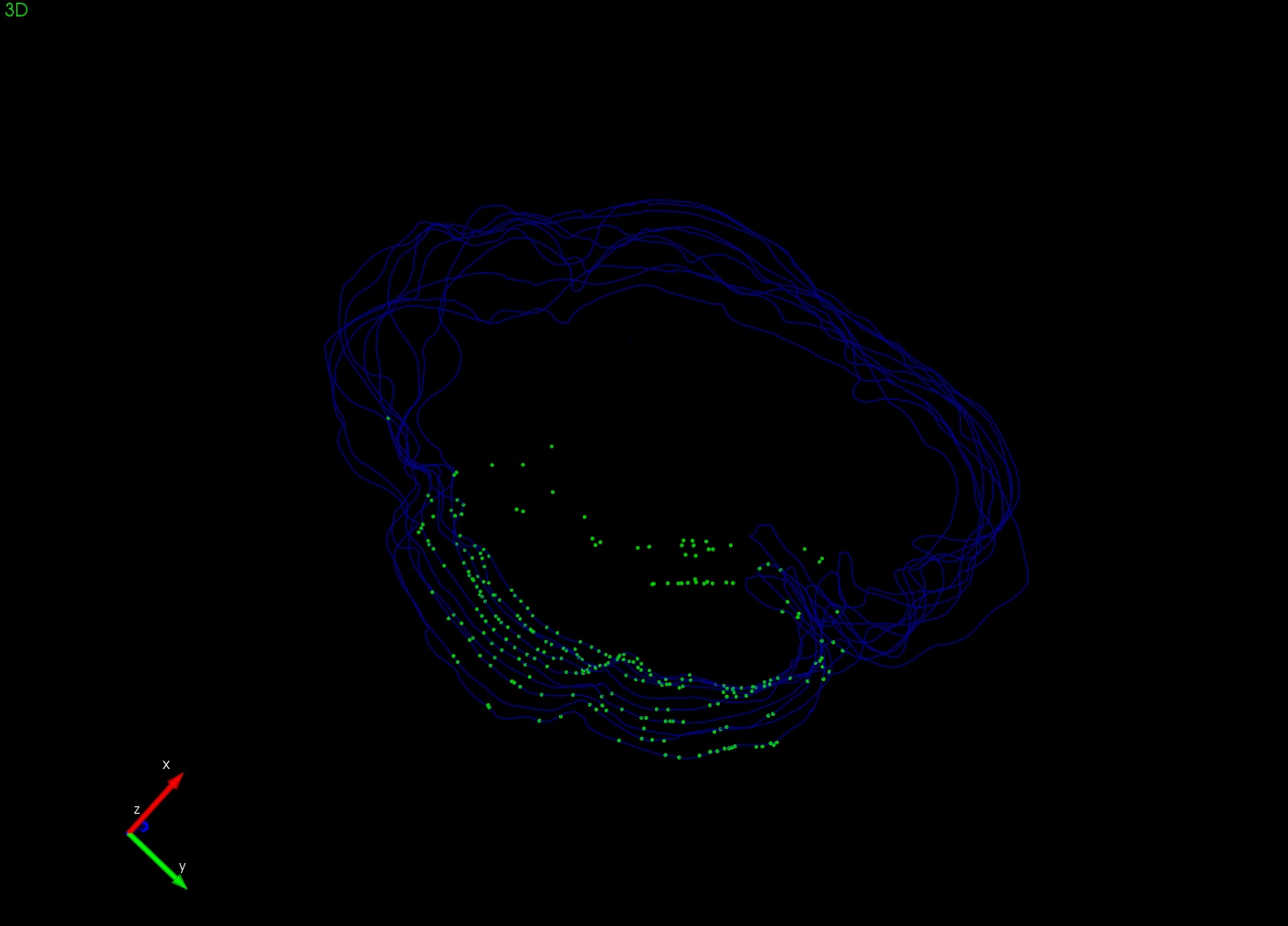

Supplement: Supplementary file 7 — Source data Fig. 5 [file 44321_2024_144_MOESM7_ESM.zip › Figure 5/5A/4A.jpg]

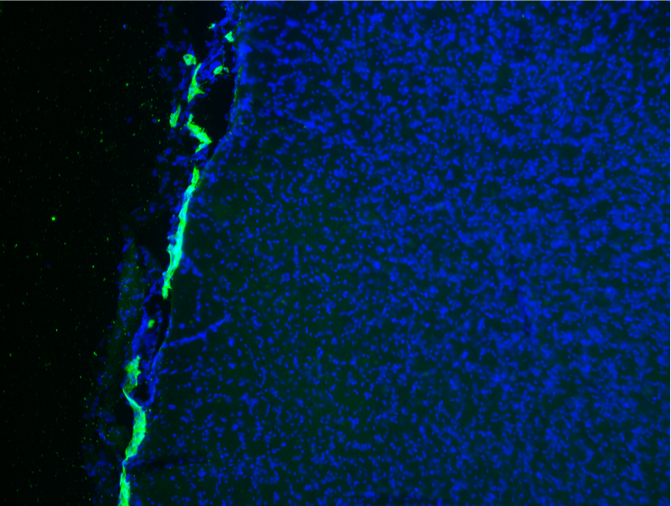

Supplement: Supplementary file 7 — Source data Fig. 5 [file 44321_2024_144_MOESM7_ESM.zip › Figure 5/5B/5B_10days.tif]

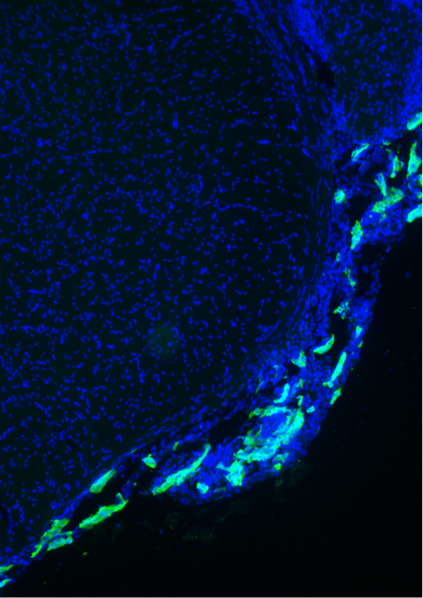

Supplement: Supplementary file 7 — Source data Fig. 5 [file 44321_2024_144_MOESM7_ESM.zip › Figure 5/5B/5B_96h.tif]

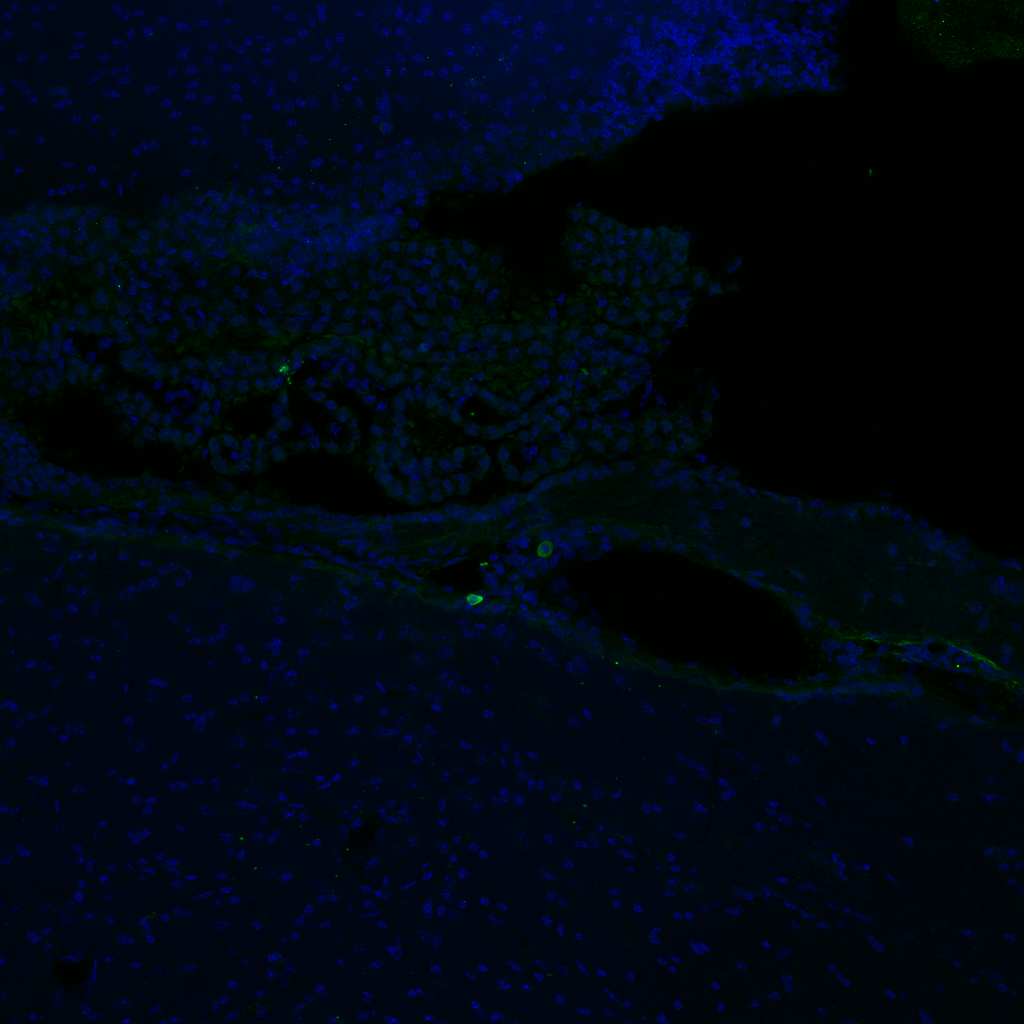

Supplement: Supplementary file 7 — Source data Fig. 5 [file 44321_2024_144_MOESM7_ESM.zip › Figure 5/5B/5B_KO 20days.tif]

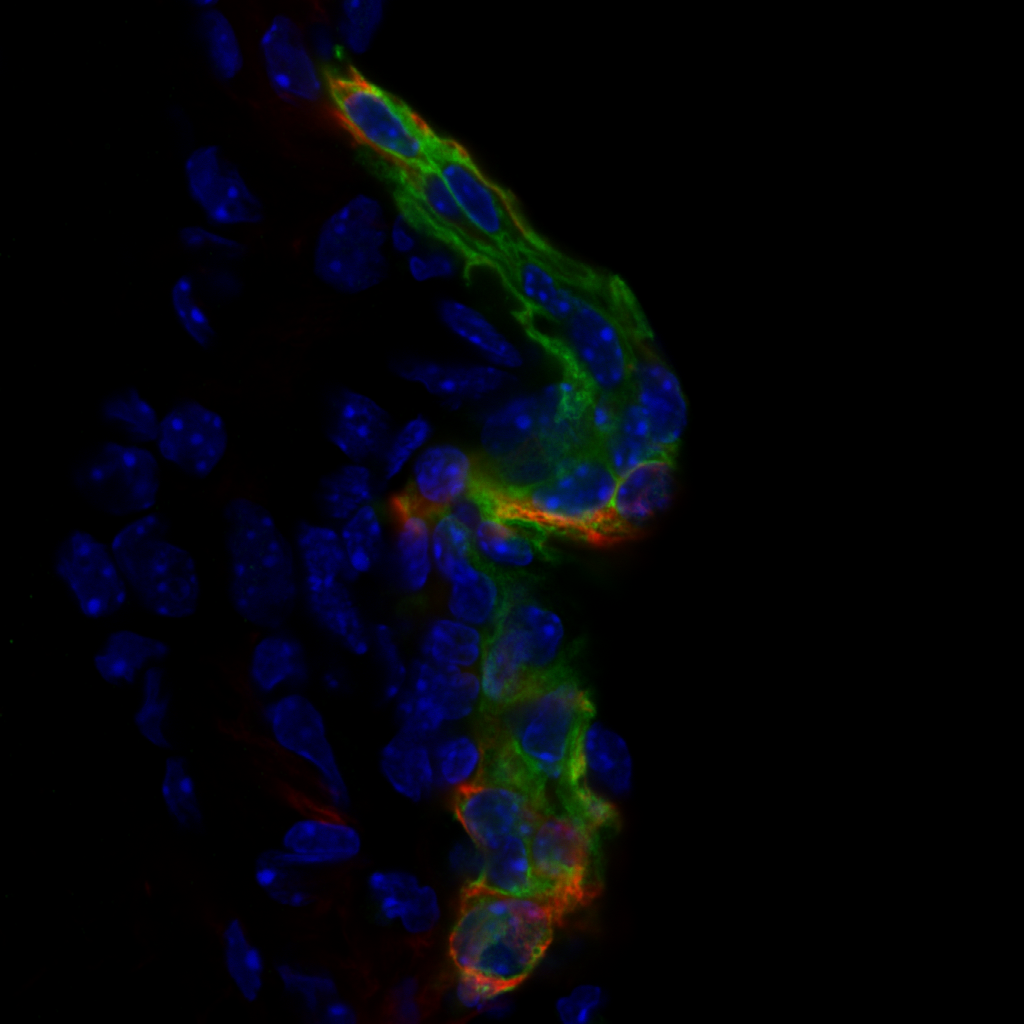

Supplement: Supplementary file 7 — Source data Fig. 5 [file 44321_2024_144_MOESM7_ESM.zip › Figure 5/5C/GFAP GFP 4_RGB.tif]

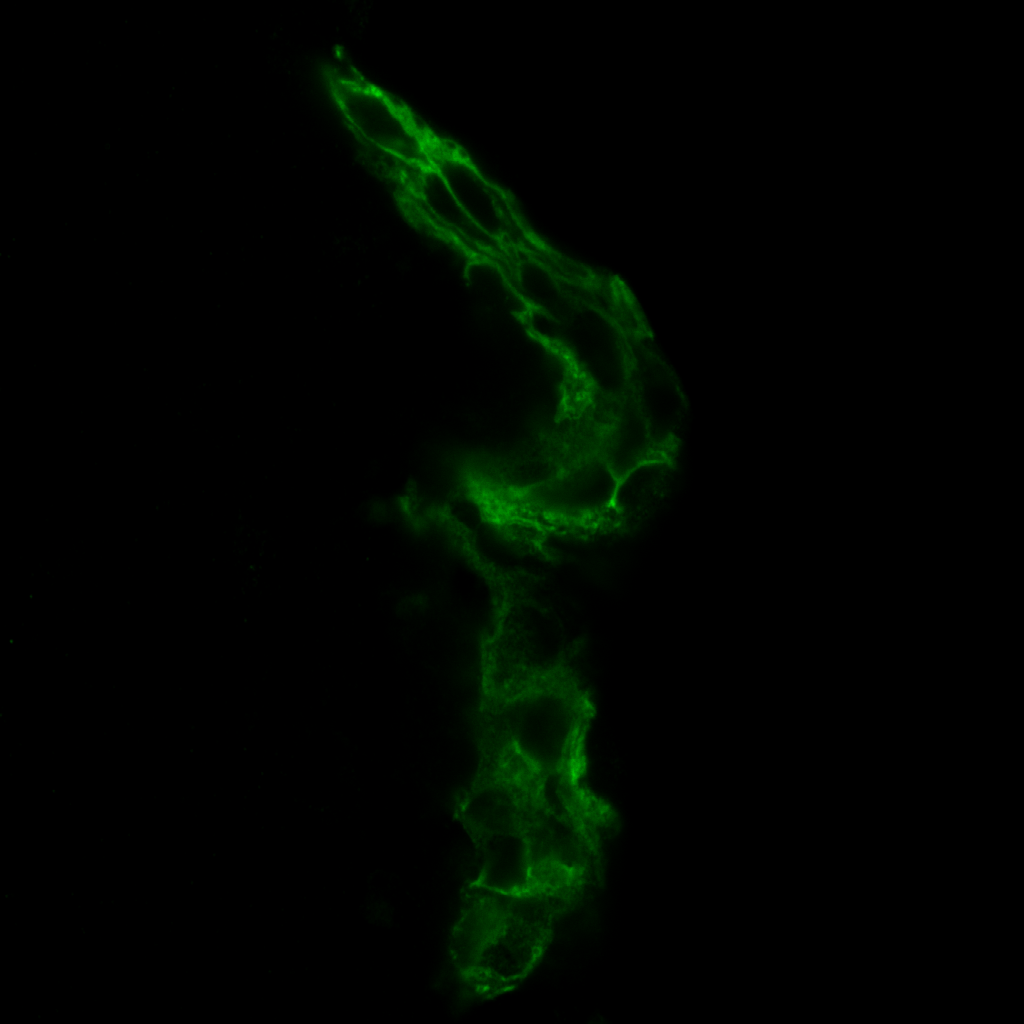

Supplement: Supplementary file 7 — Source data Fig. 5 [file 44321_2024_144_MOESM7_ESM.zip › Figure 5/5C/GFAP GFP 4_RGB_EGFP.tif]

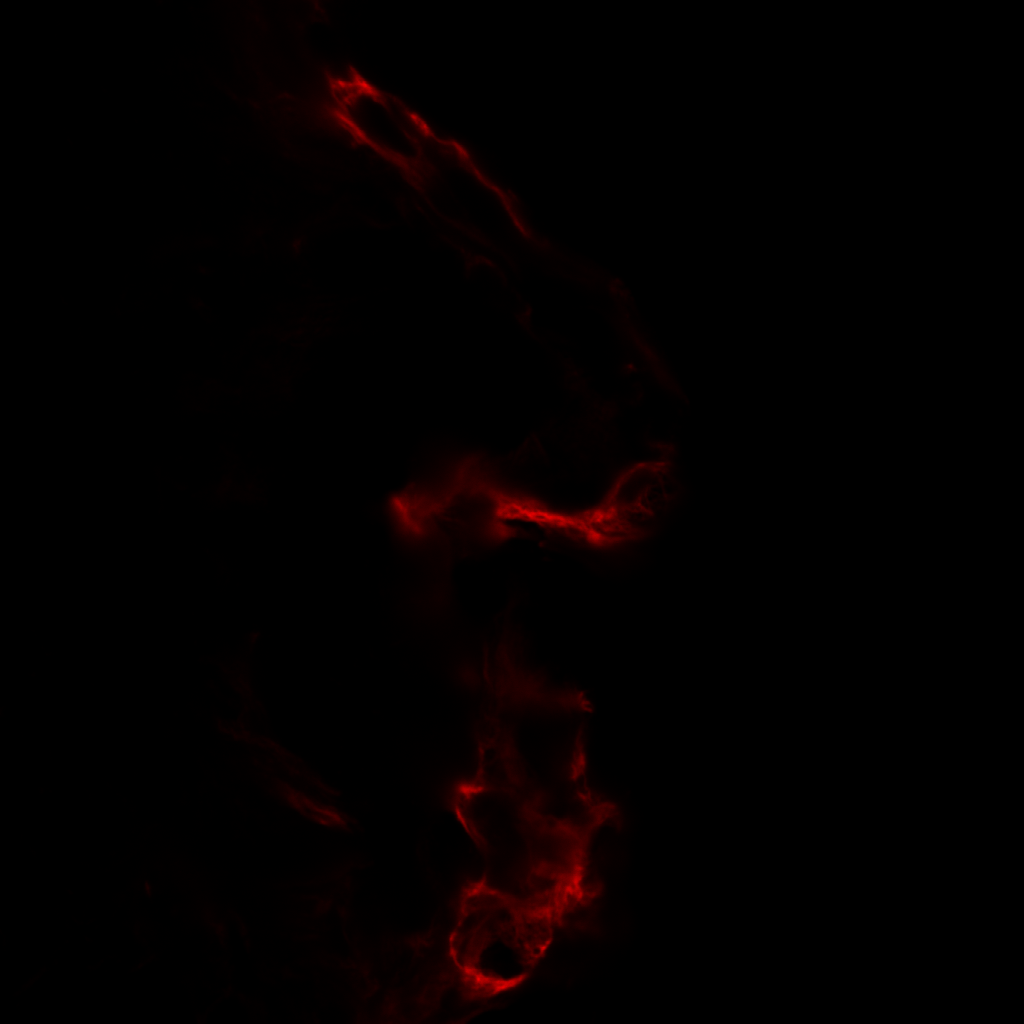

Supplement: Supplementary file 7 — Source data Fig. 5 [file 44321_2024_144_MOESM7_ESM.zip › Figure 5/5C/GFAP GFP 4_RGB_TRITC.tif]

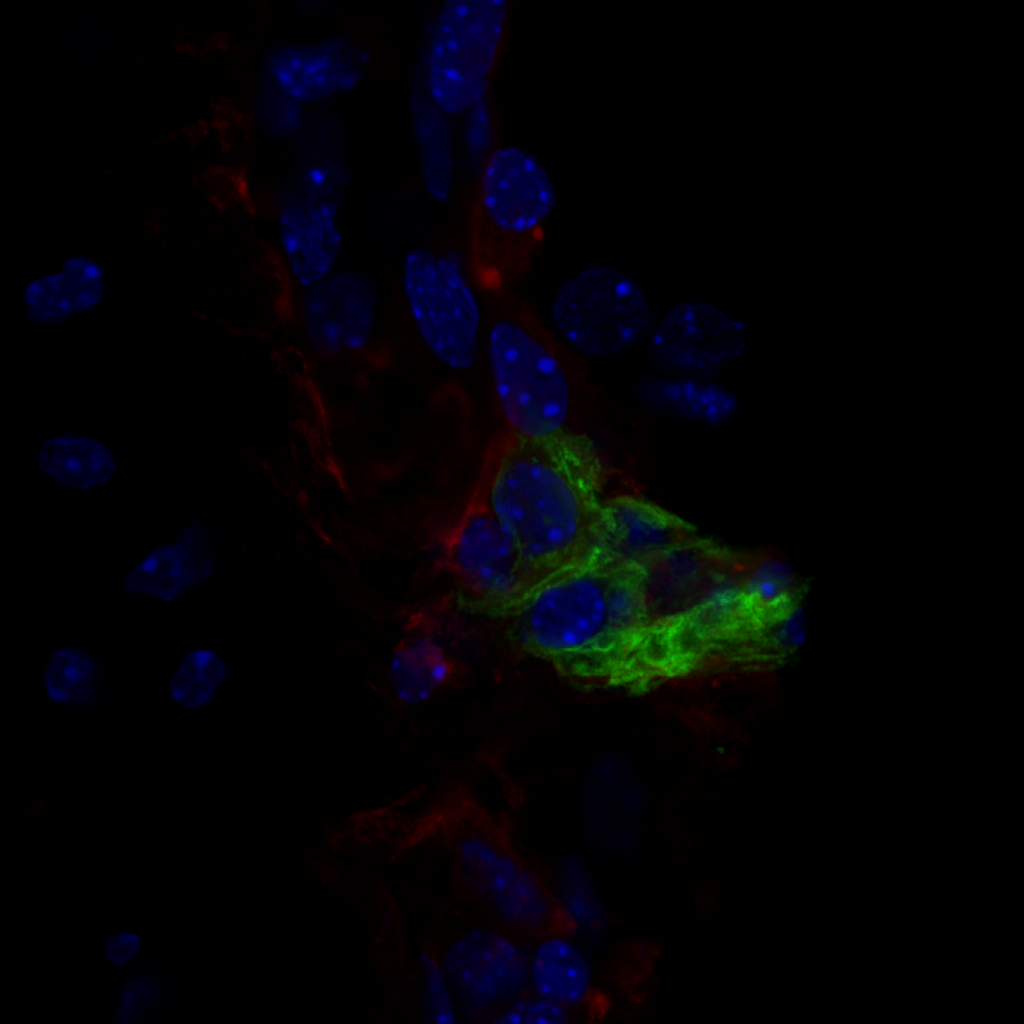

Supplement: Supplementary file 7 — Source data Fig. 5 [file 44321_2024_144_MOESM7_ESM.zip › Figure 5/5C/Nestina GFP 1_RGB.tif]

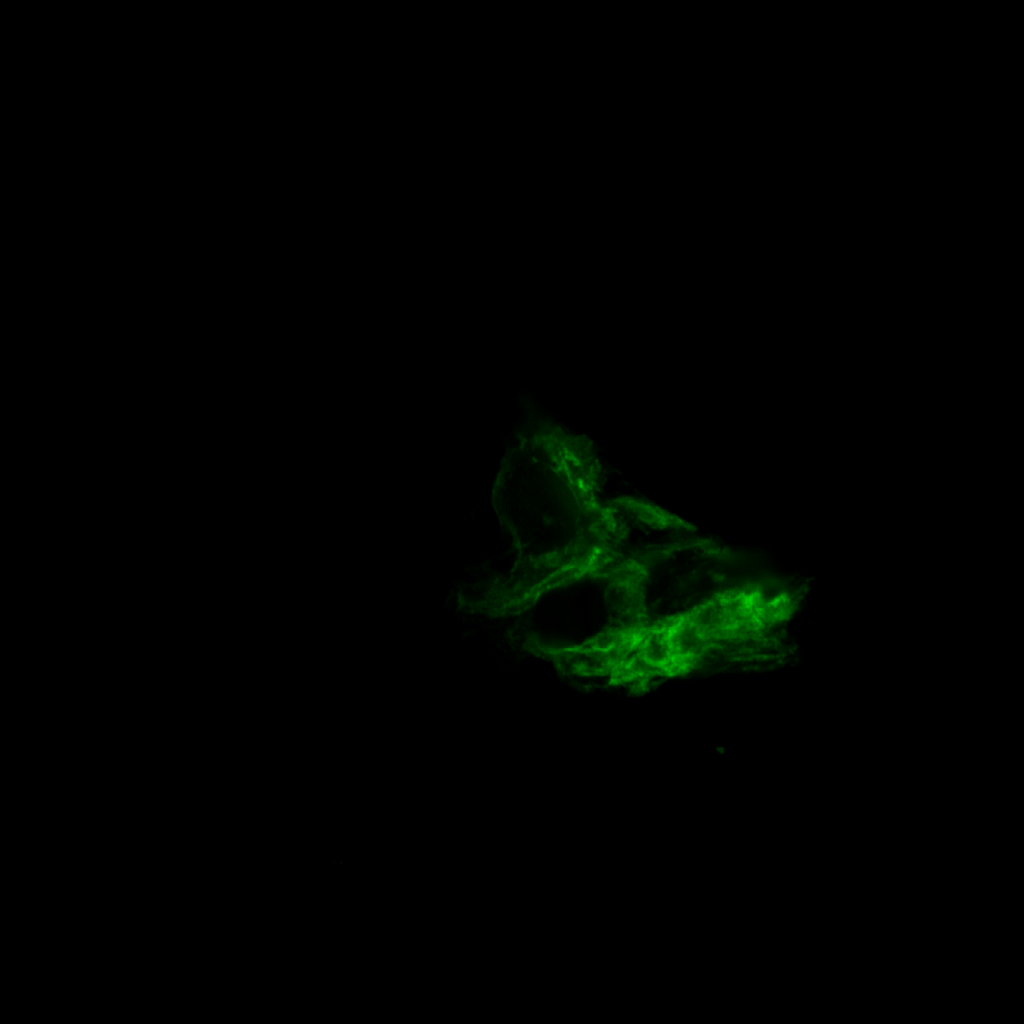

Supplement: Supplementary file 7 — Source data Fig. 5 [file 44321_2024_144_MOESM7_ESM.zip › Figure 5/5C/Nestina GFP 1_RGB_EGFP.tif]

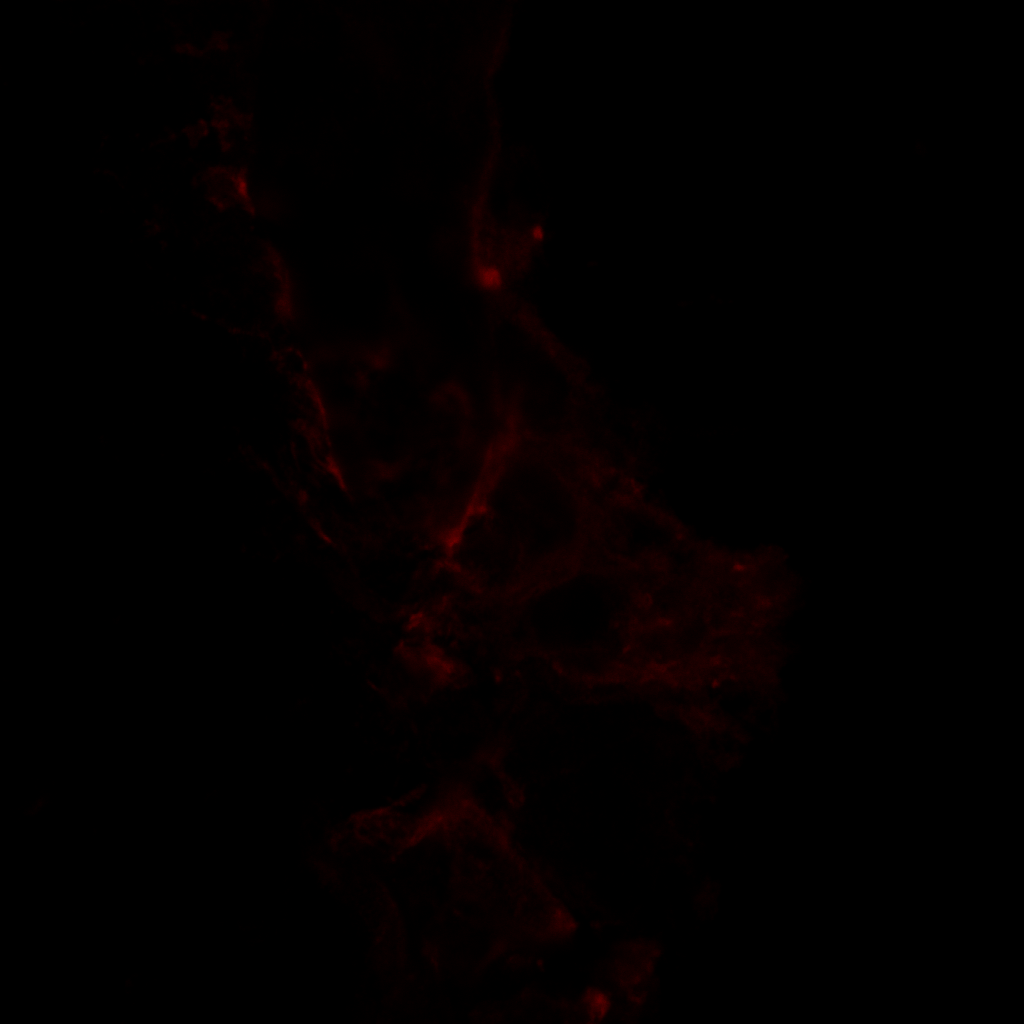

Supplement: Supplementary file 7 — Source data Fig. 5 [file 44321_2024_144_MOESM7_ESM.zip › Figure 5/5C/Nestina GFP 1_RGB_TRITC.tif]

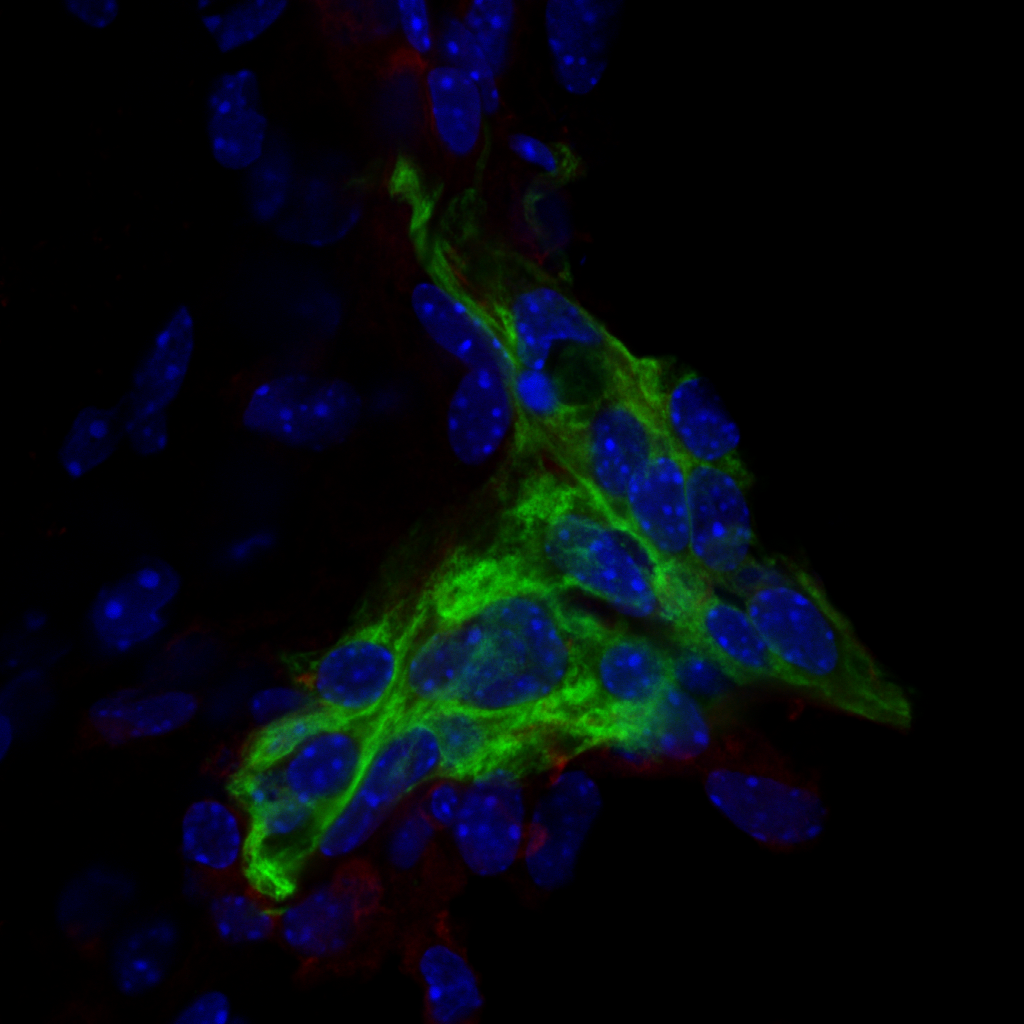

Supplement: Supplementary file 7 — Source data Fig. 5 [file 44321_2024_144_MOESM7_ESM.zip › Figure 5/5C/NeuN GFP 3_RGB.tif]

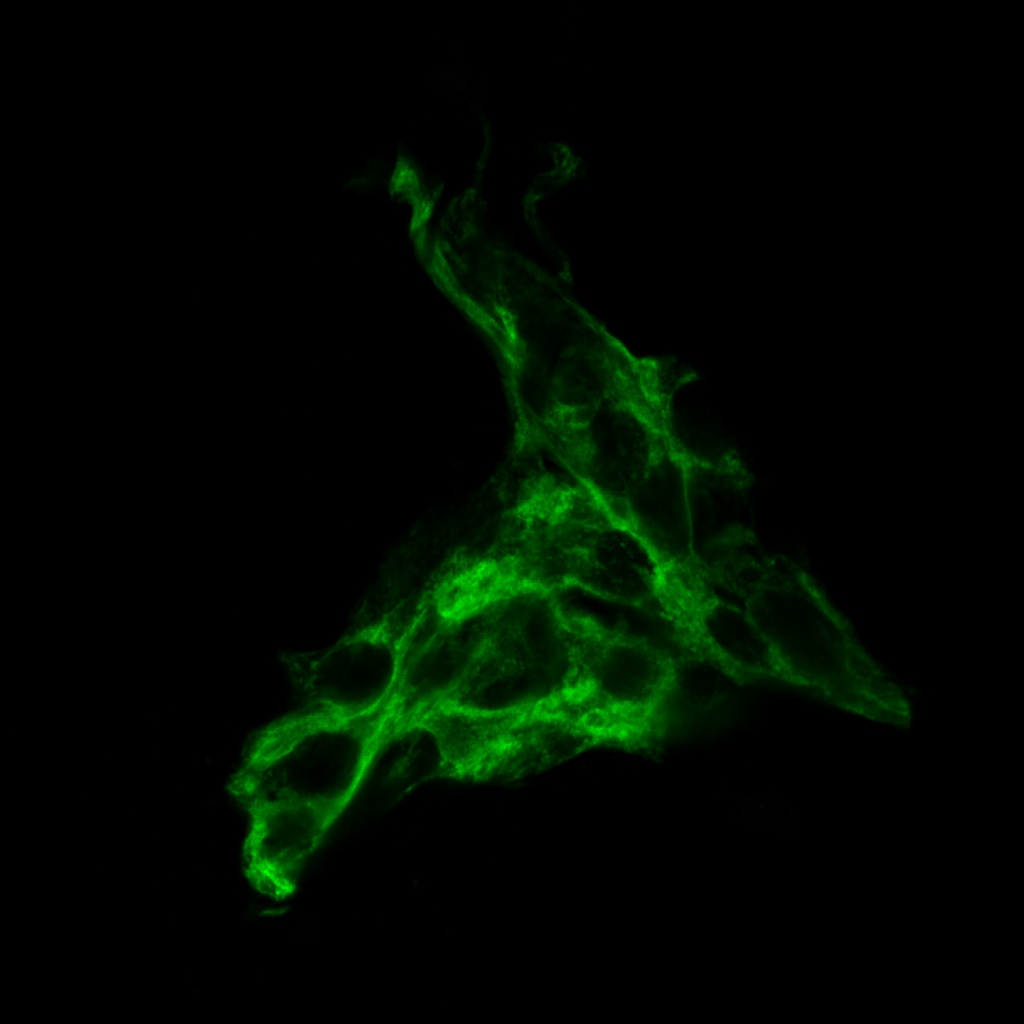

Supplement: Supplementary file 7 — Source data Fig. 5 [file 44321_2024_144_MOESM7_ESM.zip › Figure 5/5C/NeuN GFP 3_RGB_EGFP.tif]

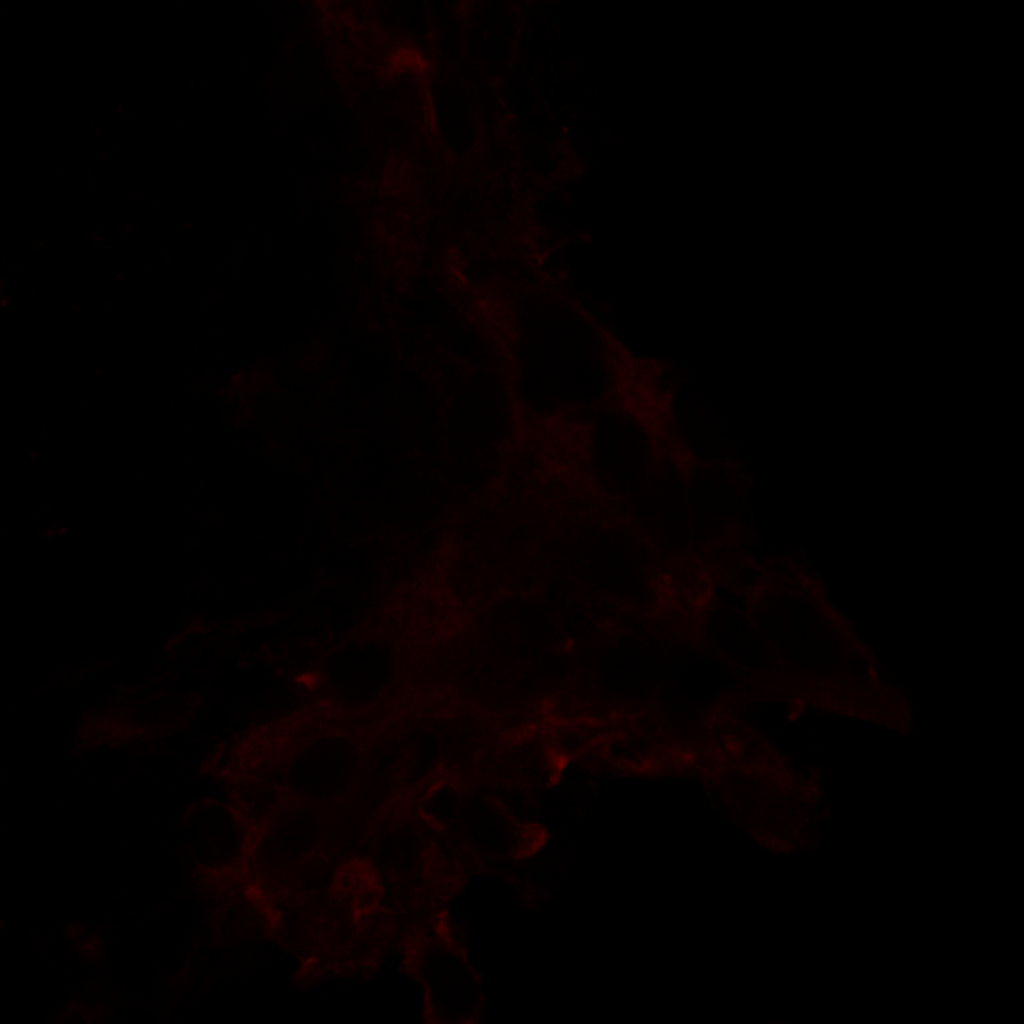

Supplement: Supplementary file 7 — Source data Fig. 5 [file 44321_2024_144_MOESM7_ESM.zip › Figure 5/5C/NeuN GFP 3_RGB_TRITC.tif]

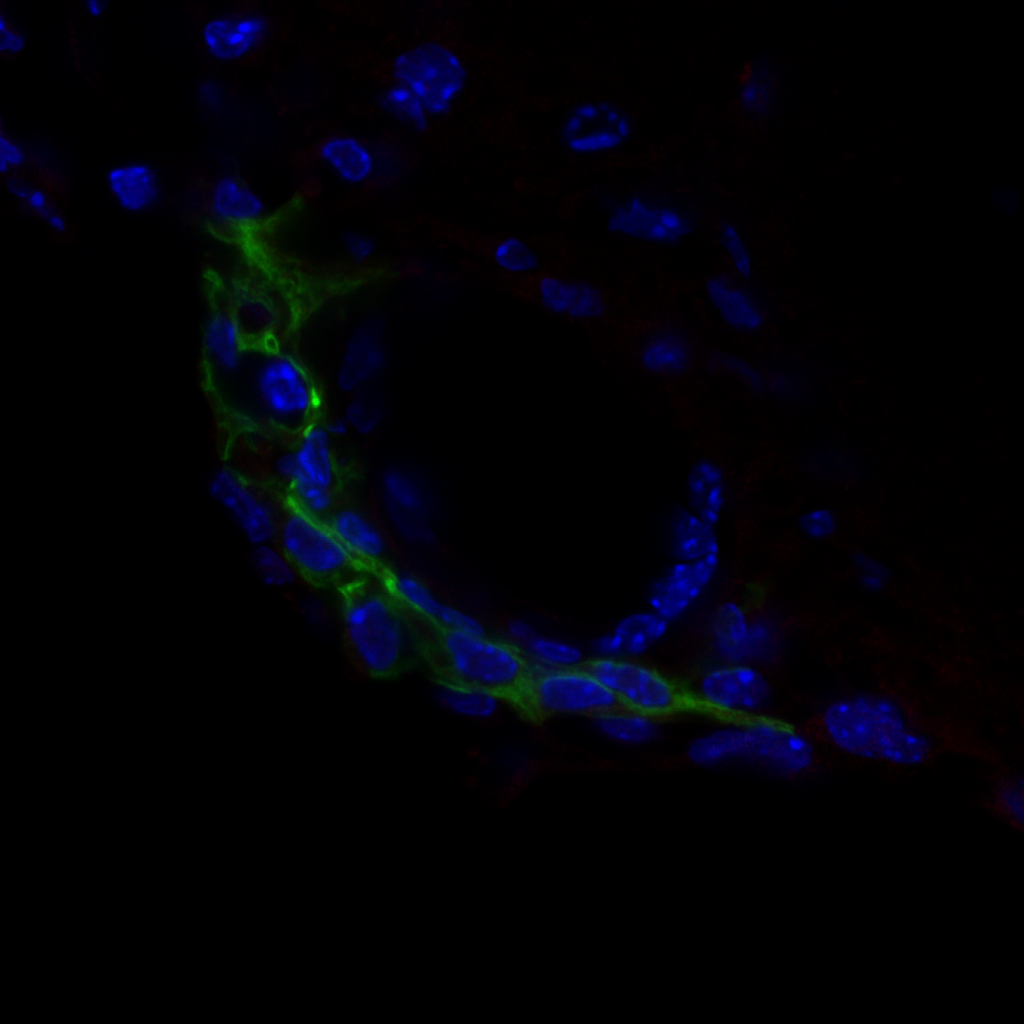

Supplement: Supplementary file 7 — Source data Fig. 5 [file 44321_2024_144_MOESM7_ESM.zip › Figure 5/5C/olig2 GFP 1_RGB.tif]

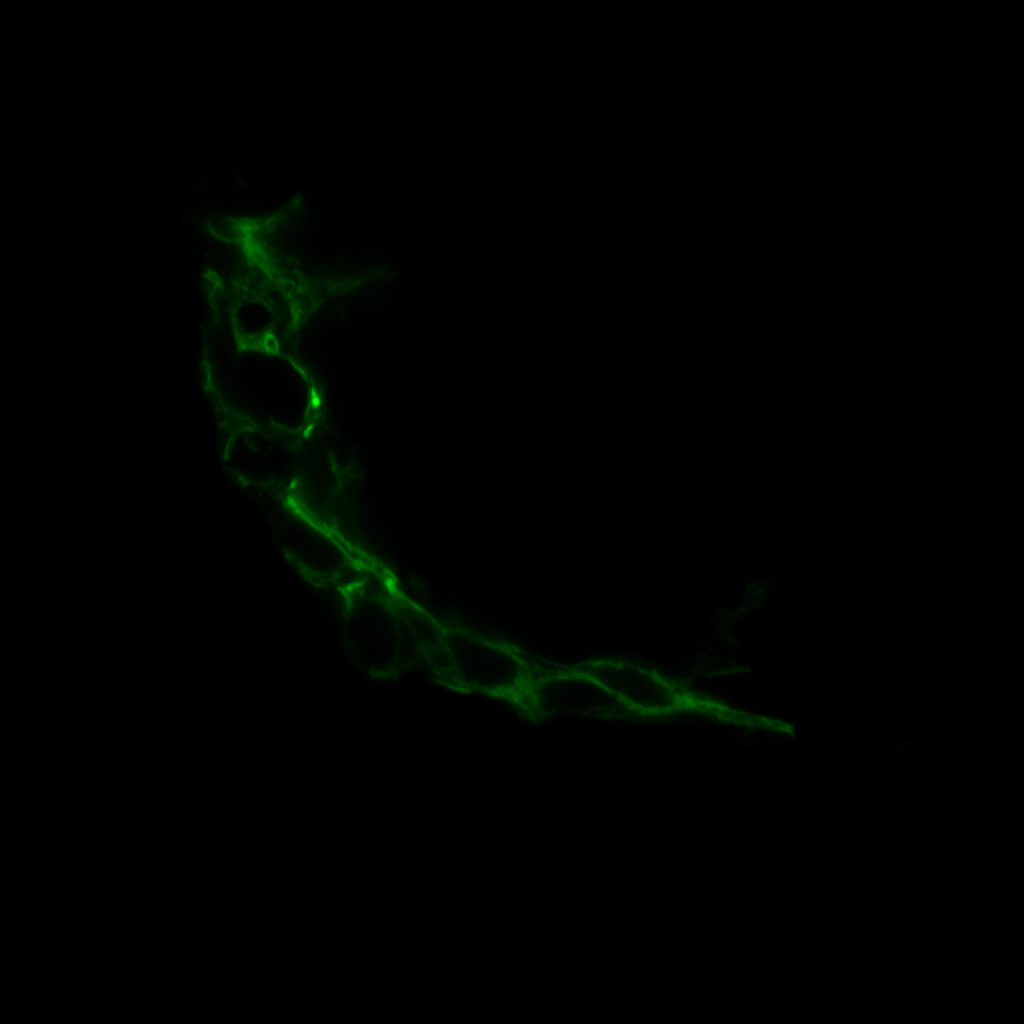

Supplement: Supplementary file 7 — Source data Fig. 5 [file 44321_2024_144_MOESM7_ESM.zip › Figure 5/5C/olig2 GFP 1_RGB_EGFP.tif]

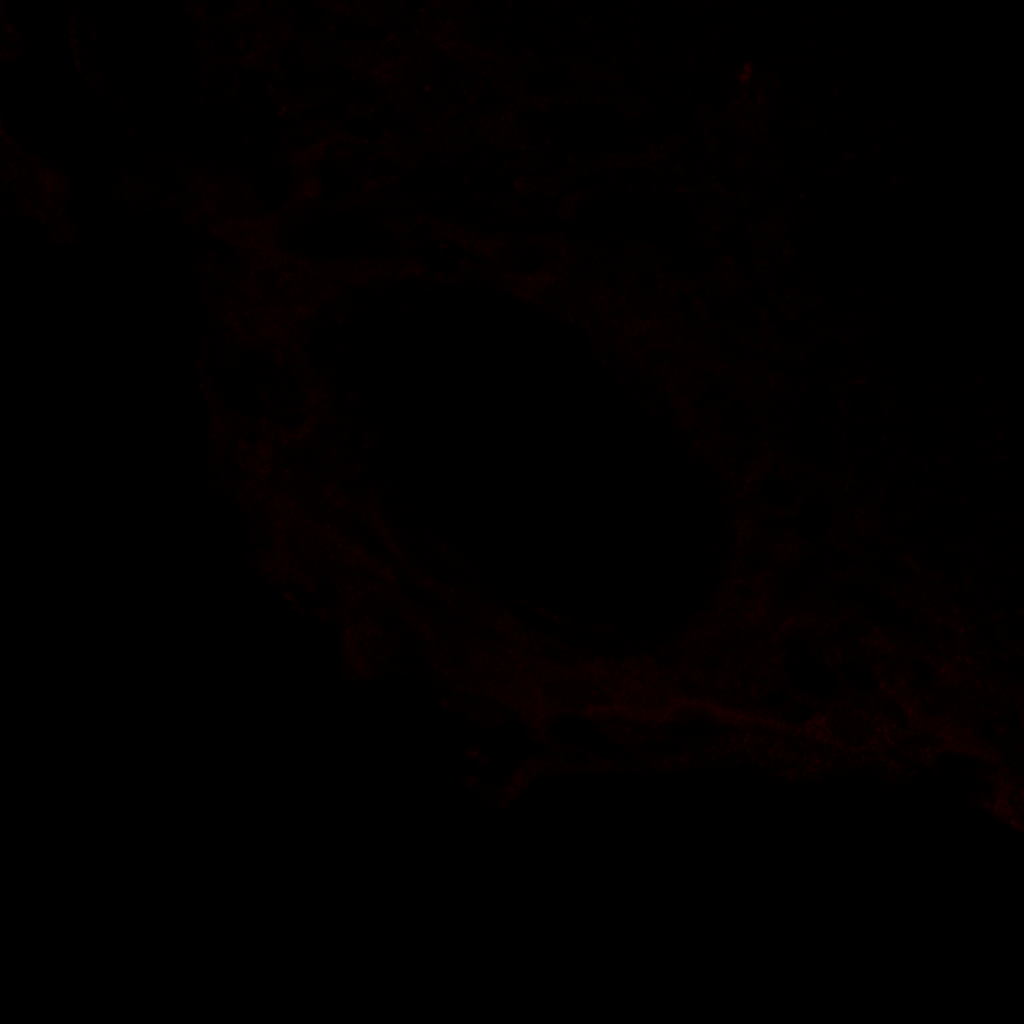

Supplement: Supplementary file 7 — Source data Fig. 5 [file 44321_2024_144_MOESM7_ESM.zip › Figure 5/5C/olig2 GFP 1_RGB_TRITC.tif]

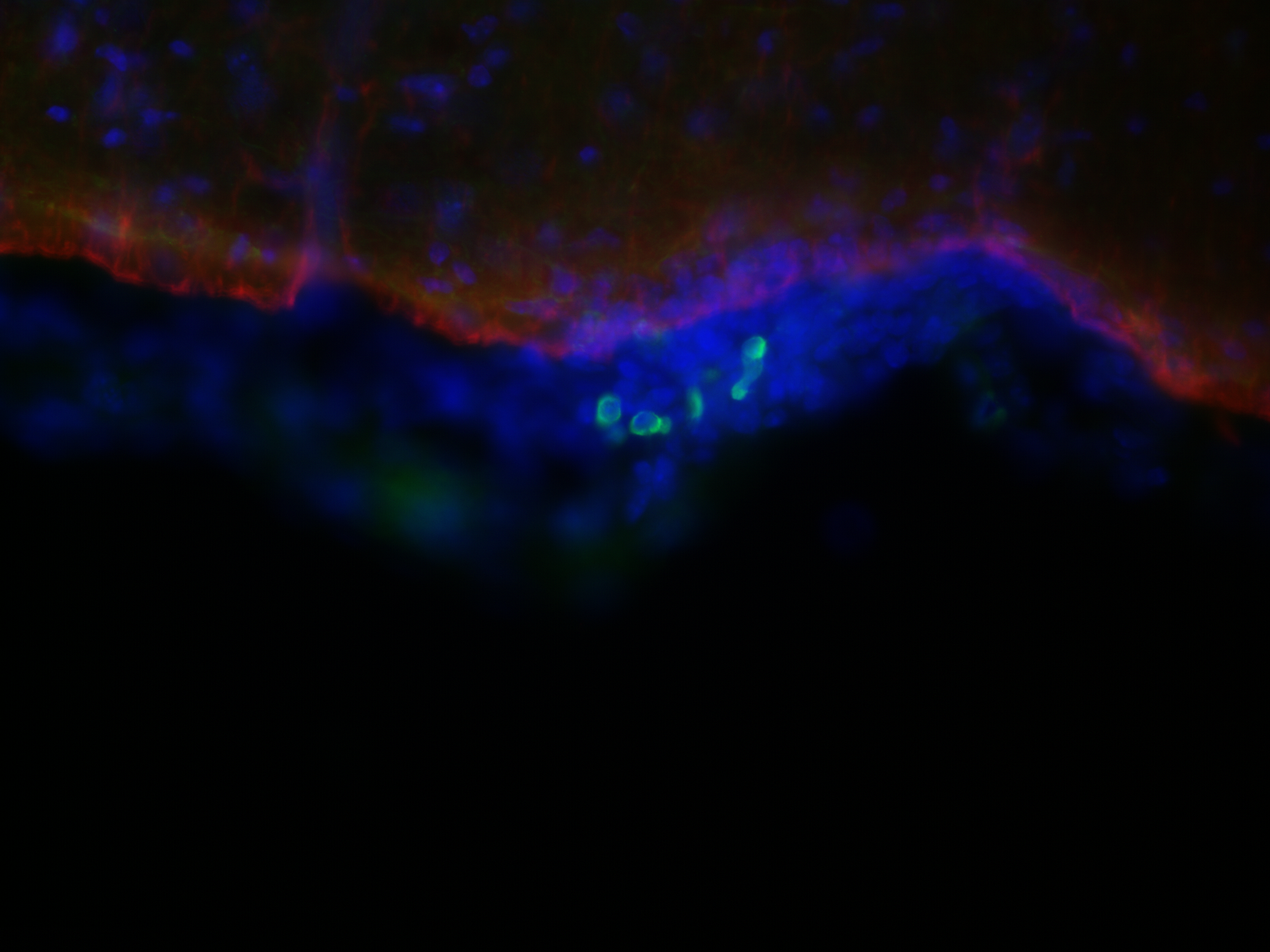

Supplement: Supplementary file 8 — Source data Fig. 6 [file 44321_2024_144_MOESM8_ESM.zip › Figure 6/6A/1580_2_40X.tif]

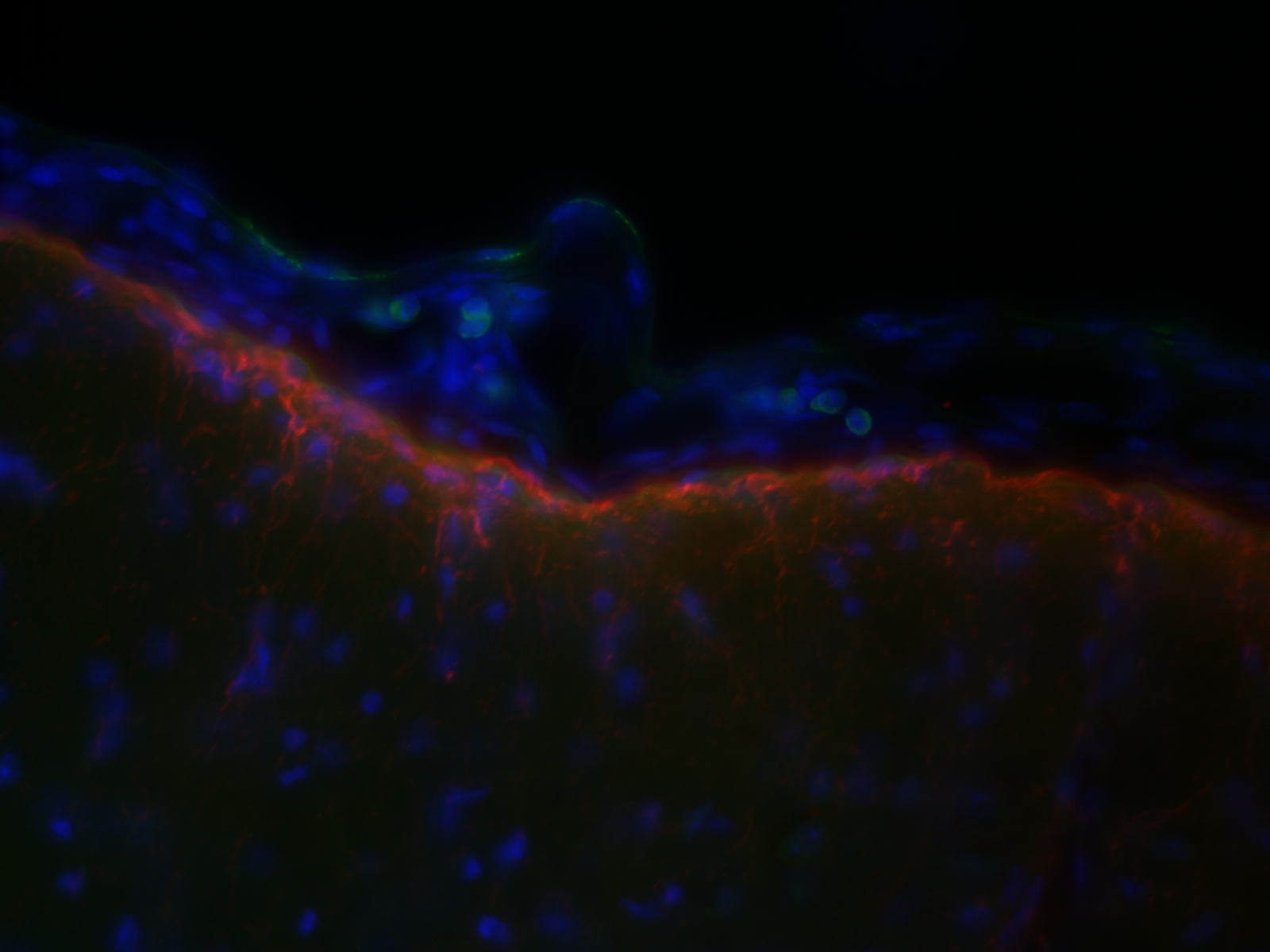

Supplement: Supplementary file 8 — Source data Fig. 6 [file 44321_2024_144_MOESM8_ESM.zip › Figure 6/6A/1580_4_40X.tif]

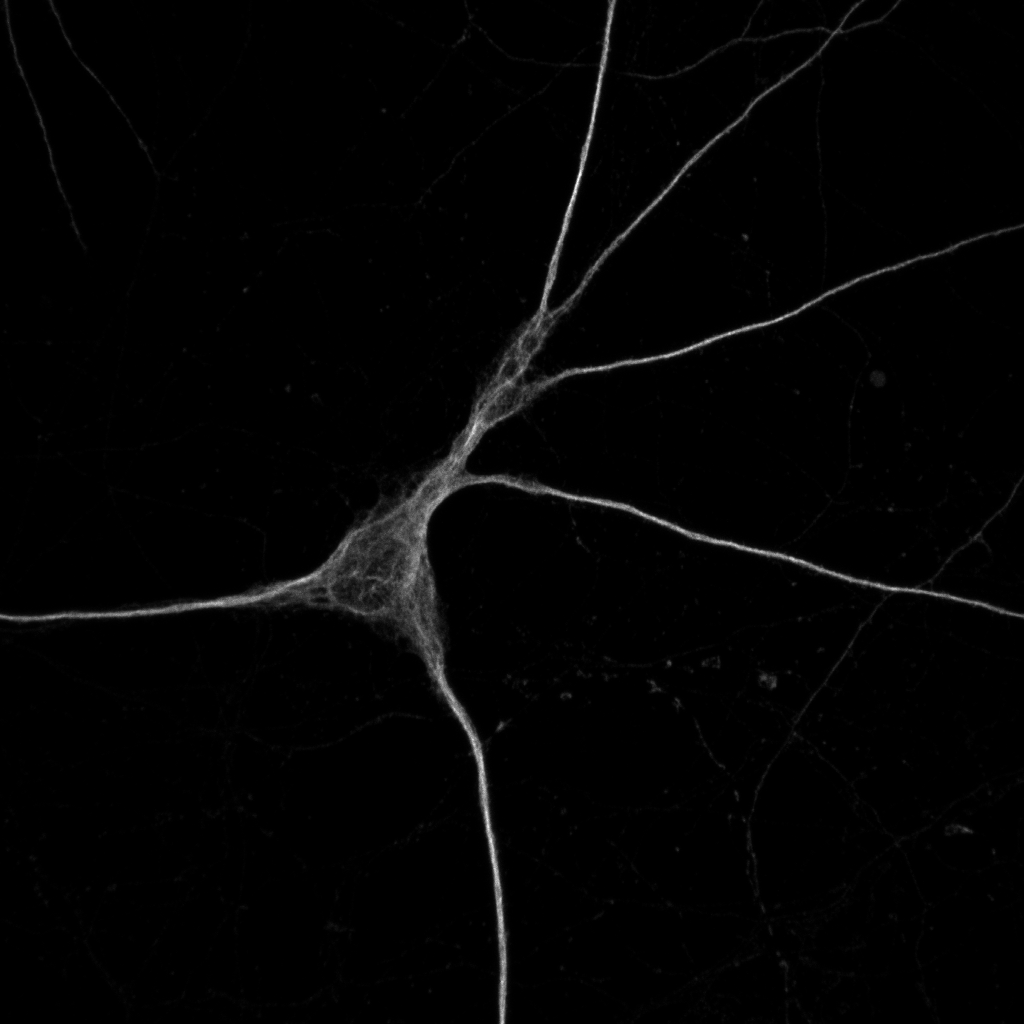

Supplement: Supplementary file 10 — Source data Fig. 8 [file 44321_2024_144_MOESM10_ESM.zip › Figure 8/8A/KO_IFNg_100/MAP2_KO_IFNg_100ng.tif]

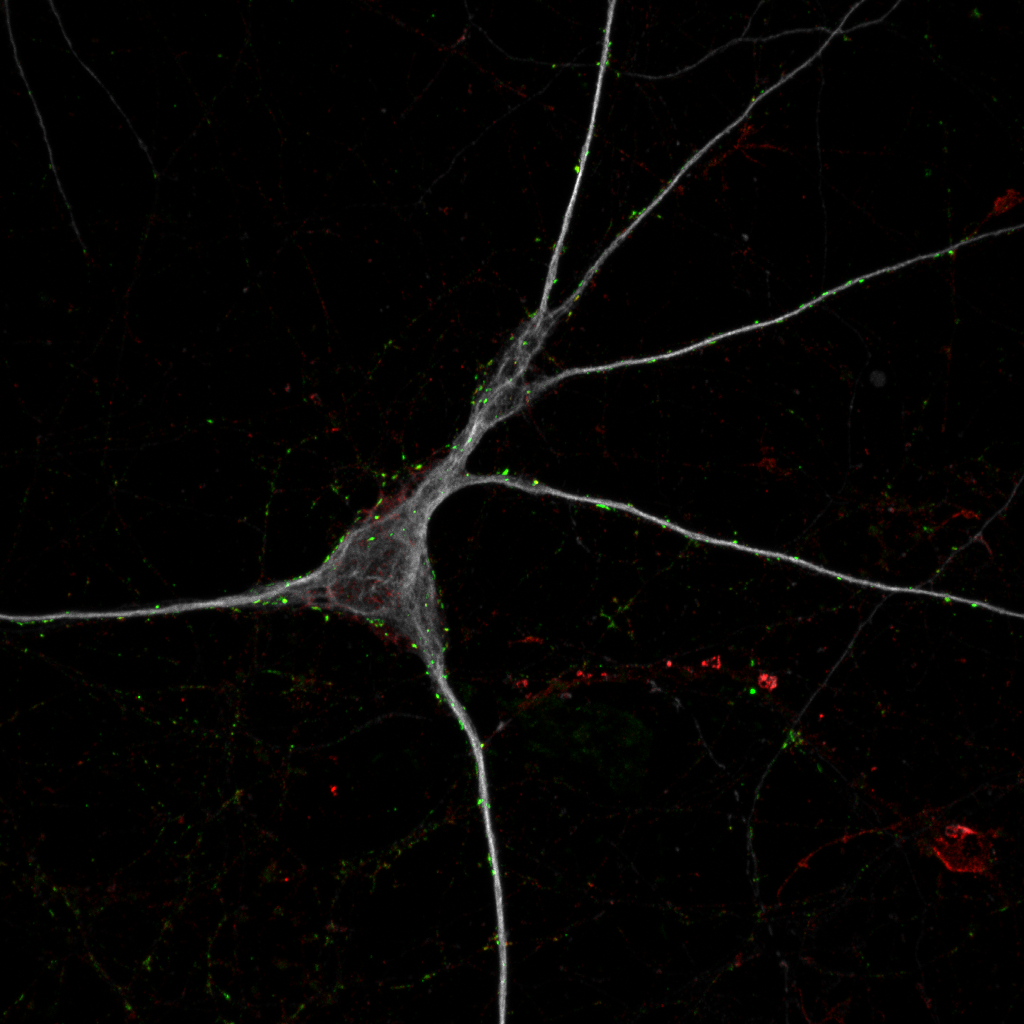

Supplement: Supplementary file 10 — Source data Fig. 8 [file 44321_2024_144_MOESM10_ESM.zip › Figure 8/8A/KO_IFNg_100/MERGE_KO_IFNg_100ng.tif]

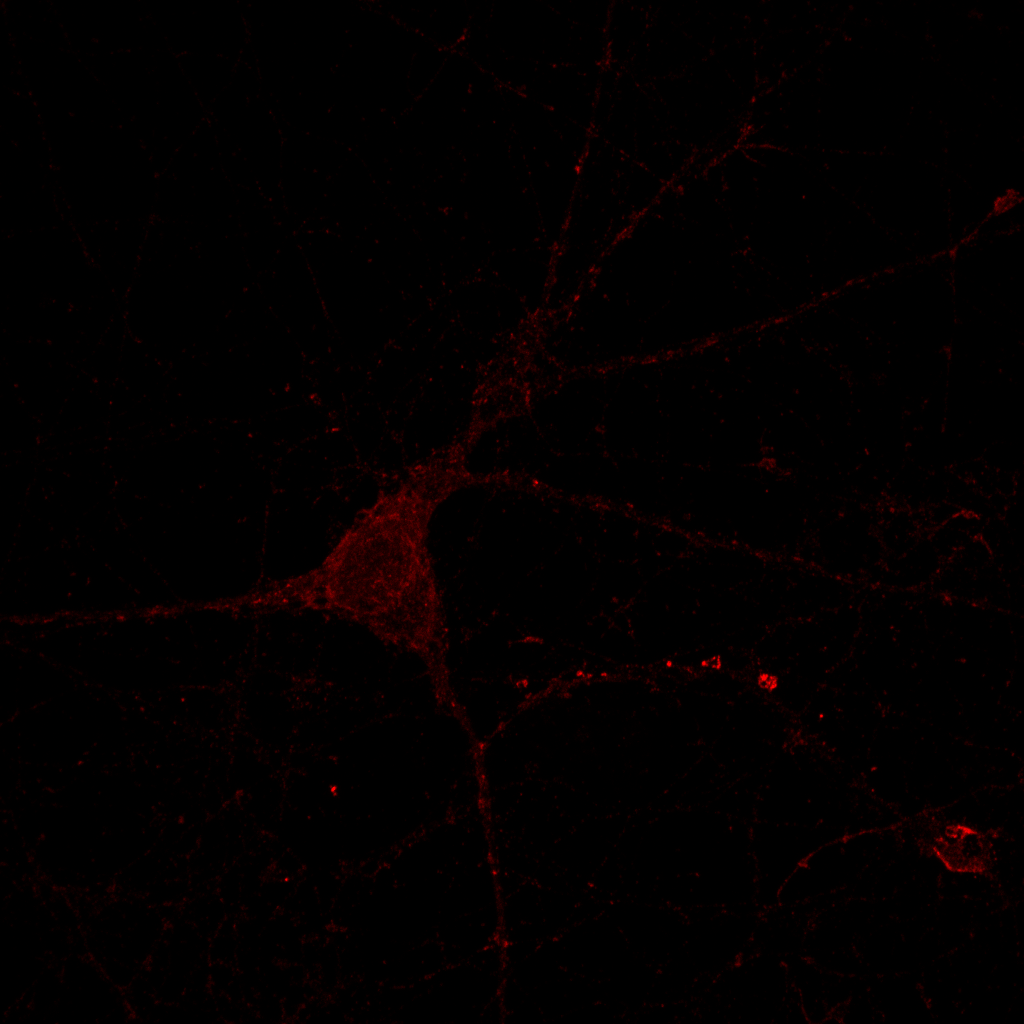

Supplement: Supplementary file 10 — Source data Fig. 8 [file 44321_2024_144_MOESM10_ESM.zip › Figure 8/8A/KO_IFNg_100/SHANK_KO_IFNg_100ng.tif]

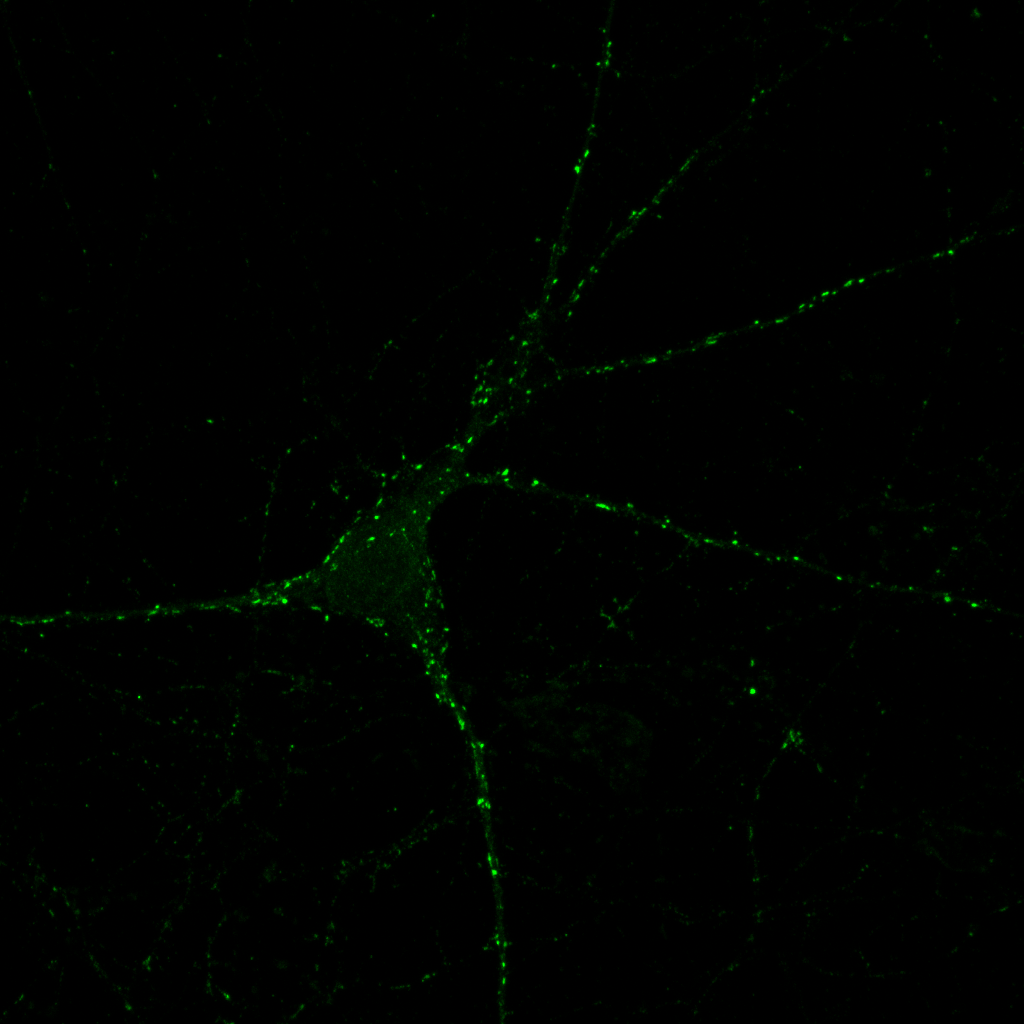

Supplement: Supplementary file 10 — Source data Fig. 8 [file 44321_2024_144_MOESM10_ESM.zip › Figure 8/8A/KO_IFNg_100/SYN_KO_IFNg_100ng.tif]

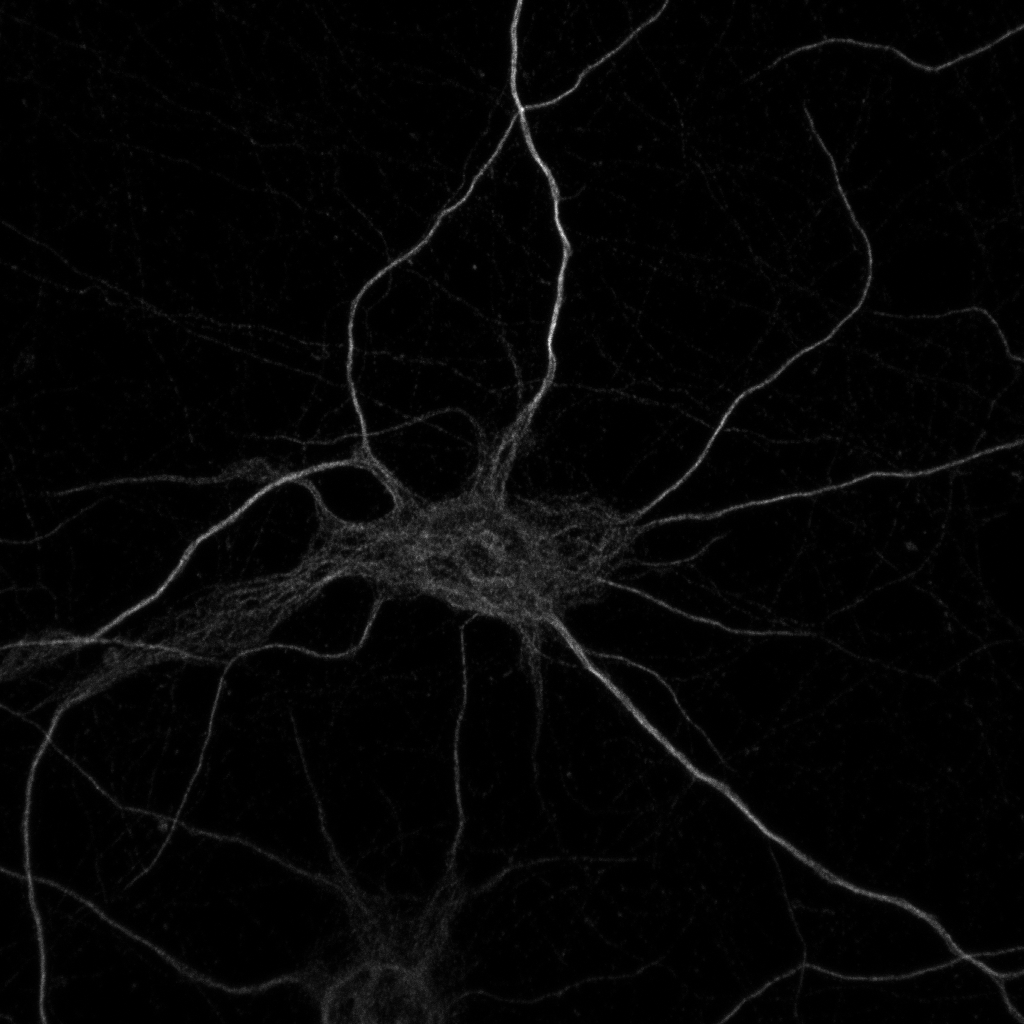

Supplement: Supplementary file 10 — Source data Fig. 8 [file 44321_2024_144_MOESM10_ESM.zip › Figure 8/8A/KO_IFNg_25/MAP2_KO_IFNg_25ng.tif]

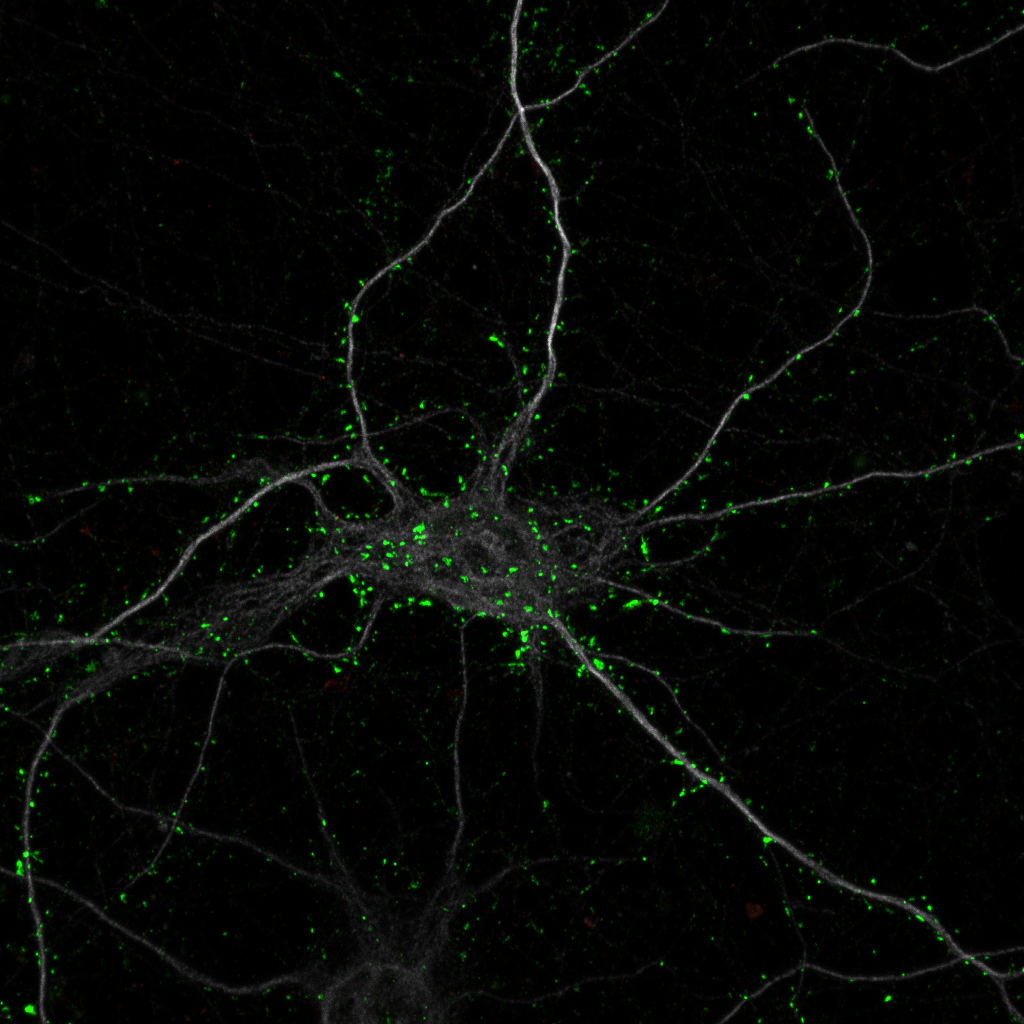

Supplement: Supplementary file 10 — Source data Fig. 8 [file 44321_2024_144_MOESM10_ESM.zip › Figure 8/8A/KO_IFNg_25/MERGE_KO_IFNg_25ng.tif]

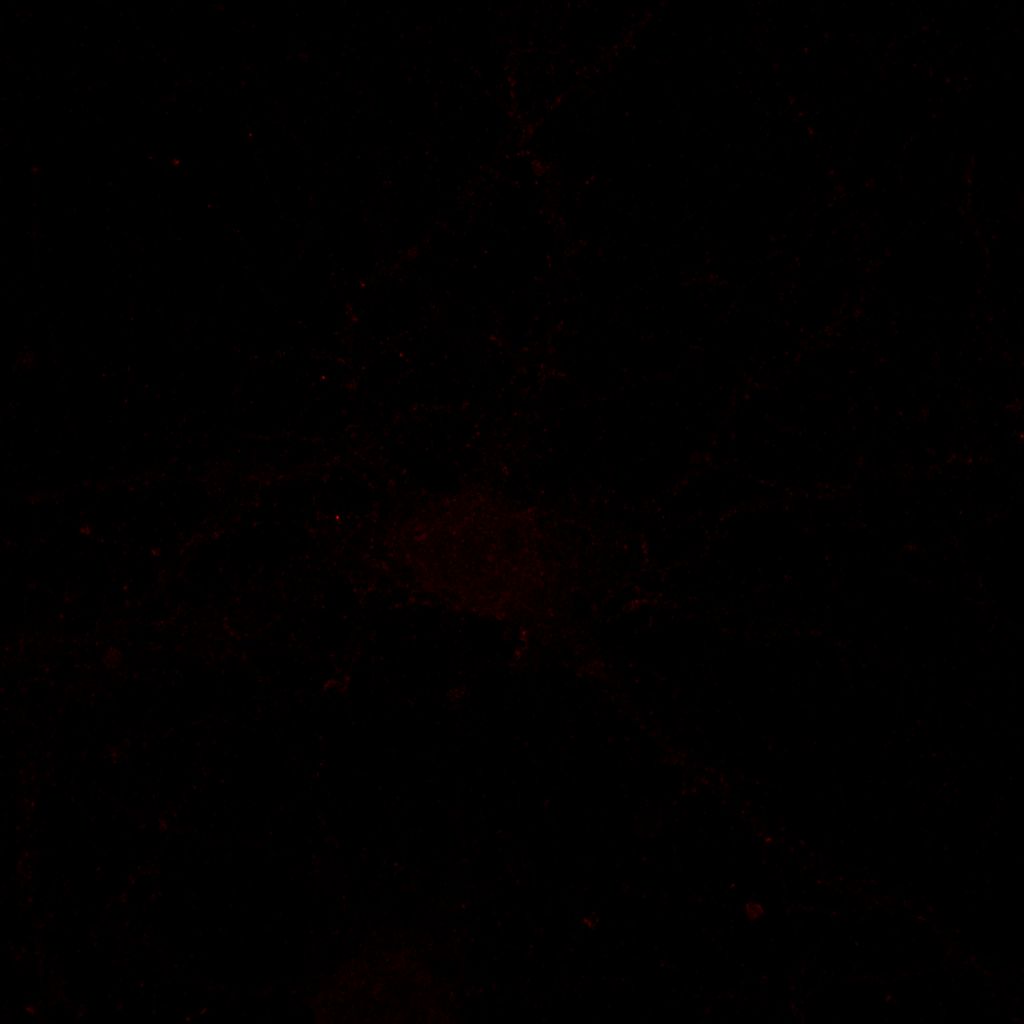

Supplement: Supplementary file 10 — Source data Fig. 8 [file 44321_2024_144_MOESM10_ESM.zip › Figure 8/8A/KO_IFNg_25/SHANK_KO_IFNg_25ng.tif]

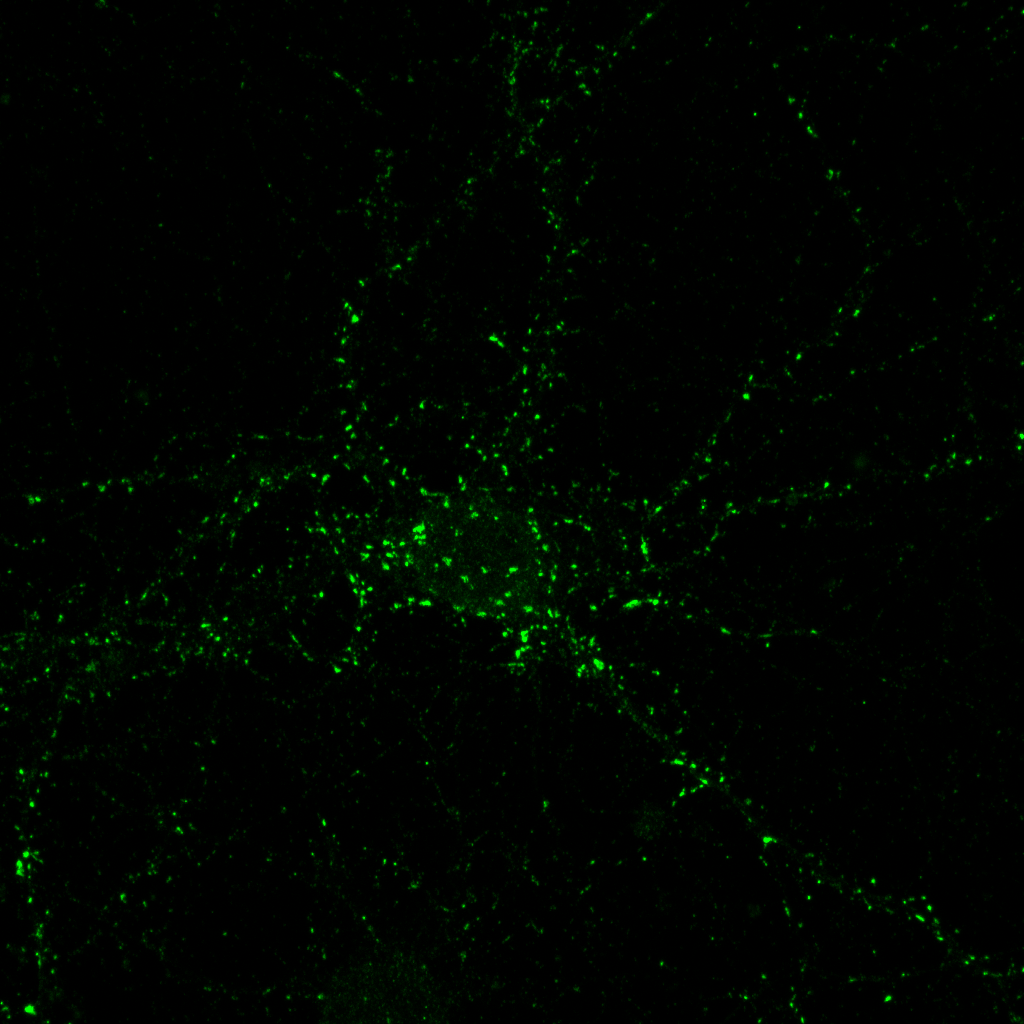

Supplement: Supplementary file 10 — Source data Fig. 8 [file 44321_2024_144_MOESM10_ESM.zip › Figure 8/8A/KO_IFNg_25/SYN_KO_IFNg_25ng.tif]

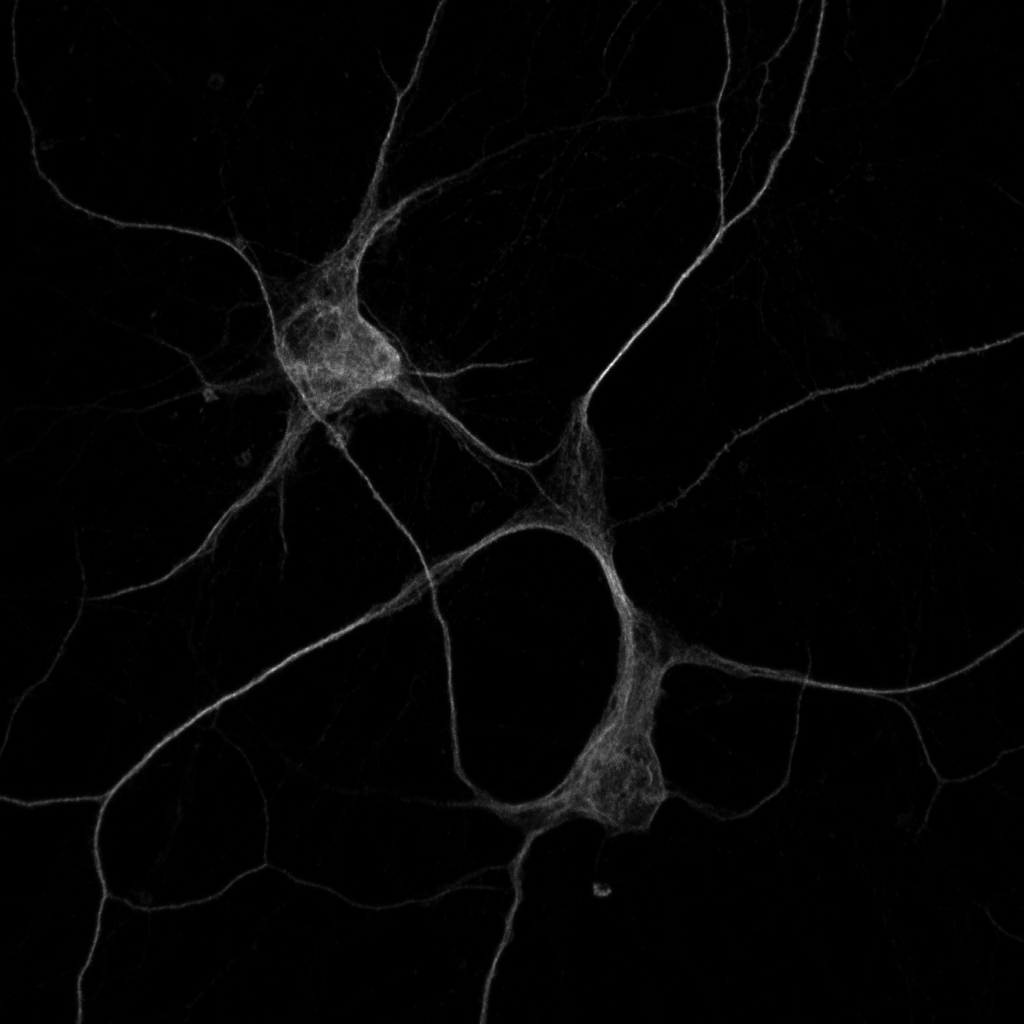

Supplement: Supplementary file 10 — Source data Fig. 8 [file 44321_2024_144_MOESM10_ESM.zip › Figure 8/8A/KO_UT/MAP2_KO_UT.tif]

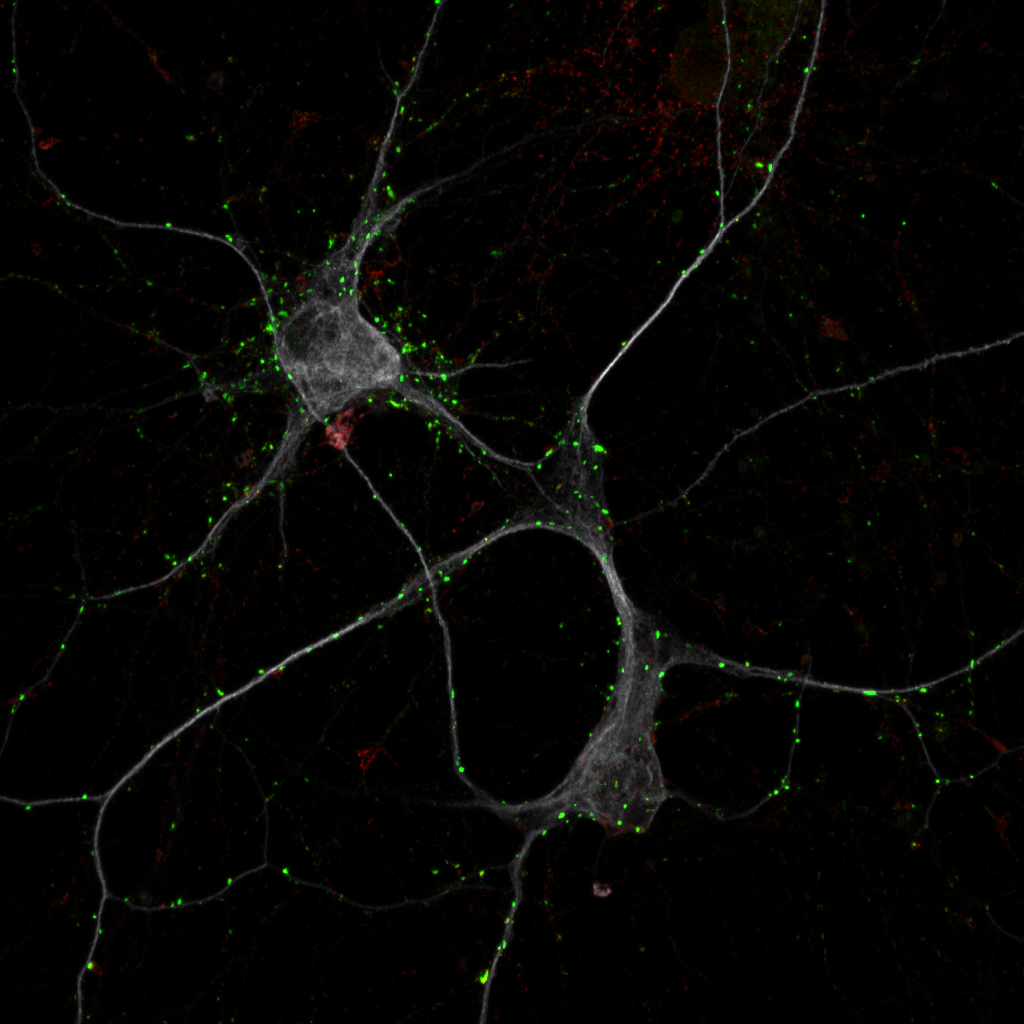

Supplement: Supplementary file 10 — Source data Fig. 8 [file 44321_2024_144_MOESM10_ESM.zip › Figure 8/8A/KO_UT/MERGE_KO_UT.tif]

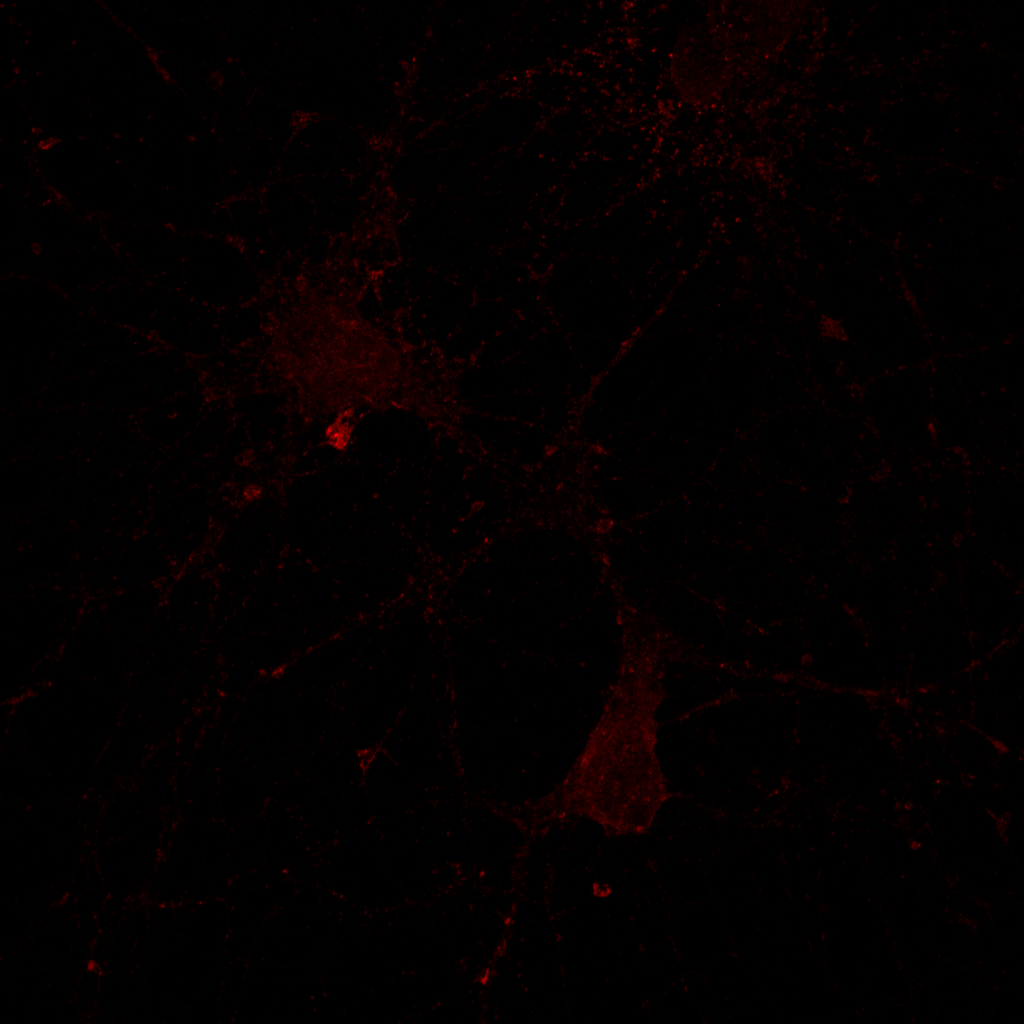

Supplement: Supplementary file 10 — Source data Fig. 8 [file 44321_2024_144_MOESM10_ESM.zip › Figure 8/8A/KO_UT/SHANK_KO_UT.tif]

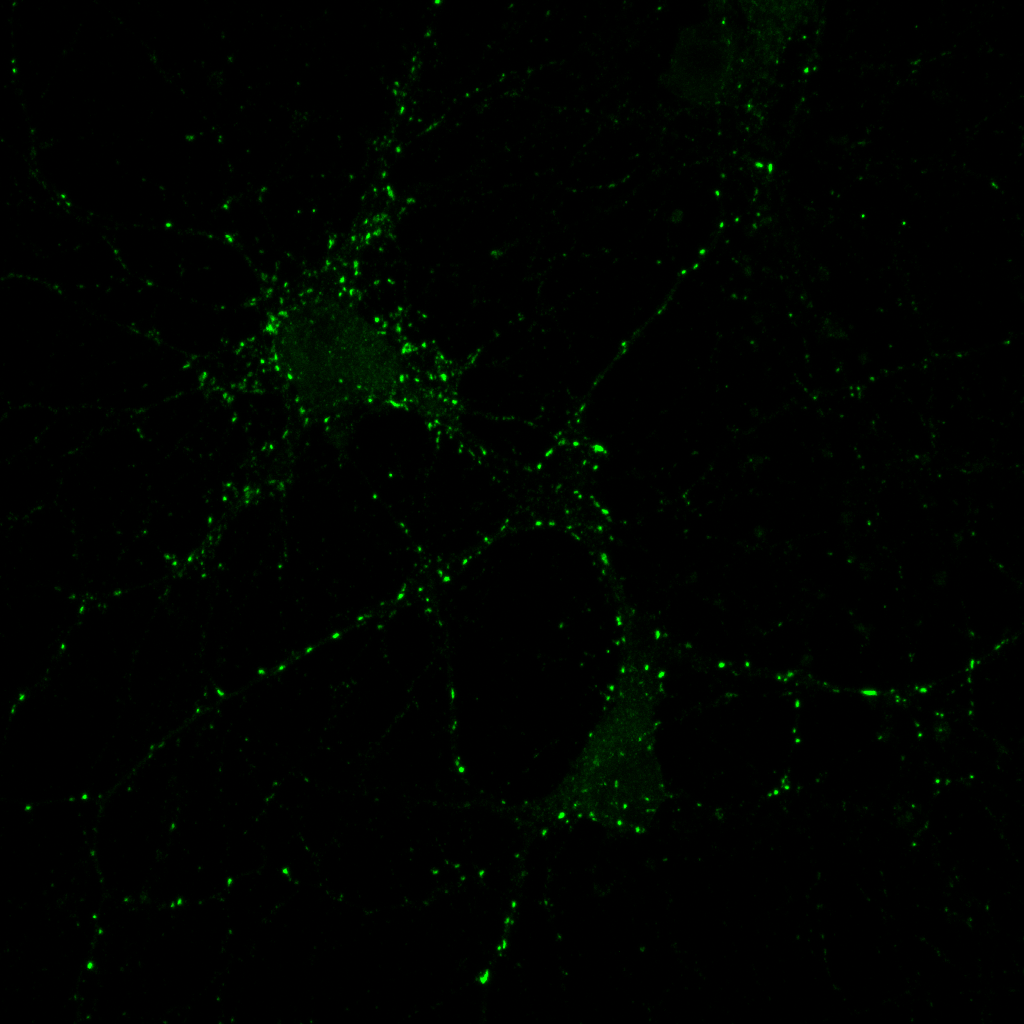

Supplement: Supplementary file 10 — Source data Fig. 8 [file 44321_2024_144_MOESM10_ESM.zip › Figure 8/8A/KO_UT/SYN_KO_UT.tif]

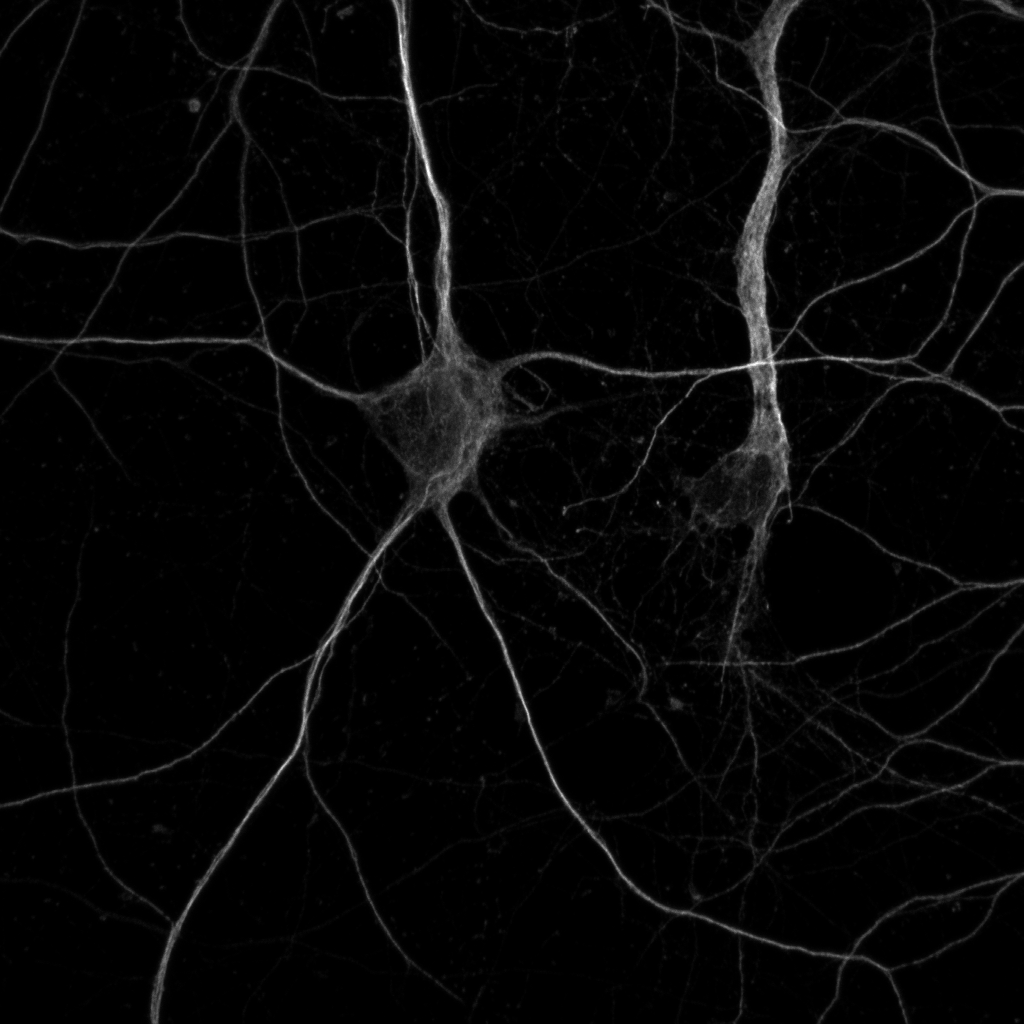

Supplement: Supplementary file 10 — Source data Fig. 8 [file 44321_2024_144_MOESM10_ESM.zip › Figure 8/8A/WT_UT/MAP2_WT_UT.tif]

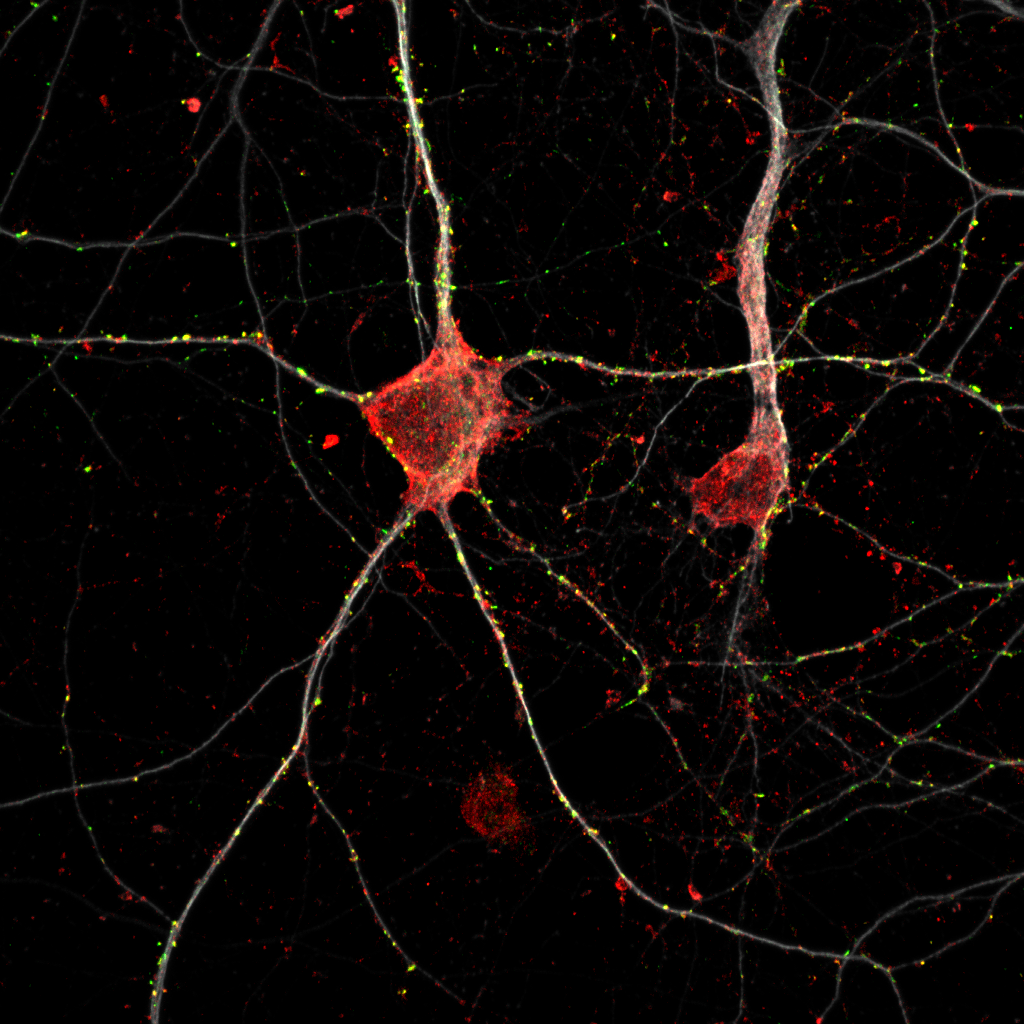

Supplement: Supplementary file 10 — Source data Fig. 8 [file 44321_2024_144_MOESM10_ESM.zip › Figure 8/8A/WT_UT/MERGE_WT_UT.tif]

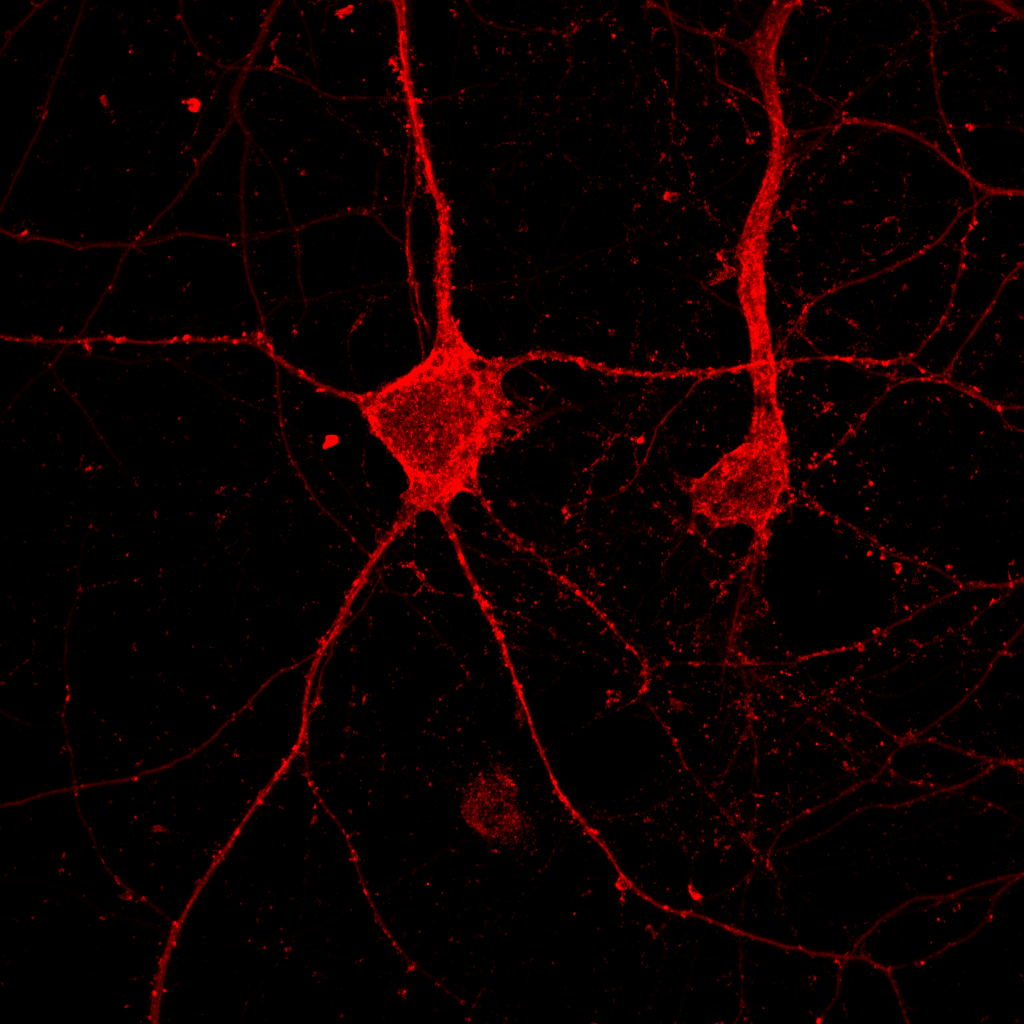

Supplement: Supplementary file 10 — Source data Fig. 8 [file 44321_2024_144_MOESM10_ESM.zip › Figure 8/8A/WT_UT/SHANK_WT_UT.tif]

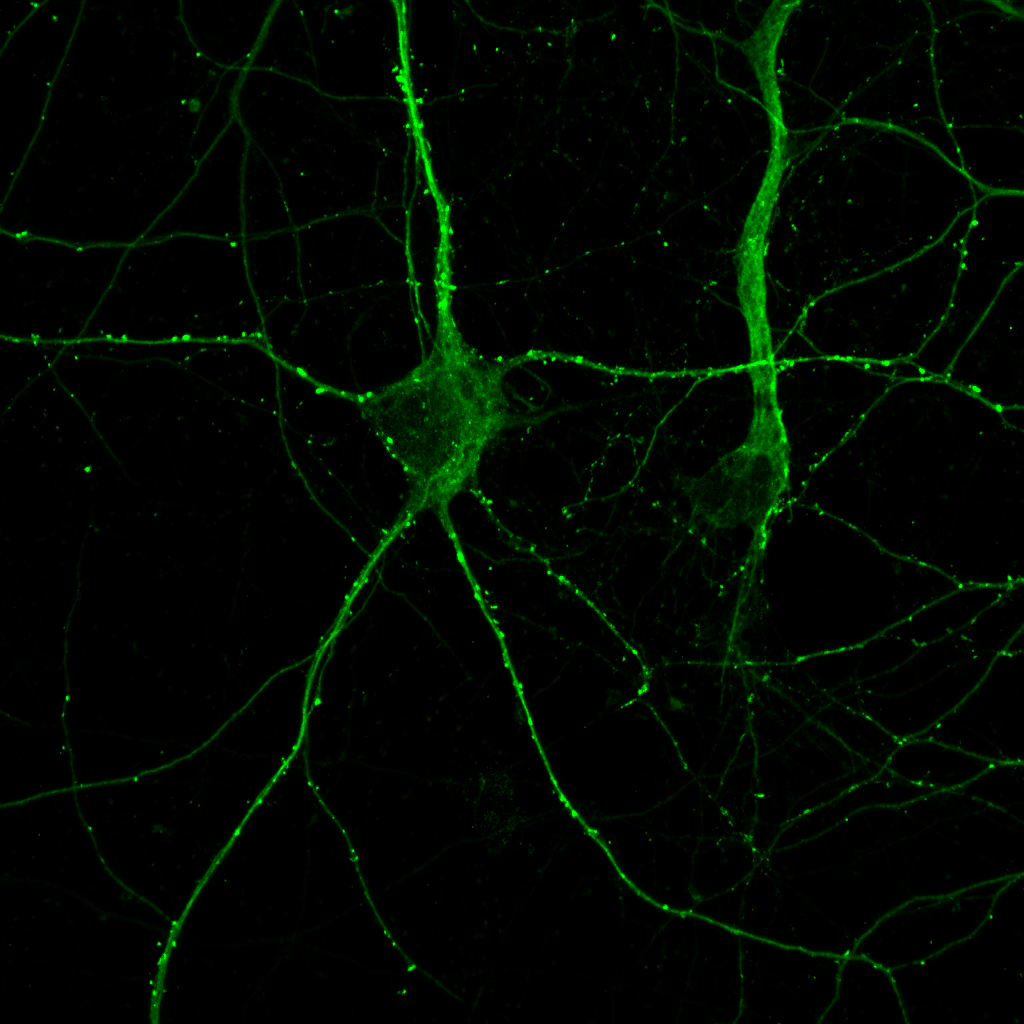

Supplement: Supplementary file 10 — Source data Fig. 8 [file 44321_2024_144_MOESM10_ESM.zip › Figure 8/8A/WT_UT/SYN_WT_UT.tif]

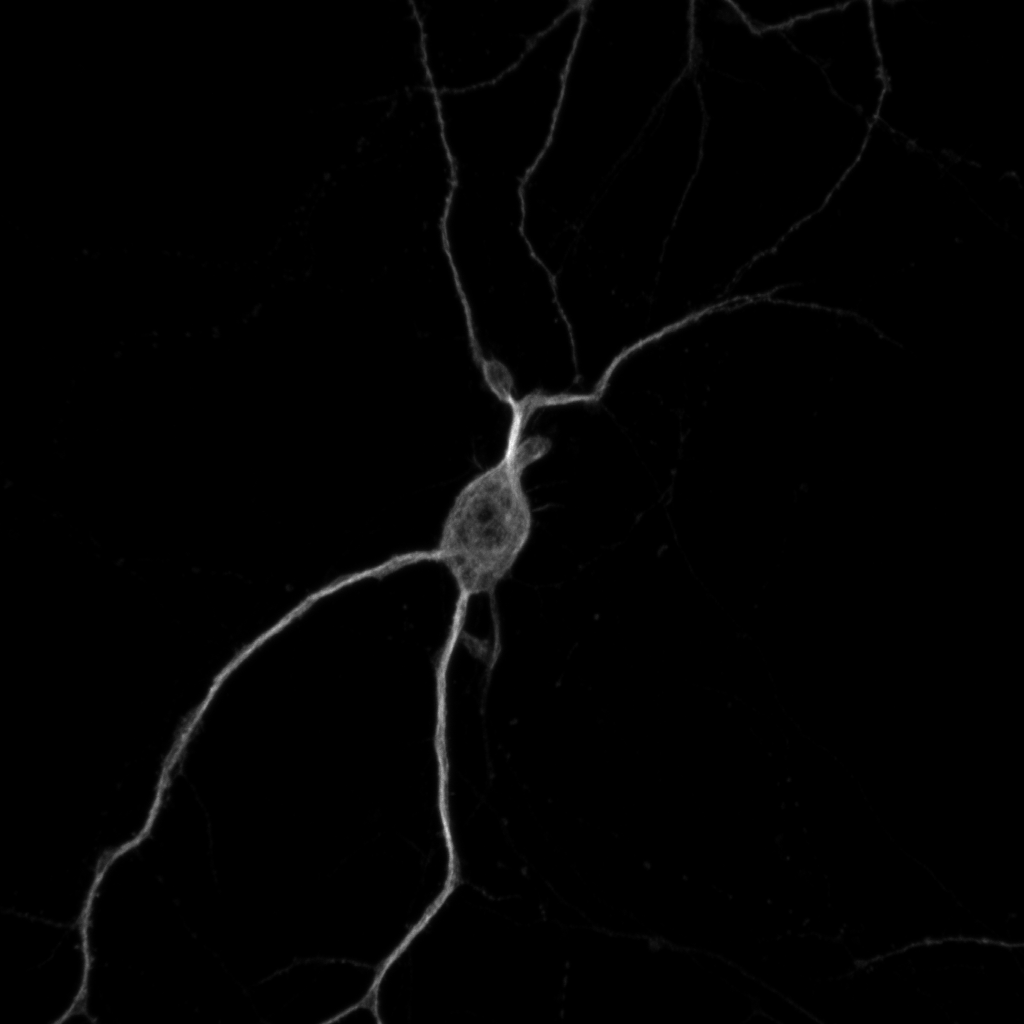

Supplement: Supplementary file 10 — Source data Fig. 8 [file 44321_2024_144_MOESM10_ESM.zip › Figure 8/8E/HET_IFNg_100ng/MAP2_HET_IFNg_100ng.tif]

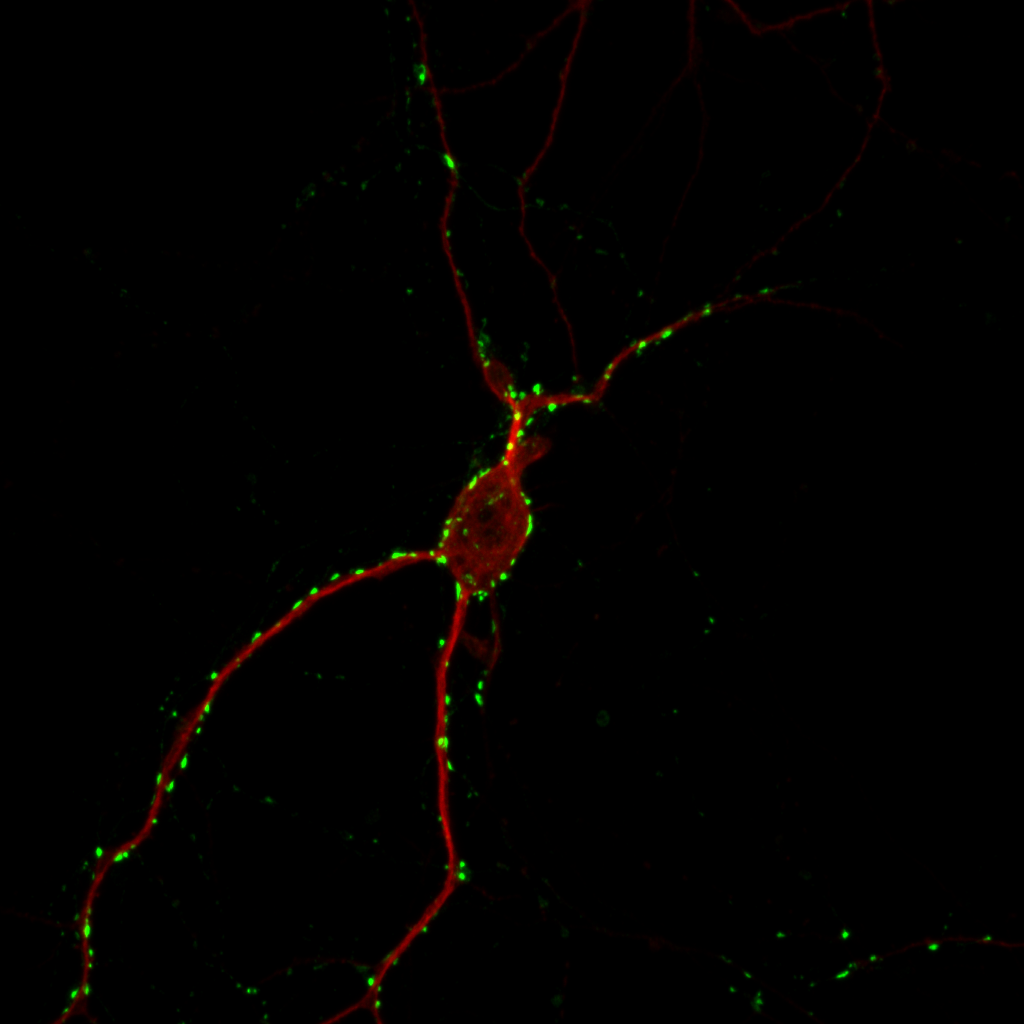

Supplement: Supplementary file 10 — Source data Fig. 8 [file 44321_2024_144_MOESM10_ESM.zip › Figure 8/8E/HET_IFNg_100ng/MERGE_HET_IFNg_100ng.tif]

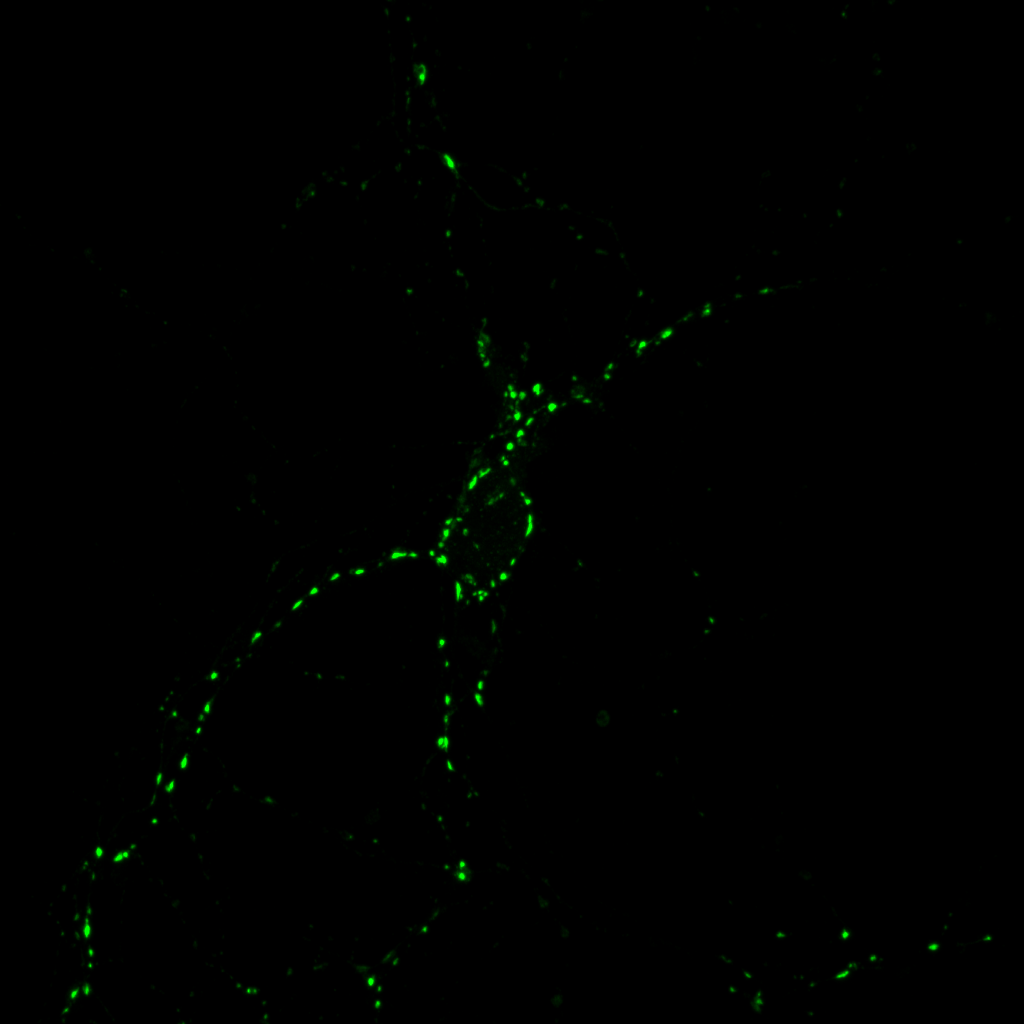

Supplement: Supplementary file 10 — Source data Fig. 8 [file 44321_2024_144_MOESM10_ESM.zip › Figure 8/8E/HET_IFNg_100ng/SYN_HET_IFNg_100ng.tif]

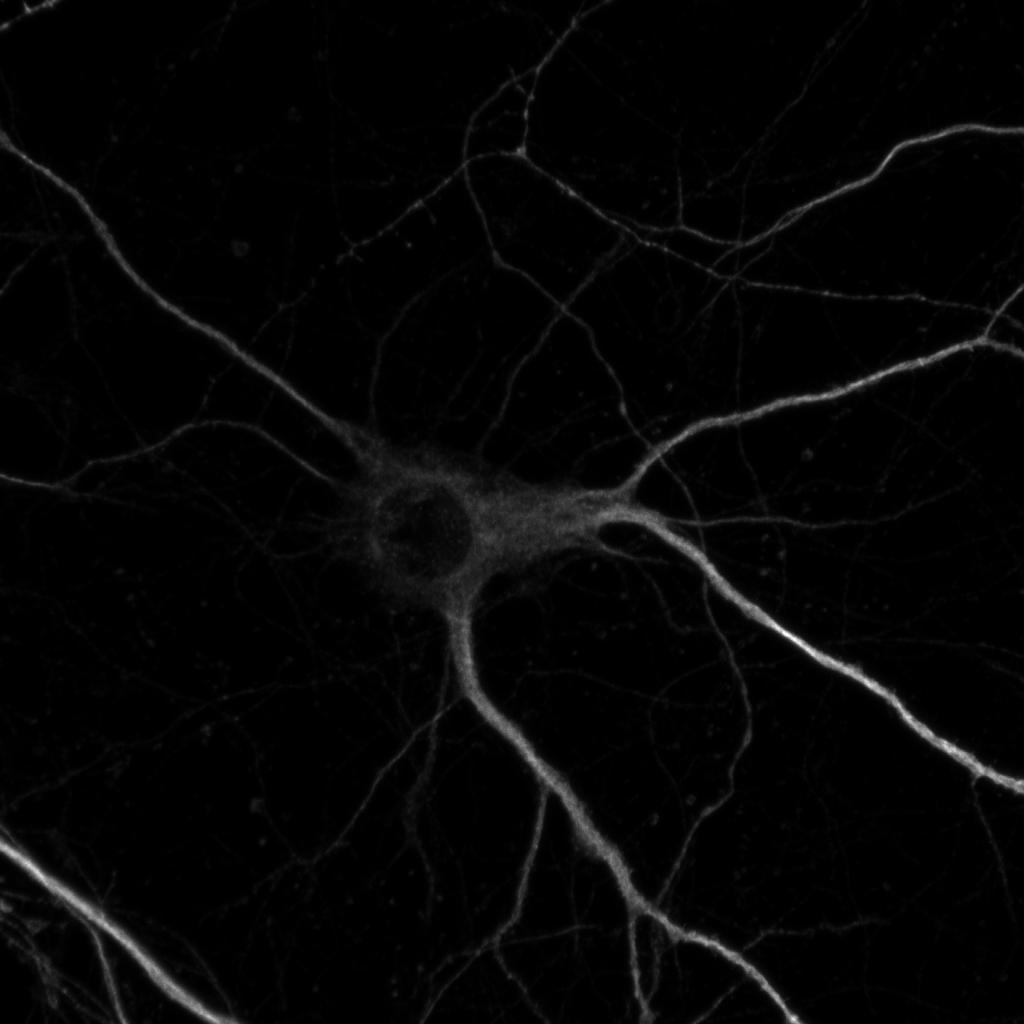

Supplement: Supplementary file 10 — Source data Fig. 8 [file 44321_2024_144_MOESM10_ESM.zip › Figure 8/8E/HET_IFNg_25ng/MAP2_HET_IFNg_25ng.tif]
