# Supplementary figures and images for: Neural precursor cells rescue symptoms of Rett syndrome by activation of the Interferon γ pathway (part 2 of 2)
Source: EMBO Mol Med. 2024 Sep 20;16(12):3218–46. doi: 10.1038/s44321-024-00144-9 (PMC11628625; doi:10.1038/s44321-024-00144-9)

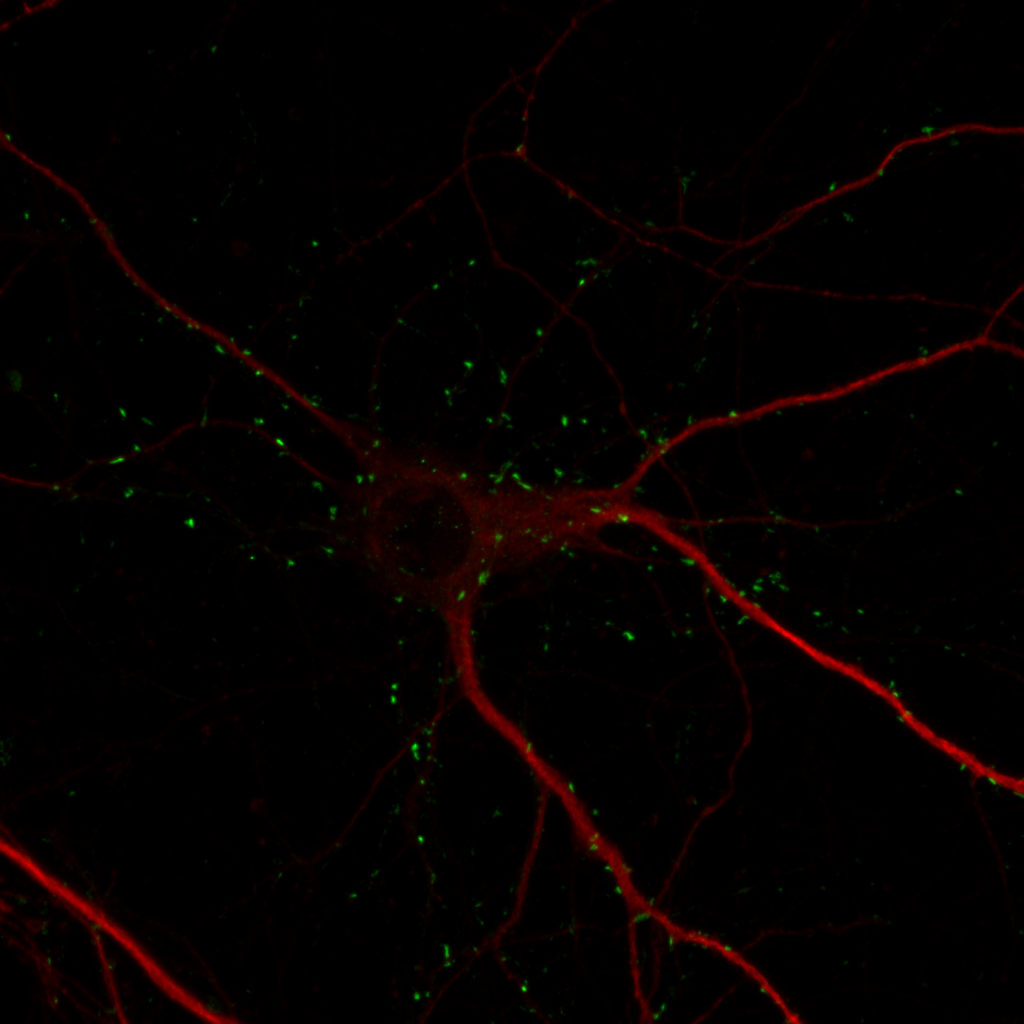

Supplement: Supplementary file 10 — Source data Fig. 8 [file 44321_2024_144_MOESM10_ESM.zip › Figure 8/8E/HET_IFNg_25ng/MERGE_HET_IFNg_25ng.tif]

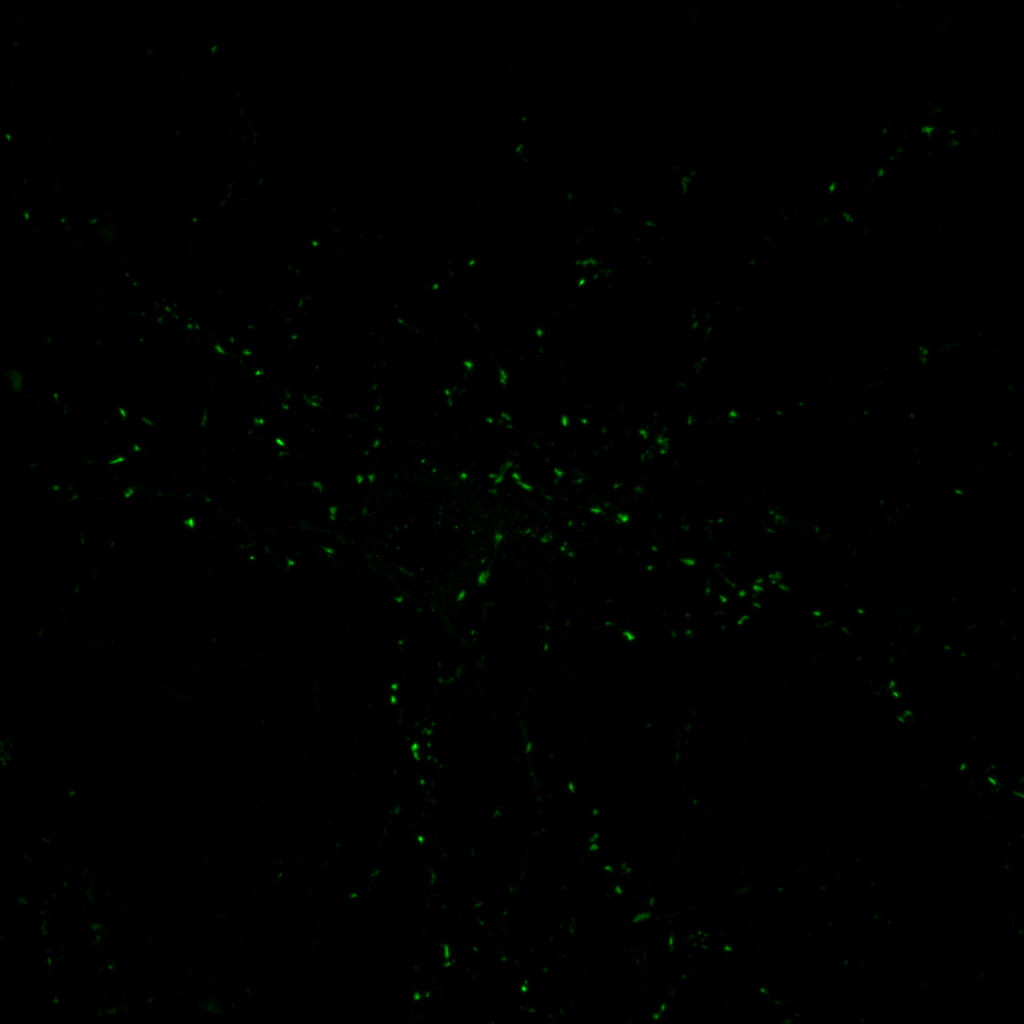

Supplement: Supplementary file 10 — Source data Fig. 8 [file 44321_2024_144_MOESM10_ESM.zip › Figure 8/8E/HET_IFNg_25ng/SYN_HET_IFNg_25ng.tif]

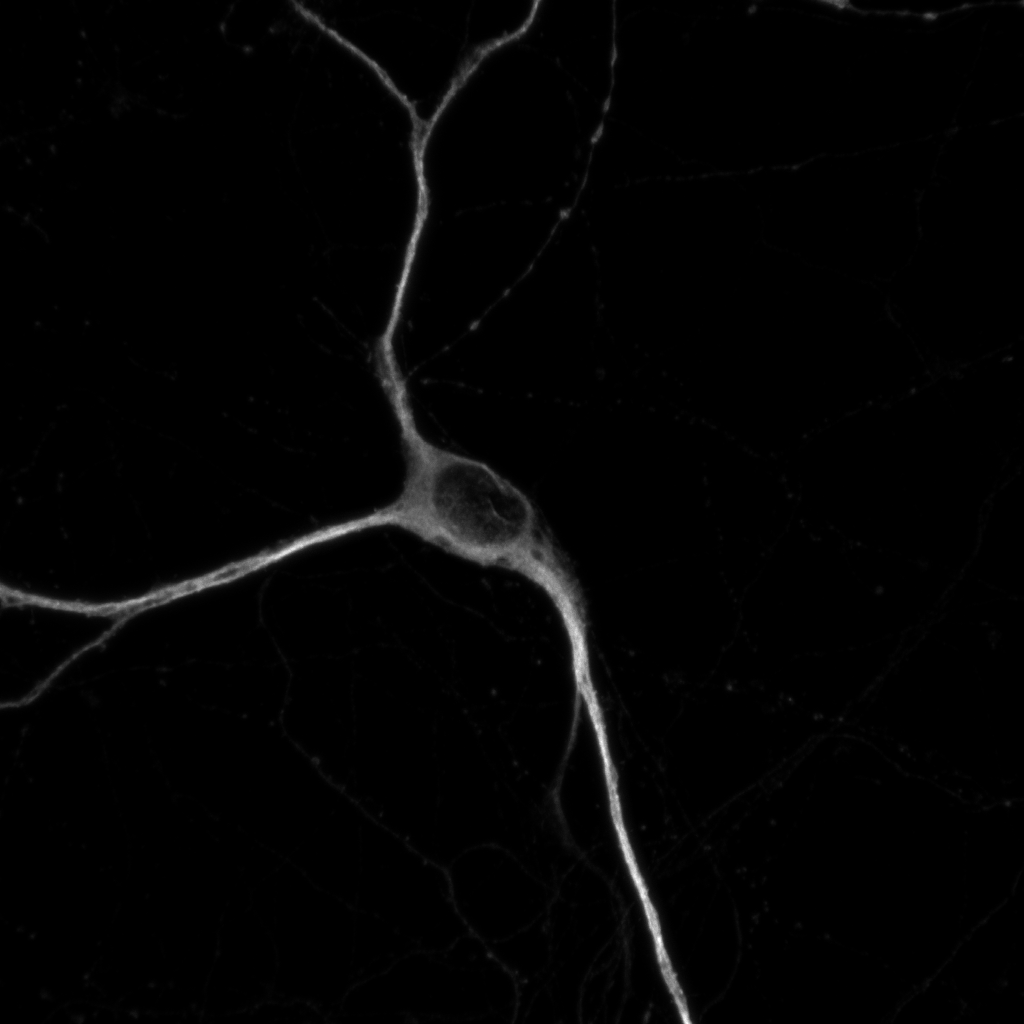

Supplement: Supplementary file 10 — Source data Fig. 8 [file 44321_2024_144_MOESM10_ESM.zip › Figure 8/8E/HET_UT/MAP2_HET_UT.tif]

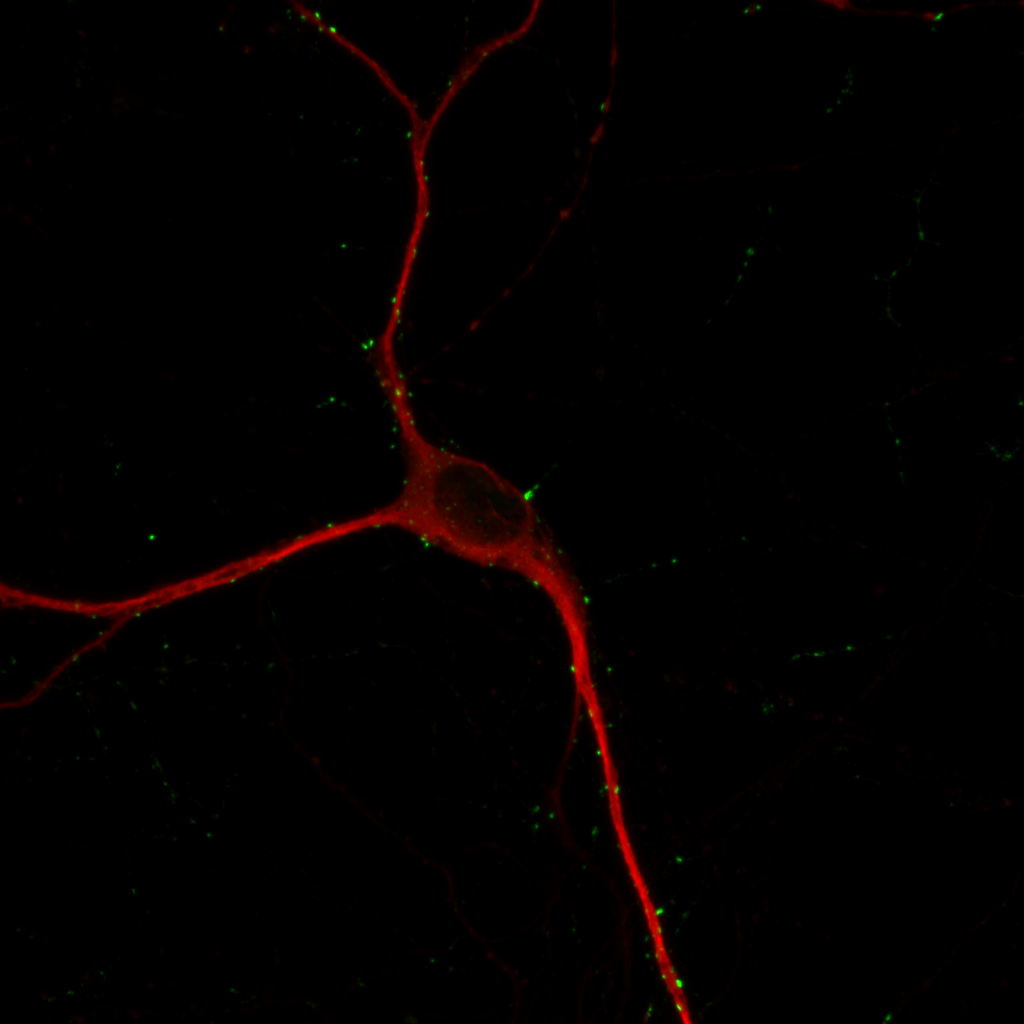

Supplement: Supplementary file 10 — Source data Fig. 8 [file 44321_2024_144_MOESM10_ESM.zip › Figure 8/8E/HET_UT/MERGE_HET_UT.tif]

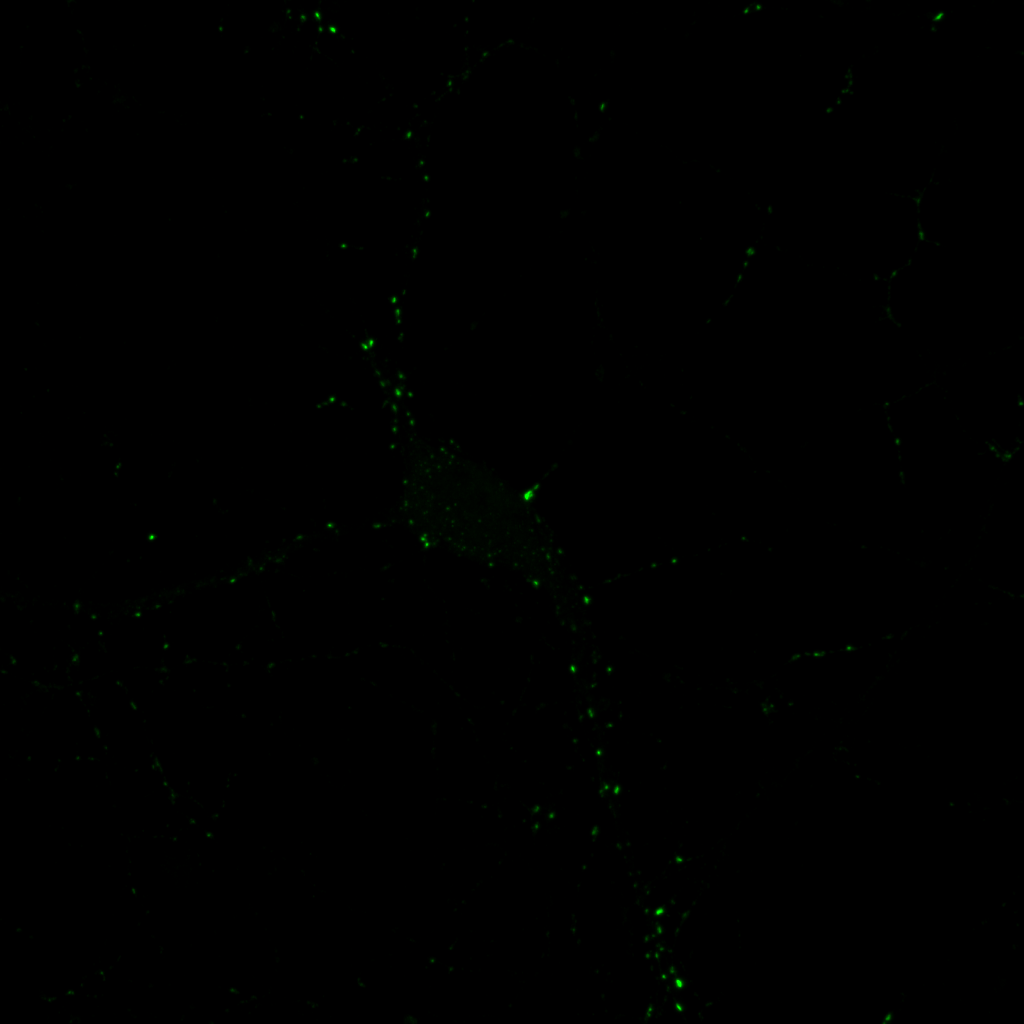

Supplement: Supplementary file 10 — Source data Fig. 8 [file 44321_2024_144_MOESM10_ESM.zip › Figure 8/8E/HET_UT/SYN_HET_UT.tif]

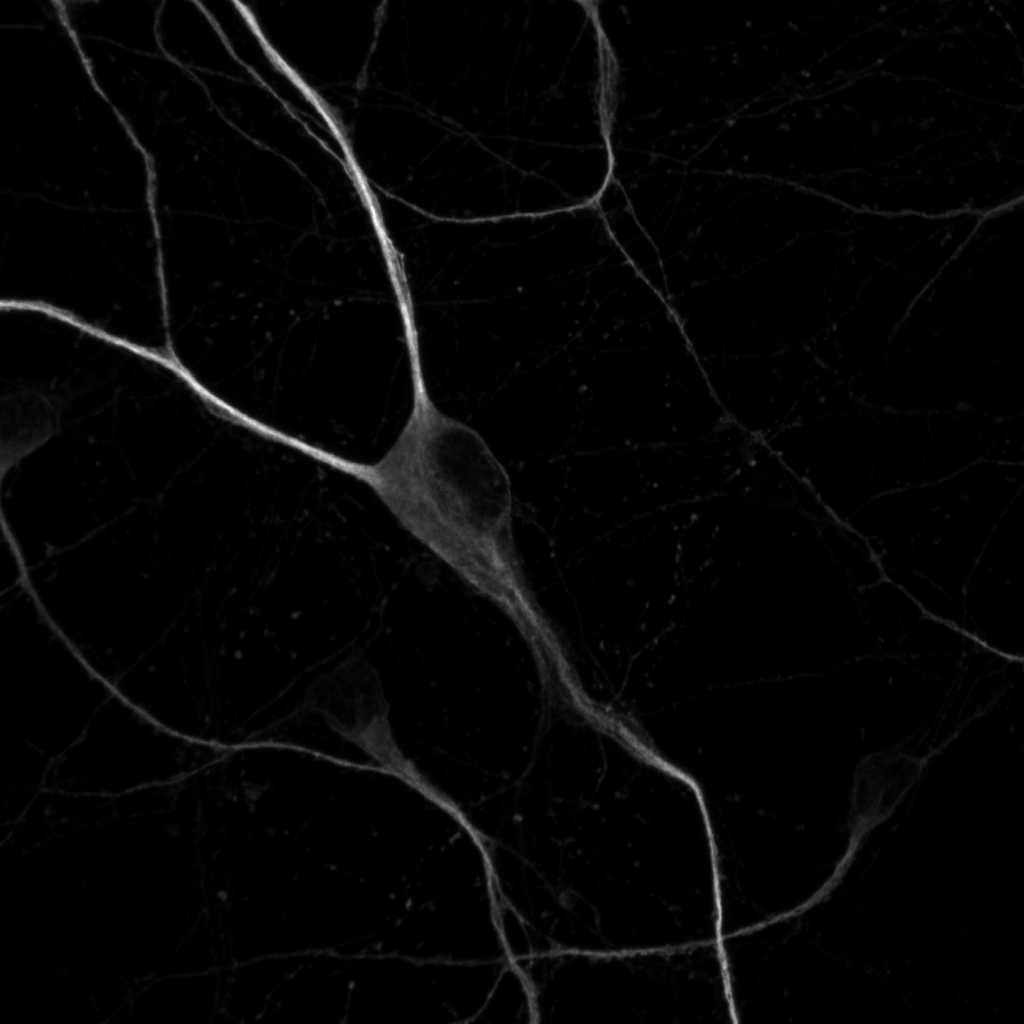

Supplement: Supplementary file 10 — Source data Fig. 8 [file 44321_2024_144_MOESM10_ESM.zip › Figure 8/8E/WT_UT/MAP2_WT_UT.tif]

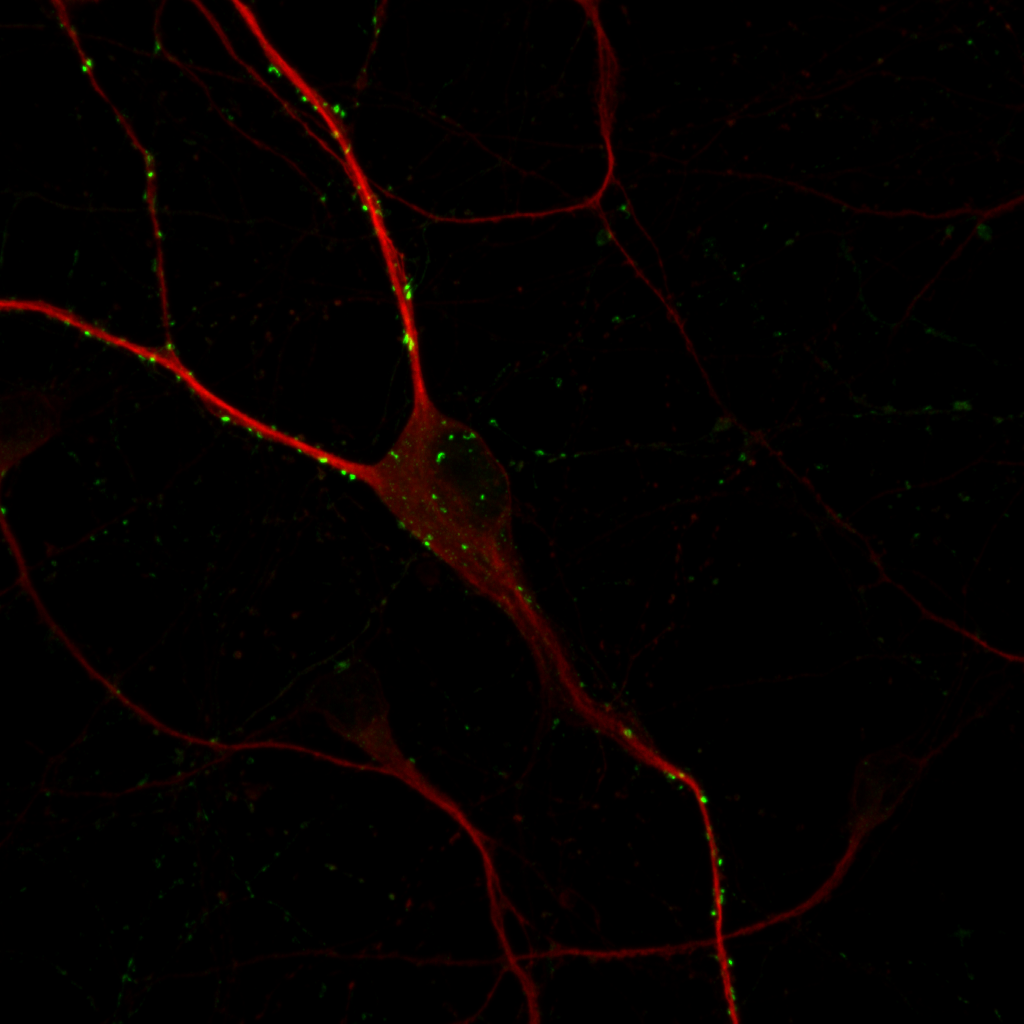

Supplement: Supplementary file 10 — Source data Fig. 8 [file 44321_2024_144_MOESM10_ESM.zip › Figure 8/8E/WT_UT/MERGE_WT_UT.tif]

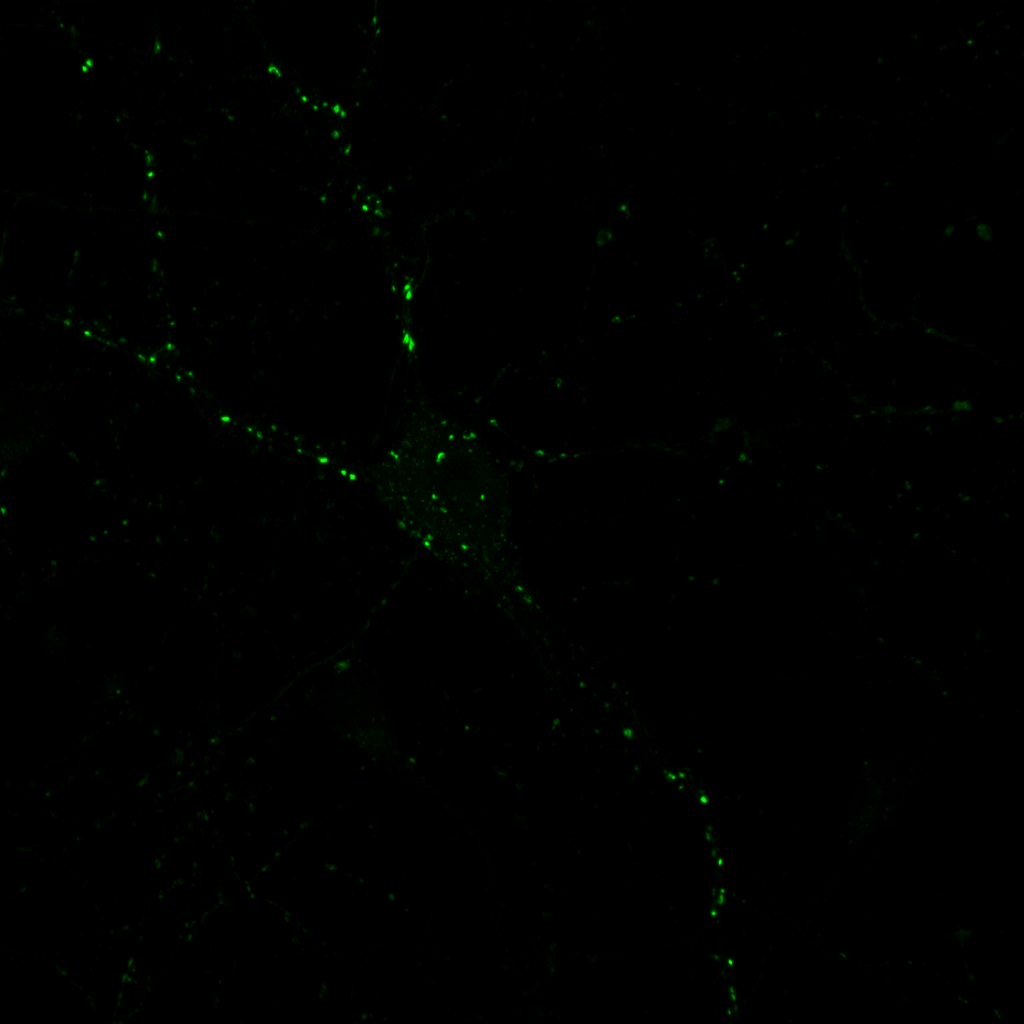

Supplement: Supplementary file 10 — Source data Fig. 8 [file 44321_2024_144_MOESM10_ESM.zip › Figure 8/8E/WT_UT/SYN_WT_UT.tif]
