# Supplementary material for: Neural precursor cells rescue symptoms of Rett syndrome by activation of the Interferon γ pathway
Source: EMBO Mol Med. 2024 Sep 20;16(12):3218–46. doi: 10.1038/s44321-024-00144-9 (PMC11628625; doi:10.1038/s44321-024-00144-9)
Supplement: Supplementary file 12 — Expanded View Figures [file 44321_2024_144_MOESM12_ESM.pdf]

Expanded View Figures

Figure EV1. NPC effects on the morphological and functional properties of neurons.

(**A**) Sholl analysis reports the capacity of NPCs to increase dendritic complexity also in WT neurons. The graph depicts the mean ± SEM of the number of intersections of WT neurons cultured alone, or with NIH3T3 or NPCs from DIV0 to DIV7. *$p = 0.0194$ at 30 μm, *$p = 0.0270$ at 50 μm, *$p = 0.0045$ at 60 μm, *$p = 0.0074$ at 70 μm, *$p = 0.0256$ at 80 μm, *$p = 0.0262$ at 130 μm, *$p = 0.0070$ at 140 μm by two-way ANOVA followed by Tukey post-hoc test. $n = 33$ WT, $n = 17$ WT + NIH3T3; $n = 39$ WT + NPC. (**B**) The histogram shows the mean ± SEM of the total number of intersections calculated by Sholl analysis for WT and KO neurons cultured alone, or in culture with NIH3T3 or NPCs from DIV0 to DIV7. *$p = 0.0135$ WT *vs* KO, *$p = 0.0275$ WT *vs* WT+NPCs, **$p = 0.0032$ KO *vs* KO+NPCs by two-way ANOVA followed by Tukey post-hoc test. $n = 33$ WT, $n = 17$ WT + NIH3T3; $n = 39$ WT + NPC; $n = 39$ KO; $n = 26$ KO + NIH3T3; $n = 32$ KO+NPCs. Neurons derived from at least 3 different mice/genotype. (**C**) Representative traces of excitatory postsynaptic current in miniature (mEPSCs) recorded in primary WT and Het neurons (left) and in Het neurons treated with NPC or NIH3T3 (right). (**D**) Histograms represent the mean ± SEM of the mEPSCs frequency (Hz) and amplitude (pA) both expressed as values normalized on the frequency of WT neurons or amplitude of WT neurons. $n = 26$ WT; $n = 24$ Het; $n = 19$ Het+NPCs; $n = 14$ Het+NIH3T3. Data derived from 3 independent experiments. **$p = 0.0025$ WT *vs* Het by Mann–Whitney test; *$p = 0.0405$ by one-way ANOVA followed by Dunn's test. (**E**) The graph reports the percentage of contamination ± SEM of astrocytes, oligodendrocytes and microglia in cortical neurons at DIV14, independently from the genotype. $n = 3$ for each genotype.

© The Author(s)

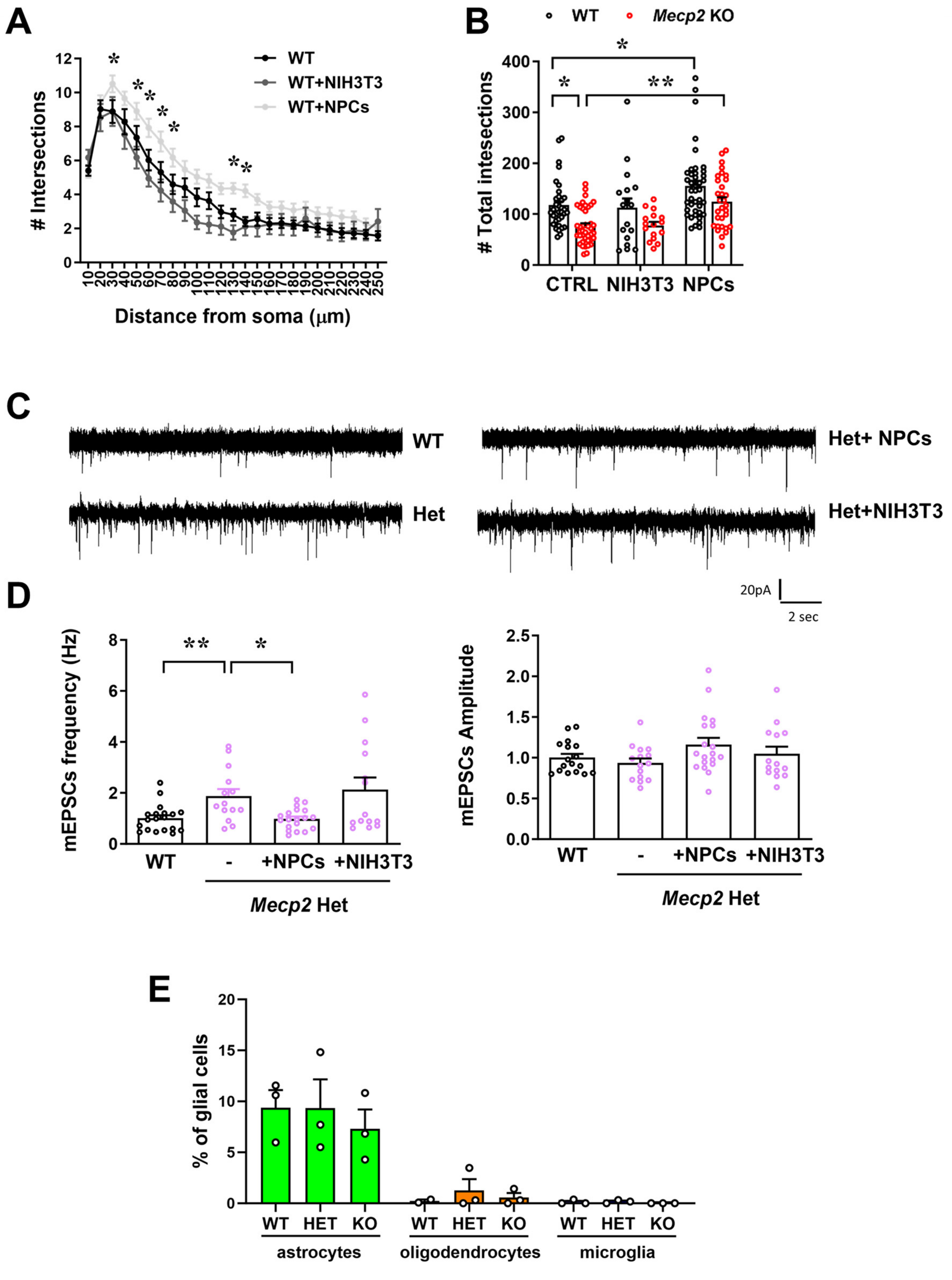
A
Intersections
Distance from soma (μm)
WT
WT+NIH3T3
WT+NPCs
B
WT
Mecp2 KO
Total intesections
CTRL
NIH3T3
NPCs
C
WT
Het
Het+ NPCs
Het+NIH3T3
20pA
2 sec
D
mEPSCs frequency (Hz)
WT
-
+NPCs
+NIH3T3
Mecp2 Het
mEPSCs Amplitude
E
% of glial cells
WT HET KO
astrocytes
oligodendrocytes
microglia

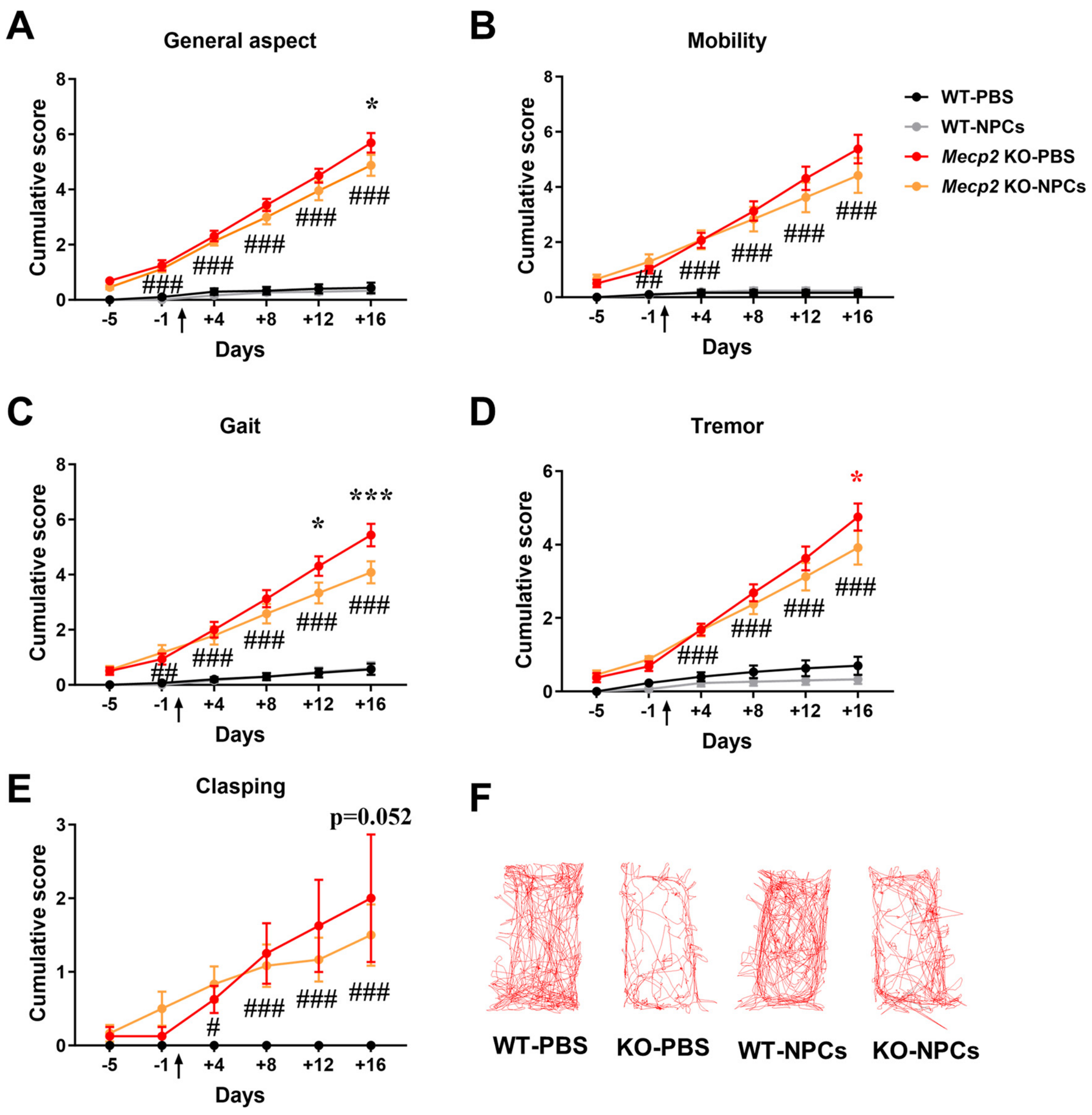

Figure EV2. Phenotypic characterization of NPC-transplanted *Mecp2* KO mice.

(**A–E**) Behavioral scoring was performed by a researcher blind to the treatment, assigning a score between 0 and 2 to general aspect (**A**), mobility (**B**), gait (**C**), tremor (**D**), and clasping (**E**). For each parameter, the graph reports its progression by a cumulative plot, in which the mean ± SEM of each value is obtained by summing the score of each day with those assigned the preceding days. Asterisks indicate a significant difference between KO + PBS and KO+NPCs; hashtags denote a difference between KO + NPC and WT mice. #$p = 0.0140$ for clasping, ##$p = 0.0010$ for gait, ##$p = 0.0022$ for mobility, ###$p < 0.0001$ for parameters, *$p = 0.0144$ for general aspect, *$p = 0.0224$ and ***$p = 0.0005$ for gait, *$p = 0.0367$ for tremor by two-way ANOVA followed by Tukey post-hoc test. $n = 15$ WT + PBS, $n = 15$ WT+NPCs, $n = 9$ KO + PBS, $n = 12$ KO+NPCs. (**F**) Representative traces of the distance travelled during the first day of NOR test by WT + PBS, KO + PBS, WT+NPCs and KO+NPCs mice. The corresponding data are reported in Fig. 4H.

 © The Author(s)

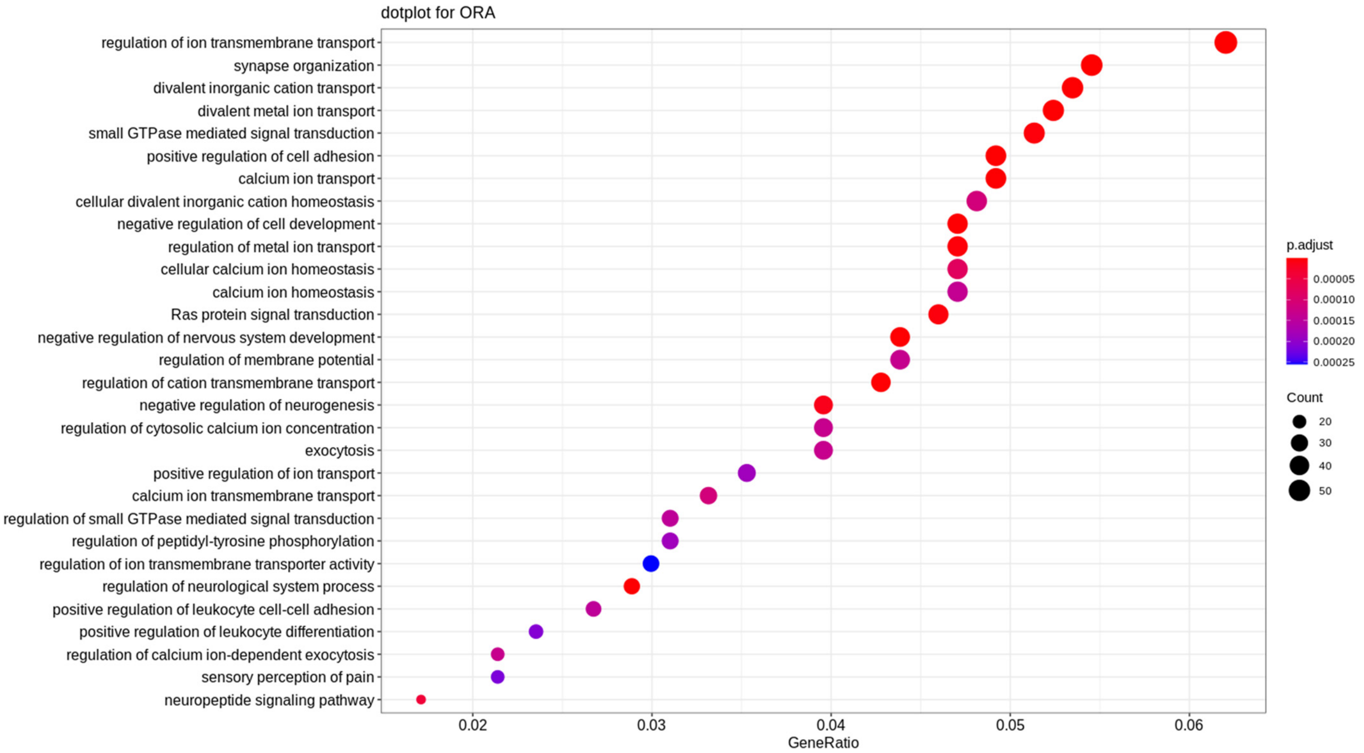

Figure EV3. Gene Ontology (GO) analysis.

(**A**) Dot plot of Gene Ontology (GO) enriched pathway analysis in the cerebellum, indicating the top 30 most enriched pathways of the comparison between KO *versus* WT samples. For a description of the statistical method implemented in ORA see Yu et al, 2012.

A

Region	Contrasts	padj<0.05	padj<0.05 LFC > 1	padj<0.05 LFC < -1
HP	KO vs WT	1214	12	15
HP	KO+NPC vs WT	2074	54	26
HP	KO+NPC vs KO	9	4	0
HP	WT+NPC vs WT	5	0	0

B

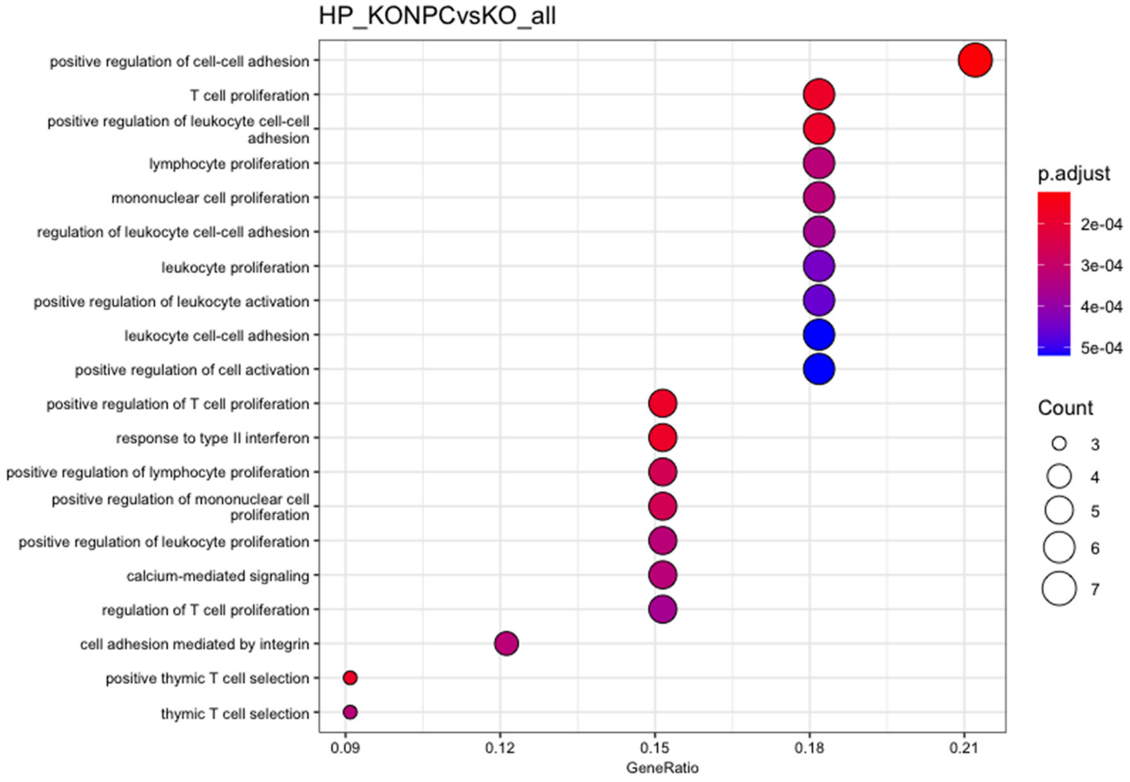

C

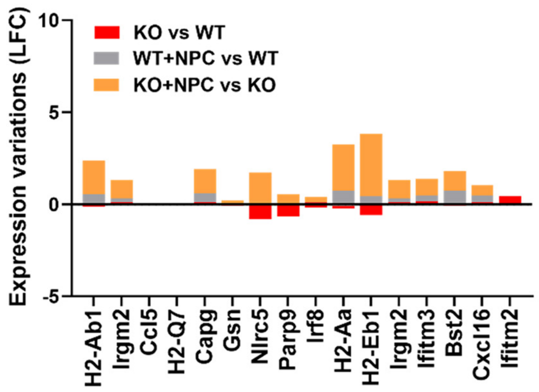

Figure EV4. Bulk RNA-sequencing indicated a mild upregulation of the IFNγ response in the hippocampus of KO mice after NPC transplantation.

(**A**) The table reports the number of deregulated genes (DEGs) for the different comparisons, considering a p.adj < 0.05 and a p.adj < 0.1. The number of DEGs with a LogFoldChange lower than −1 (down-regulated genes) or LogFoldChange greater than 1 (upregulated genes) is also indicated. WT ($n = 6$), KO ($n = 5$), WT+NPCs ($n = 5$) and KO+NPCs ($n = 6$) hippocampi. (**B**) Dot plot of Gene Ontology (GO) indicating the top 20 most enriched pathways of the comparison between KO+NPCs *versus* KO samples. For a description of the statistical method implemented in DESeq2 or ORA see Boyle et al (2004) and Yu et al (2012), respectively. (**C**) The graph shows the LogFoldChange (LFC) of genes belonging to the GO pathway "Interferon-γ response" in the comparisons KO *versus* WT, WT + NPC *versus* WT, and KO+NPCs *versus* KO.

© The Author(s)

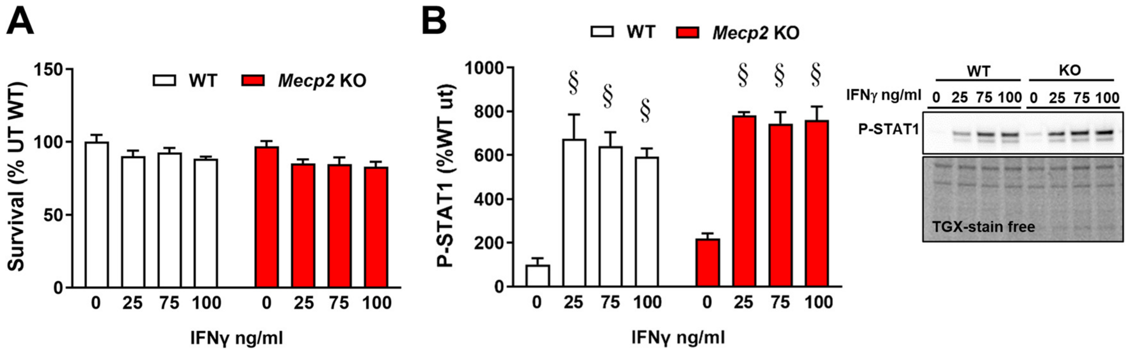

Figure EV5. IFNγ does not affect neuronal survival and activates its downstream kinase in vitro.

(**A**) The histogram represents the cell survival (% untreated WT neurons) ± SEM, assessed by MTT assay. IFNγ was added for 24 h in DIV13 primary neurons and three doses were tested: 25, 75, and 100 ng/ml. (**B**) The histogram reports the mean ± SEM of the levels of phosphorylated STAT1 after IFNγ treatment. Data are normalized to total protein content, visualized by a TGX stain-free technology. Representative bands of phosphorylated STAT1, and the corresponding lanes of TGX-stain-free gel, in WT and KO neurons treated or not with IFNγ are depicted. §$p = 0.0003$ WT-UT *vs* WT 100 ng/ml and §$p < 0.0001$ for the other comparisons, by two-way ANOVA followed by Tukey's post-hoc test. § denotes a significant difference respect to the corresponding untreated control of the same genotype. WT and KO neurons derived from 3 different mice/genotype.
